# Open Issues for Protein Function Assignment in *Haloferax volcanii* and Other Halophilic Archaea

**DOI:** 10.3390/genes12070963

**Published:** 2021-06-24

**Authors:** Friedhelm Pfeiffer, Mike Dyall-Smith

**Affiliations:** 1 Computational Biology Group, Max-Planck-Institute of Biochemistry, 82152 Martinsried, Germany; mike.dyallsmith@gmail.com; 2 Veterinary Biosciences, Faculty of Veterinary and Agricultural Sciences, University of Melbourne, Parkville 3010, Australia

**Keywords:** haloarchaea, genome annotation, Gold Standard Protein, *Haloferax volcanii*, annotation error

## Abstract

Background: Annotation ambiguities and annotation errors are a general challenge in genomics. While a reliable protein function assignment can be obtained by experimental characterization, this is expensive and time-consuming, and the number of such Gold Standard Proteins (GSP) with experimental support remains very low compared to proteins annotated by sequence homology, usually through automated pipelines. Even a GSP may give a misleading assignment when used as a reference: the homolog may be close enough to support isofunctionality, but the substrate of the GSP is absent from the species being annotated. In such cases, the enzymes cannot be isofunctional. Here, we examined a variety of such issues in halophilic archaea (class Halobacteria), with a strong focus on the model haloarchaeon *Haloferax volcanii*. Results: Annotated proteins of *Hfx. volcanii* were identified for which public databases tend to assign a function that is probably incorrect. In some cases, an alternative, probably correct, function can be predicted or inferred from the available evidence, but this has not been adopted by public databases because experimental validation is lacking. In other cases, a probably invalid specific function is predicted by homology, and while there is evidence that this assigned function is unlikely, the true function remains elusive. We listed 50 of those cases, each with detailed background information, so that a conclusion about the most likely biological function can be drawn. For reasons of brevity and comprehension, only the key aspects are listed in the main text, with detailed information being provided in a corresponding section of the Supplementary Materials. Conclusions: Compiling, describing and summarizing these open annotation issues and functional predictions will benefit the scientific community in the general effort to improve the evaluation of protein function assignments and more thoroughly detail them. By highlighting the gaps and likely annotation errors currently in the databases, we hope this study will provide a framework for experimentalists to systematically confirm (or disprove) our function predictions or to uncover yet more unexpected functions.

## 1. Introduction

*Haloferax volcanii* is a model organism for halophilic archaea [1,2,3,4,5,6], for which an elaborate set of genetic tools has been developed [7,8,9]. Its genome has been sequenced and carefully annotated [1,10,11]. A plethora of biological aspects have been successfully tackled in this species, with examples including DNA replication [4]; cell division and cell shape [12,13,14,15,16]; metabolism [17,18,19,20,21,22,23,24,25]; protein secretion [26,27,28,29]; motility and biofilms [30,31,32,33,34,35]; mating [36]; signaling [37]; virus defense [38]; proteolysis [39,40,41,42,43,44]; posttranslational modification (N-glycosylation; SAMPylation) [45,46,47,48,49,50]; gene regulation [21,25,51,52,53,54,55]; microproteins [56,57,58] and small noncoding RNAs (sRNAs) [59,60,61,62].

Genome annotations are frequently compromised by annotation errors [11,63,64,65]. Many of these errors are caused by an invalid annotation transfer between presumed homologs, which, once introduced, are further spread by annotation robots. This problem can be partially overcome by using a Gold Standard Protein (GSP)-based annotation strategy [11]. Since the GSP has itself been subjected to an experimental analysis, its annotation cannot be caused by an invalid annotation transfer process. The GSP strategy was already applied to a detailed analysis of the metabolism of halophilic archaea [66]. However, with a decreasing level of sequence identity, the assumption of isofunctionality becomes increasingly uncertain. Although this may be counterbalanced by additional evidence, e.g., gene clustering, experimental confirmation would be the best option for validation of the annotation.

There are additional and much more subtle genome annotation problems. In some cases, GSPs are true homologs, and the annotated function in the database is correct. Nevertheless, the biological context in the query organism makes it unlikely that the homologs are isofunctional, e.g., when the substrate of the GSP is lacking in the query organism. Additionally, paralogs may have distinct but related functions that cannot be assigned by a sequence analysis but may be assigned based on phylogenetic considerations. Here, again, experimental confirmation is the preferred option for validation of the annotation. A lack of experimental confirmation may keep high-level databases like KEGG or the SwissProt section of UniProt from adopting assignments based on well-supported bioinformatic analyses, so that the database entries continue to provide information that is probably incorrect. We refer to annotation problems in these databases solely to underscore that the biological issues raised by us are far from trivial. There is no intention to question the exceedingly high quality of the SwissProt and KEGG databases [67,68] and their tremendous value for the scientific community. We have actively supported them by providing feedback and encourage others to do the same, e.g., with the recently implemented “Add a publication” functionality in the UniProt entries that allows users to connect a protein to a publication that describes its experimental characterization (https://community.uniprot.org/bbsub/bbsubinfo.html).

In this study, we describe a number of annotation issues for haloarchaea, with a strong emphasis on *Hfx. volcanii*. We denote such cases as “open annotation issues” with the hope of attracting members of the *Haloferax* community and other groups working with halophilic archaea to apply experimental analyses to elucidate the true function(s) of these proteins. This will increase the number of Gold Standard Proteins that originate from *Hfx. volcanii* or other haloarchaea, reduce genome annotation ambiguities and perhaps uncover novel metabolic processes.

## 2. Materials and Methods

### 2.1. Curation of Genome Annotation and Gold Standard Protein Identification

The Gold Standard Protein-based curation of haloarchaeal genomes has been described previously [11] (see, also, next paragraph). Since then, a systematic comparison to the KEGG data was performed for a subset of the curated genomes [69]. The *Hfx. volcanii* genome annotation is continuously scrutinized, especially when a closely related genome is annotated [70]. 

In brief, the core rule of Gold Standard Protein-based genome annotation is to assign a specific function only when a homologous protein has been confirmed experimentally to have this function. Two types of data must be available for that homolog: (a) a reference describing the experimental characterization and (b) an entry in a sequence database, so that the level of sequence similarity can be determined. The decision on whether isofunctionality can be assumed at this level of sequence similarity and, thus, if the annotation can be transferred represents an informed prediction by the annotator based on the available evidence. This decision may be taken only once for a set of closely related orthologs, such as those from halophilic archaea.

### 2.2. Additional Bioinformatics Tools

The key databases were UniProtKB/SwissProt [68], InterPro [71], KEGG [67] and OrthoDB [72]. The SyntTax server was used for inspecting the conservation of the gene neighborhood [73]. As general tools, the BLAST suite of programs [74,75] was used for sequence comparisons. 

## 3. Results

The open issues are organized below under Section 3.1, the respiratory chain and oxidative decarboxylation; Section 3.2, amino acid metabolism; Section 3.3, heme and cobalamin biosynthesis; Section 3.4, coenzyme F420; Section 3.5, tetrahydrofolate as opposed to methanopterin; Section 3.6, NAD and riboflavin; Section 3.7, lipid metabolism; Section 3.8, genetic information processing and Section 3.9, stand-alone (miscellaneous) cases. We collected this set of open annotation issues during our continuous efforts to keep the *Hfx. volcanii* genome up-to-date since its initial publication in 2010 [1]. Not covered in this study are enigmatic reactions and pathways (e.g., archaeal signal peptidase II or the haloarchaeal O-glycosylation pathway) for which no support from experimentally characterized homologs (GSP proteins) is available.

### 3.1. The Respiratory Chain and Oxidative Decarboxylation

In the respiratory chain, the coenzymes that were reduced during catabolism (e.g., glycolysis) are reoxidized, with the energy being saved as an ion gradient. The textbook examples of a respiratory chain are the five mitochondrial complexes [76,77]: complex I (NADH dehydrogenase), complex II (succinate dehydrogenase), complex III (cytochrome bc_1_ complex), complex IV (cytochrome-c oxidase as a prototype for a terminal oxidase) and complex V (F-type ATP synthase). In mitochondria, a significant part of the NADH that feeds into the respiratory chain originates from oxidative decarboxylation: the conversion of pyruvate to acetyl-CoA by the pyruvate dehydrogenase complex and conversion of α-ketoglutarate to succinyl-CoA by the homologous 2-oxoglutarate dehydrogenase complex. While complexes I and II transfer reducing elements to a lipid-embedded two-electron carrier (ubiquinone), the bc_1_ complex transfers the electrons to the one-electron carrier cytochrome-c, a heme (and, thus, iron) protein, which then transfers electrons to the terminal oxidase.

Bacteria like *Escherichia coli* and *Paracoccus denitrificans* have related complexes and enzymes: NADH dehydrogenase (encoded by the *nuo* operon), succinate dehydrogenase (encoded by *sdhABCD*) and the related fumarate reductase (encoded by *frdABCD*) [78], several terminal oxidases (e.g., products of *cyoABCDE* and *cydABC*) and an F-type ATP synthase (encoded by *atp* genes). *E. coli* lacks a bc_1_ complex, which, however, occurs in *Paracoccus denitrificans* [79]. *E. coli* contains the canonical complexes of oxidative decarboxylation (the pyruvate dehydrogenase complex, encoded by *aceEF*+*lpdA*, and the 2-oxoglutarate dehydrogenase complex, encoded by *sucAB*+*lpdA*).

The respiratory chain of *Hfx. volcanii* and other haloarchaea deviates considerably from those of mitochondria and bacteria such as *Paracoccus* and *E. coli* (reviewed by [80]), and a number of questions remain unresolved. We focus on the equivalents of complexes I, III and IV, because these have unresolved issues. We also cover some aspects relevant for the NADH levels (oxidative decarboxylation enzymes and type II NADH dehydrogenase). We do not cover complexes that have already been studied in haloarchaea: complex II (succinate dehydrogenase) [81,82,83] and complex V (ATP synthase) [84,85]. 

(a) In haloarchaea, oxidative decarboxylation is not linked to the reduction of NAD to NADH but to the reduction of a ferredoxin (encoded by *fdx*, e.g., OE_4217R, HVO_2995), which has a redox potential similar to that of the NAD/NADH pair [86]. The enzymes for oxidative decarboxylation are pyruvate–ferredoxin oxidoreductase (*porAB*, e.g., OE_2623R/2622R and HVO_1305/1304) and 2-oxoglutarate–ferredoxin oxidoreductase (*korAB*, e.g., OE_1711R/1710R and HVO_0888/0887), and these have been characterized from *Halobacterium salinarum* [87,88,89].

(b) It is yet unresolved how ferredoxin Fdx is reoxidized, but this might be achieved by the Nuo complex. This ferredoxin may well be involved in additional metabolic processes. In *Hfx. volcanii*, ferredoxin Fdx (HVO_2995) plays an essential role in nitrate assimilation [90]. However, in *Hbt. salinarum*, this metabolic process for Fdx reoxidation does not exist. 

(c) The *nuo* cluster of haloarchaea resembles that of *E. coli*, a type I NADH dehydrogenase, with the genes and gene order highly conserved and just a few domain fissions and fusions. However, haloarchaea lack NuoEFG [91], which is a subcomplex that mediates interaction with NADH [92,93]. Thus, the haloarchaeal *nuo* complex is unlikely to function as NADH dehydrogenase, despite its annotation as such in KEGG (as of April 2021).

(d) Other catabolic enzymes generate NADH, which must also be reoxidized. Based on inhibitor studies, NADH is not reoxidized by a type I but, rather, by a type II NADH dehydrogenase in *Hbt. salinarum* [82]. A tentative gene assignment has been made for *Natronomonas pharaonis* [66]. However, for reasons detailed in Supplementary Text S1 Section S1, this assignment is highly questionable, so this issue calls for an experimental analysis.

(e) About one-third of the haloarchaea, especially the *Natrialbales*, do not code for a complex III equivalent (the cytochrome bc_1_ complex encoded by *petABC*), according to OrthoDB analysis. The bc_1_ complex is required to transfer electrons from the lipid-embedded two-electron carrier (menaquinone in haloarchaea) to the one-electron carrier associated with terminal oxidases (probably halocyanin). How electrons flow in the absence of a complex III equivalent is currently unresolved.

The haloarchaeal *petABC* genes resemble those of the chloroplast b6-f complex rather than those of the mitochondrial bc_1_ complex (see Supplementary Text S1 Section S1 for more details).

(f) A bc cytochrome was purified from *Nmn. pharaonis*, but with an atypical 1:1 ratio between the b-type and c-type hemes [81]. The complex is heterodimeric, with subunits of 18 kDa and 14 kDa. The 18-kDa subunit carries the covalently attached heme group [81]. An attempt was made to identify the genes coding for these subunits [94] (for details, see Supplementary Text S1 Section S1). Two approaches were used to obtain protein sequence data, one being the N-terminal protein sequencing of the two subunits extracted from a SDS-polyacrylamide gel. In the other attempt, peptides from the purified complex were separated by HPLC, and a peptide which absorbed at 280 nm (protein), as well as 400 nm (heme), was isolated. Absorption at 400 nm clearly indicates that the isolated peptide contains a covalently attached heme group. The sequences from the two approaches overlapped and resulted in a contiguous sequence of 41 aa, with only the penultimate position remaining undefined [94]. Based on this information, a PCR probe was generated (designated “cyt-C Sonde”) that allowed the gene to be identified and sequenced, including its genomic neighborhood. It turned out that the genes coding for the four subunits of succinate dehydrogenase (*sdhCDBA*) were isolated. The obtained protein sequence corresponds to the N-terminal region of *sdhD* (with the initiator methionine cleaved off) and only two sequence discrepancies, in addition to the unresolved penultimate residue.

In the PhD thesis [94], this unambiguous result was rated to be a failure (and the data were never formally published). The reason is that SdhD is free of cysteine residues, while standard textbooks state that a pair of cysteines is required for covalent heme attachment [95]. The lack of the required cysteine pair was taken to indicate that the results were incorrect and that the identified genes did not encode the cytochrome bc that the study was seeking [94]. In contrast, we speculate that the results were completely correct, despite being in conflict with the cysteine pair paradigm. In our opinion, a paradigm shift is required. The obtained results call for a yet-unanticipated novel mode of covalent heme attachment, exemplified by the 18-kDa subunit of *Natronomonas* succinate dehydrogenase subunit SdhD. It should be noted that the 41-aa protein sequence, which was obtained, turned out to contain three histidine residues upon translation of the gene, but none of these were detected upon Edman degradation.

In *Halobacterium*, a small c-type cytochrome was purified (cytochrome c_552_, 14.1 kDa) [96]. Heme staining after SDS-PAGE indicated a covalent heme attachment, but no sequence or composition data were reported, so that it was not possible to identify the protein based on the available information. We speculate that the *Halobacterium* cytochrome c_552_ also represents SdhD (as detailed in Supplementary Text S1 Section S1). In that case, the proposed novel type of covalent heme attachment would not be restricted to *Nmn. pharaonis* but might be a general property of haloarchaea. This would also solve the “*Halobacterium* paradox” [95].

(g) The haloarchaeal one-electron carrier is the copper protein halocyanin rather than the iron-containing heme protein cytochrome-c. A halocyanin from *Nmn. pharaonis* (NP_3954A) was characterized, including its redox potential [97,98,99]. A gene fusion supports the close connection of a halocyanin with a subunit of a terminal oxidase. For further details, see Supplementary Text S1 Section S1.

(h) Terminal oxidases are highly diverse in haloarchaea, and we restricted our analysis to three species (*Nmn. pharaonis*, *Hfx. volcanii* and *Hbt. salinarum*), because in each of these, at least one terminal oxidase has been experimentally studied (Table 1). The details are described in Supplementary Text S1 Section S1 with subunits of all analyzed terminal oxidases listed in Supplementary Table S1.

(i) NAD-dependent oxidative decarboxylation is a canonical reaction to convert pyruvate into acetyl-CoA and α-ketoglutarate into succinyl-CoA. In haloarchaea, the conversion of pyruvate to acetyl-CoA and α-ketoglutarate to succinyl-CoA is dependent on ferredoxin, not on NAD (see above). Nevertheless, most haloarchaeal genomes also code for homologs of enzymes catalyzing NAD-dependent oxidative decarboxylation, such as the *E. coli* pyruvate dehydrogenase complex. In most cases, the substrates could not be identified, an exception being a paralog involved in isoleucine catabolism [116]. In several cases, the enzymes were found not to show catalytic activity with pyruvate or α-ketoglutarate (see Supplementary Text S1 Section S1 for details). Additionally, a conditional lethal *porAB* mutant was unable to grow on glucose or pyruvate, thus excluding that alternative enzymes for the conversion of pyruvate to acetyl-CoA exist in *Hfx. volcanii* [22]. Nonetheless, despite experimental results to the contrary, pyruvate has been assigned as a substrate for some of the homologs of the pyruvate dehydrogenase complex in KEGG (as of April 2021). 

### 3.2. Amino Acid Metabolism

While most amino acid biosynthesis and degradation pathways can be reliably reconstructed, a few open issues remain, which are discussed below.

(a) The first and last steps of arginine biosynthesis deal with blocking and unblocking of the α-amino group of the substrate (glutamate) and a product intermediate (ornithine). As detailed in Supplementary Text S1 Section S2, it is highly likely that glutamate is attached to the γ-carboxyl group of a carrier protein, and ornithine is released from that carrier protein. This is based on characterized proteins from *Thermus thermophilus* [124], *Thermococcus kodakarensis* [125] and *Sulfolobus acidocaldarius* [126]. The assignment is strongly supported by clustering of the arginine biosynthesis genes. Some of the homologs are bifunctional, being involved in arginine biosynthesis but, also, in lysine biosynthesis via the prokaryotic variant of the α-aminoadipate pathway. This ambiguity is not assumed to occur in haloarchaea, which use the diaminopimelate pathway for lysine biosynthesis [127] (see Supplementary Text S1 Section S2 for further discussion of this issue).

Expanding the above, we provided full details underlying our reconstruction of arginine and lysine biosynthesis in *Hfx. volcanii* in Table 2.

**Table 2:** Proteins with open annotation issues and their Gold Standard Protein homologs (Section 3.2). For a description of this table, see the legend to Table 1.

			Gold Standard Protein						
Section	Code	Gene	isofunc	%seq_id	Locus tag	UniProt	Reference	PMID	Comment
2a	HVO_0047	*argW*	no	54%	TT_C1544	Q72HE5	[128]	25392000	for Arg, not for Lys biosynthesis
2a	HVO_0047 (cont.)		yes/no	39%	Saci_0753	Q4JAQ0			only for Arg, not for Lys biosynthesis
2a	HVO_0047 (cont.)		yes/no	61%	TK0279	Q5JFV9	[125]	27566549	only for Arg, not for Lys biosynthesis
2a	HVO_0046	*argX*	no	44%	TT_C1543	Q72HE6	[124]	19620981	for Arg, not for Lys biosynthesis
2a	HVO_0046 (cont.)		yes	30%	Saci_1621	Q4J8E7			only for Arg, not for Lys biosynthesis
2a	HVO_0046 (cont.)		yes/no	37%	TK0278	Q5JFW0	[125]	27566549	only for Arg, not for Lys biosynthesis
2a	HVO_0044	*argB*	no	41%	TT_C1541	O50147	[124] [128]	19620981 25392000	for Arg, not for Lys biosynthesis
2a	HVO_0044 (cont.)		yes/no	33%	Saci_0751	Q4JAQ2	[126]	23434852	only for Arg, not for Lys biosynthesis
2a	HVO_0044 (cont.)		yes/no	32%	TK0276	Q5JFW2	[125]	27566549	only for Arg, not for Lys biosynthesis
2a	HVO_0045	*argC*	no	48%	TT_C1542	O50146	[124] [129]	19620981 26966182	for Arg, not for Lys biosynthesis
2a	HVO_0045 (cont.)		yes/no	42%	Saci_0750	Q4JAQ3	[126]	23434852	only for Arg, not for Lys biosynthesis
2a	HVO_0045 (cont.)		yes/no	46%	TK0277	Q5JFW1	[125]	27566549	only for Arg, not for Lys biosynthesis
2a	HVO_0043	*argD*	no	45%	TT_C1393	Q93R93	[130]	11489859	for Arg, not for Lys biosynthesis
2a	HVO_0043 (cont.)		yes/no	40%	Saci_0755	Q4JAP8	[126]	23434852	only for Arg, not for Lys biosynthesis
2a	HVO_0043 (cont.)		yes/no	42%	TK0275	Q5JFW3	[125]	27566549	only for Arg, not for Lys biosynthesis
2a	HVO_0042	*argE*	no	36%	TT_C1396	Q8VUS5	[124] [131]	19620981 28720495	for Arg, not for Lys biosynthesis
2a	HVO_0042 (cont.)		yes/no	29%	Saci_0756	Q4JAP7	[126]	23434852	only for Arg, not for Lys biosynthesis
2a	HVO_0042 (cont.)		yes/no	37%	TK0274	Q5JFW4	[125]	27566549	only for Arg, not for Lys biosynthesis
2a	HVO_0041	*argF*	yes	50%	P18186	BSU11250	[132]	4216455	
2a	HVO_0041 (cont.)		yes	47%	OE_5205R	B0R9X3	[133]	7868583	
2a	HVO_0049	*argG*	yes	35%	-	P00966	[134]	8792870	human
2a	HVO_0049 (cont.)		yes	23%	b3172	P0A6E4	[135]	10666579	*E. coli*
2a	HVO_0048	*argH*	yes	38%	MMP0013	O74026	[136]	10220900	
2a	HVO_0008	*lysC*	yes	32%	BSU28470	P08495	[137]	15033471	
2a	HVO_2487	*asd*	yes	51%	MJ0205	Q57658	[138]	16225889	
2a/9e	HVO_1101	*dapA*	yes	45%	PA1010	Q9I4W3	[139]	21396954	
2a	HVO_1100	*dapB*	yes	33%	b0031	P04036	[140]	7893644	
2a	HVO_1099	*dapD*	yes	32%	b0166	P0A9D8	[141]	6365916	
2a	HVO_1096	*dapE*	yes	29%	b2472	P0AED7	[142]	3276674	function supported by gene clustering
2a	HVO_1097	*dapF*	yes	35%	b3809	P0A6K1	[143]	6378903	
2a	HVO_1098	*lysA*	yes	38%	b2838	P00861	[144]	14343156	
2a	HVO_A0634	-	unknown	25%	b2472	P0AED7	[142]	3276674	function assigned to HVO_1096 in *dap* cluster
2b	HVO_0790	*fba2*	special	67%	OE_1472F	B0R334	[145]	25216252	EC 2.2.1.10 activity of OE_1472F not yet confirmed in vitro
2b	HVO_0790 (cont.)		special	45%	MJ0400	Q57843	[146]	15182204	substrate uncertain
2b	HVO_0792	*aroB*	yes	69%	OE_1475F	B0R336	[145]	25216252	OE_1475F only partially characterized
2b	HVO_0792 (cont.)		yes	44%	MJ1249	Q58646	[146]	15182204	
2b	HVO_0602	*aroD1*	yes	44%	OE_1477R	B0R338	[145]	25216252	
2b	HVO_0602 (cont.)		yes	31%	MMP1394	Q6LXF7	[147]	15262931	
2c	HVO_0009	*tnaA*	yes	41%	b3708	P0A853	[148] [149]	2659590 14284727	
2d	HVO_A0559	*hutH*	yes	42%	BSU39350	P10944	[150] [151]	2454913 14066617	
2d	HVO_A0562	*hutU*	yes	62%	BSU39360	P25503	[152]	4990470	
2d	HVO_A0560	*hutI*	yes	42%	BSU39370	P42084	[153]	16990261	
2d	HVO_A0561	*hutG*	yes	33%	BSU39380	P42068	[152]	4990470	
2e	HVO_0431	-	-						no GSP available
2e	HVO_0644	*leuA1*	yes/no	47%	MJ1392	Q58787	[154]	9864346	HVO_0644 monofunc (CimA) or bifunc (CimA+LeuA); MJ1392 CimA
2e	HVO_0644 (cont.)		unclear	44%	MJ1195	Q58595	[155]	9665716	HVO_0644 monofunc (CimA) or bifunc (CimA+LeuA); MJ1195 LeuA
2e/2f	HVO_1510	*leuA2*	yes	47%	MJ1195	Q58595	[155]	9665716	HVO_1510 LeuA; MJ1195 LeuA
2e/2f	HVO_1510 (cont.)		no	41%	MJ1392	Q58787	[154]	9864346	HVO_1510 LeuA MJ1392 CimA
2e	HVO_A0489	*-*	no	31%	MJ1392	Q58787	[154]	9864346	HVO_A0489 general function only; MJ1392 CimA
2e	HVO_A0489 (cont.)		no	30%	MJ1195	Q58595	[155]	9665716	HVO_A0489 general function only; MJ1195 LeuA
2e	HVO_1153	-	-						function unassigned; no GSP

(b) Archaea use a different precursor for aromatic amino acid biosynthesis than the classical pathway. This has been resolved for *Methanocaldococcus jannaschii* and for *Methanococcus maripaludis* [146,156]. However, the initial steps may differ from those reported for *Methanocaldococcus* in that fructose 1,6-bisphosphate, rather than 6-deoxy-5-ketofructose, might be a substrate [145]. Up to now, a clean deletion of the corresponding enzymes and confirmation with in vitro assays has not yet been achieved (for details, see Supplementary Text S1 Section S2).

(c) The gene for tryptophanase (*tpa*) is stringently regulated in *Haloferax*, which is the basis for using its promoter in the toolbox for regulated gene expression [157]. The shutdown of this gene avoids tryptophan degradation when supplies are scarce. Tryptophanase cleaves tryptophan into indole, pyruvate and ammonia. The fate of indole is, however, yet unresolved.

(d) A probable histidine utilization cluster exists, based on the characterized homologs from *Bacillus subtilis*, but has not yet been experimentally verified.

(e) Among the 16 auxotrophic mutants observed in a *Hfx. volcanii* transposon insertion library [9], some could grow only in the presence of one (or several) supplied amino acids. In many cases, the affected genes were known to be involved in the corresponding pathway, but the others may lead to novel function assignments. One affected gene resulted in histidine auxotrophy, and the product of this gene (HVO_0431) is an interesting candidate. The InterPro domain assignment (HAD family hydrolase) fits into the only remaining pathway gap in histidine biosynthesis (histidinol-phosphatase). In this context, it should be noted that the enzyme that catalyzes the preceding reaction (encoded by *hisC*) is part of a highly conserved three-gene operon involved in polar lipid biosynthesis (see below). For details, see Supplementary Text S1 Section S2. One affected gene resulted in isoleucine auxotrophy. The product of this gene (HVO_0644) is currently annotated to catalyze two reactions, one being an early step in isoleucine biosynthesis (EC 2.3.1.182) and the other being the first step after leucine biosynthesis branches off from valine biosynthesis (EC 2.3.3.13) (see below, (f)) (for details, see Supplementary Text S1 Section S2).

(f) *Hfx. volcanii* codes for two paralogs with an attributed function as 2-isopropylmalate synthase (EC 2.3.3.13). This is the first reaction specific to leucine biosynthesis when the pathway branches off valine biosynthesis. One paralog, HVO_0644, is annotated as bifunctional, also catalyzing a chemically similar reaction that is an early step in isoleucine biosynthesis (EC 2.3.1.182). When the gene encoding HVO_0644 is disrupted by transposon integration, cells cannot grow in the absence of isoleucine. It is unclear if the protein is really bifunctional and is really involved in leucine biosynthesis, catalyzing the reaction of EC 2.3.3.13. The other paralog, HVO_1510, belongs to an ortholog set with major problems concerning the start codon assignment. The ortholog set from the 16 genomes listed in Supplementary Table S2 was analyzed. When only canonical start codons are considered (ATG, GTG and TTG), the orthologs from *Haloferax mediterranei*, *Nmn. pharaonis*, *Natronomonas moolapensis* and *Halohasta litchfieldiae* either lack a long highly conserved N-terminal region or they are disrupted (pseudogenes), being devoid of a potential start codon. The gene from *Hfx. volcanii* has a start codon (GTG) that is consistent with that of *Haloferax gibbonsii* strain LR2-5 (but a GTA in *Hfx. gibbonsii* strain ARA6). In this region, the gene from *Hfx. mediterranei* is closely related but has in-frame stop codons. HVO_1510 is considerably longer than the orthologs from *Haloquadratum walsbyi*, *Haloarcula hispanica* and *Natrialba magadii*. The first alternative start codon for HVO_1510 codes for Met-93. This protein was proteomically identified in three ArcPP datasets [2], and peptides upstream of Met-93 were identified. This gene might be translated from an atypical start codon, either an in-frame CTG or an out-of-frame ATG, which would require ribosomal slippage (for details, see Supplementary Text S1 Section S2 and Supplementary Figure S1). It is tempting to speculate that translation occurs only when leucine is not available.

### 3.3. Coenzymes I: Cobalamin and Heme

**Figure 1:**
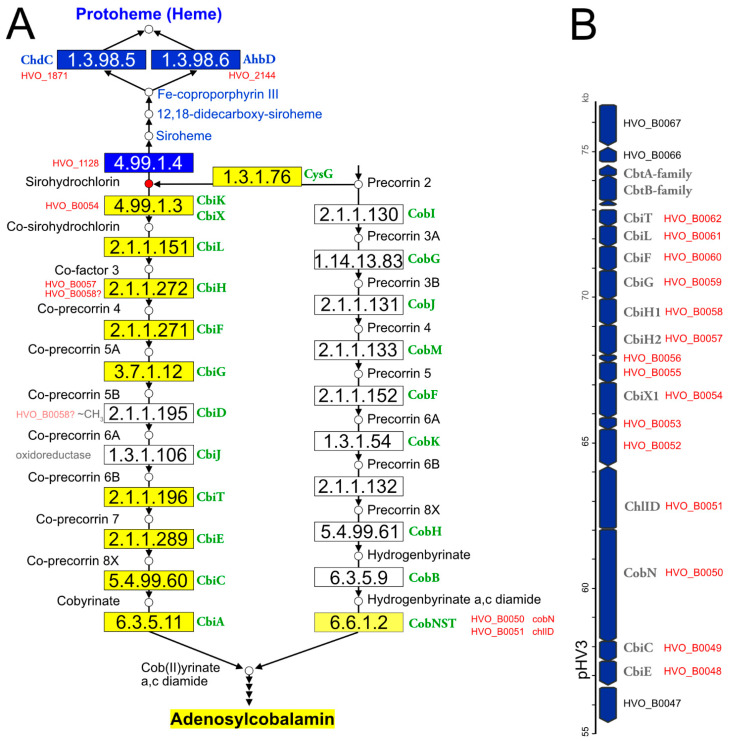
Illustration of the haloarchaeal cobalamin and heme biosynthesis pathways and of the major cobalamin biosynthesis gene cluster. (**A**) Biosynthesis pathways. This illustration is based on the corresponding KEGG map 00860. Small circles represent pathway intermediates and have their names assigned. Pathway intermediates upstream of precorrin-2 are not displayed. The circle for sirohydrochlorin is highlighted in red, as this is the branchpoint for heme and cobalamin biosynthesis in haloarchaea. Enzymatic reactions are shown by arrows, the EC numbers being provided in rectangular boxes. Rectangles are colored when the enzyme has been reconstructed for haloarchaea (blue: heme biosynthesis; dark yellow: de novo cobalamin biosynthesis; light yellow: late cobaltochelatase, which may be a salvage reaction). Gene names in green are adopted from KEGG and represent those from bacterial model pathways. Consecutive arrowheads indicate reaction series that are not shown in detail for space reasons. Additionally, some enzymes of the heme biosynthesis pathway are omitted for space reasons. For enzymatic reactions that are considered to be open issues, *Hfx. volcanii* locus tags are provided. For two pathway gaps (white boxes in the cobalt-early pathway), the type of reaction is indicated (oxidoreductase and ~CH3, indicating a methylation reaction). The question mark after HVO_B0058 indicates that this protein, currently co-attributed to EC 2.1.1.272, is a candidate for the yet-unassigned EC 2.1.1.195 reaction. We note that haloarchaea might use a deviating biosynthesis pathway, e.g., by swapping the methylation and oxidoreductase reactions (not illustrated). (**B**) The major cobalamin cluster, encoded on megaplasmid pHV3. Arrows are used to indicate the coding strand and are roughly drawn to scale. If assigned, the gene name is provided in addition to the *Hfx. volcanii* locus tag. Locus tags in red indicate genes that are part of the cobalamin cluster.

The classical heme biosynthesis pathway branches off cobalamin biosynthesis at the level of uroporphyrinogen III. A second pathway exists in bacteria (CPD pathway). Haloarchaea use the alternative heme biosynthesis pathway [158], which has an additional common step with cobalamin biosynthesis, the conversion of uroporphyrinogen III to precorrin-2. For heme biosynthesis, precorrin-2 is converted into siroheme. This pathway was reconstructed [159], except for the iron insertion step. For de novo cobalamin biosynthesis, haloarchaea use the cobalt-early pathway. A key reaction in this pathway variant, catalyzed by CbiG, is cobalt-dependent. Thus, cobalt must be inserted early and is present in all intermediates [160]. Several aspects of heme and cobalamin biosynthesis in haloarchaea have yet to be resolved. This is illustrated in Figure 1.

(a) *Hfx. volcanii* contains two annotated *cbiX* genes. For the reasons detailed in Supplementary Text S1 Section S3, we predict that one is a cobaltochelatase, involved in cobalamin biosynthesis, while the other is a ferrochelatase, responsible for the conversion of precorrin-2 to siroheme in the alternative heme biosynthesis pathway. 

(b) De novo cobalamin biosynthesis has been extensively reconstructed upon curation of the genome annotation [11]. All enzymes of the pathway and their associated GSPs are listed in Table 3. Only two pathway gaps remained, and because these are consecutive, it may be possible that the haloarchaeal pathway is noncanonical and proceeds via a novel biosynthetic intermediate. There are only four genes with yet-unassigned functions in the *Hfx. volcanii* cobalamin gene cluster, and their synteny is well-conserved in the majority of haloarchaeal genomes. Thus, these genes are obvious candidates for filling the pathway gaps (for details, see Supplementary Text S1 Section S3). 

(c) The cobalamin biosynthesis and salvage reactions (those beyond ligand cobyrinate a,c diamide) involve “adenosylation of the corrin ring, attachment of the aminopropanol arm, and assembly of the nucleotide loop that bridges the lower ligand dimethylbenzimidazole and the corrin ring” [161]. The enzymes of these branches of cobalamin biosynthesis and their associated GSPs are listed in Table 3. Only two pathway gaps remain open. For one of these, a candidate was proposed upon a detailed bioinformatic analysis [161] (for further details, see Supplementary Text S1 Section S3).

(d) In the cobalt-late (aerobic) pathway variant, the intermediates are cobalt-free, and cobalt is inserted only late in the pathway. Even though haloarchaea do not use the cobalt-late pathway, so that a late cobaltochelatase is not required, they code for a homolog of the large subunit of a characterized heterotrimeric late cobaltochelatase. The adjacent gene is homologous to small subunits of other chelatases. We speculate that this late cobaltochelatase may be involved in cobalamin salvage. The chelatase has a mosaic subunit structure, as also reported previously [161] (see Supplementary Text S1 Section S3 for details).

(e) In the alternative heme biosynthesis pathway, siroheme is decarboxylated to 12,18-didecarboxysiroheme, which is attributed to the proteins encoded by *ahbA* and *ahbB*. These are homologous to each other and are organized as two two-domain proteins. It is unclear if AhbA and AhbB function independently or if they form a complex.

(f) Two of the three heme biosynthesis pathways (AHB and CPD) share a common last step (decarboxylation of Fe-coproporphyrin III to protoheme (heme b)). They use, however, distinct types of enzymes (AHB: *ahbD*, EC 1.3.98.6, adenosylmethionine-dependent heme synthase, a radical SAM enzyme; CPD: *chdC*, EC 1.3.98.5, peroxide-dependent heme synthase). Nearly all haloarchaea contain a *chdC* gene, and two-thirds also contain an *ahbD* gene. *Hfx. volcanii* was shown to use AhbD under anaerobic conditions and ChdC under aerobic conditions [162].

**Table 3:** Proteins with open annotation issues and their Gold Standard Protein homologs (Section 3.3). For a description of this table, see the legend to Table 1.

			Gold Standard Protein						
Section	Code	Gene	Isofunc	%seq_id	Locus Tag	UniProt	Reference	PMID	Comment
3a	HVO_B0054	*cbiX1*	yes	30%	-	O87690	[163]	12408752	cobaltochelatase
3a	HVO_B0054 (cont.)		yes	27%	MTH_1397	O27448	[164]	12686546	cobaltochelatase
3a	HVO_1128	*cbiX2*	no	29%	AF0721	O29537	[165]	16835730	cobaltochelatase
3a	HVO_1128 (cont.)		no	28%	MTH_1397	O27448	[164]	12686546	cobaltochelatase
3a	HVO_1128 (cont.)		no	29%	AF0721	O29537	[165]	16835730	cobaltochelatase
3a	NP_0734A	*cbiX3*	-						function unassigned; no GSP; distantly related to paralogs
3a	HVO_2312	*sirC*	yes/no	31%	Mbar_A1461	Q46CH4	[166]	21197080	precorrin-2 DH; no analysis for Fe-chelatase
3a	HVO_2312 (cont.)		yes/no	29%	STM3477	P25924	[167] [168]	14595395 32054833	matches to the N-term domain which is bifunctional as precorrin-2 DH and Fe-chelatase
3a	HVO_2312 (cont.)		yes/no	29%	-	P61818	[163] [169]	12408752 18588505	precorrin-2 DH; devoid of Fe-chelatase activity
3b	HVO_B0061	*cbiL*	no	32%	STM2024	Q05593	[170]	1451790	equivalent reaction on cobalt-free substrate
3b	HVO_B0057	*cbiH2*	yes	45%	-	O87689	[160]	23922391	corresponds to N-term of O87689 which has a C-term extension
3b	HVO_B0057 (cont.)		no	40%	STM2027	Q05590	[171] [172]	9331403 16198574	equivalent reaction on cobalt-free substrate
3b	HVO_B0058	*cbiH1*	special	32%	-	O87689	[160]	23922391	corresponds to N-term of O87689 which has a C-term extension; more distant to O87689 than CbiH2
3b	HVO_B0058 (cont.)		no	30%	STM2027	Q05590	[171] [172]	9331403 16198574	equivalent reaction on cobalt-free substrate
3b	HVO_B0060	*cbiF*	no	40%	STM2029	P0A2G9	[170] [173]	1451790 16866557	equivalent reaction on cobalt-free substrate
3b	HVO_B0060 (cont.)		yes	38%	-	O87686	[160]	23922391	
3b	HVO_B0059	*cbiG*	yes	24%	-	O87687	[160]	23922391	
3b	pathway gap								EC 2.1.1.195
3b	pathway gap								EC 1.3.1.106
3b	HVO_B0062	*cbiT*	yes	36%	-	O87694	[160]	23922391	corresponds to the C-term of bifunctional O87694
3b	HVO_B0048	*cbiE*	yes	28%	-	O87694	[160]	23922391	corresponds to the N-term of bifunctional O87694
3b	HVO_B0049	*cbiC*	yes	33%	-	O87692	[160]	23922391	
3b	HVO_A0487	*cbiA*	no	37%	STM2035	P29946	[174]	15311923	equivalent reaction on cobalt-free substrate
3b	HVO_B0052	-	-						function unassigned; no GSP
3b	HVO_B0053	-	-						function unassigned; no GSP
3b	HVO_B0055	-	-						function unassigned; no GSP
3b	HVO_B0056	-	-						function unassigned; no GSP
3c	HVO_A0488	*cobA*	yes	31%	MM_3138	Q8PSE1	[175]	16672609	
3c	HVO_A0488 (cont.)		yes	30%	STM1718	P31570	[176]	12080060	
3c	HVO_2395	*pduO*	yes	37%	-	Q9XDN2	[177]	11160088	PduO and CobA are isofunctional; In Q9XDN2, the PduO domain (N-term) is fused to a DUF336 domain
3c	HVO_A0553	*cbiP*	yes	63%	VNG_1576G OE_3246F	Q9HPL5 B0R5X2	[178]	14645280	
3c	HVO_0587	*cbiB*	yes	58%	VNG_1578H OE_3253F	Q9HPL3 B0R5X4	[178]	14645280	
3c	HVO_0592	*cbiZ*	yes	57%	VNG_1583C OE_3261F	Q9HPL3 B0R5X8	[179]	14990804	
3c	HVO_0589	*cobY*	yes	47%	VNG_1581C OE_3257F	Q9HPL1 B0R5X6	[180]	12486068	
3c	HVO_0588	*cobS*	yes	30%	STM2017	Q05602	[181]	17209023	
3c	-				STM0643	P39701	[182]	7929373	EC 3.1.3.73; CobC; no homolog in haloarchaea
3c	HVO_0586	*-*	prediction	-	-	-	[161]	12869542	EC 3.1.3.73; prediction for HSL01294 (VNG_1577C)
3c	pathway gap								EC 2.7.1.177
3c	HVO_0591	*cobD1*	yes	31%	STM0644	P97084	[183]	9446573	
3c	HVO_0593	*cobD2*	yes						no GSP; 51% seq_id to HVO_0591 (*cobD1*)
3c	HVO_0590	*cobT*	prediction				[161]	12869542	prediction for VNG_1572C
3c	halTADL_3045	*cobT*	yes	39%	STM0644	Q05603	[184]	8206834	
3d	HVO_B0051	*cobN*	yes	34%	-	P29929	[185]	1429466	
3d	HVO_B0051 (cont.)		no	29%	-	Q55284	[186] [187]	8663186 9716491	Mg chelatase
3d	HVO_B0050	*chlID*	no	46%	slr1030	P51634	[186] [187]	8663186 9716491	match to N-term; Mg chelatase
3d	HVO_B0050 (cont.)		no	33%	slr1777	P52772	[186] [187]	8663186 9716491	match to complete sequence, incl distant match to N-term; Mg chelatase
3e	HVO_2227	*ahbA*	yes	35%	-	I6UH61	[158]	21969545	
3e	HVO_2313	*ahbB*	yes	32%	-	I6UH61	[158]	21969545	
3f	HVO_1121	*ahbC*	yes	47%	Mbar_A1793	Q46BK8	[158] [188]	21969545 24669201	
3f	HVO_2144	*ahbD*			self		[162]	29284023	EC 1.3.98.6
3f	HVO_2144 (cont.)		yes	42%	Mbar_A1458	Q46CH7	[188]	24669201	
3f	HVO_1871	*chdC*			self		[162]	29284023	EC 1.3.98.5
3f	HVO_1871 (cont.)		yes	46%	BSU37670	P39645	[189]	28123057	

### 3.4. Coenzymes II: Coenzyme F420

Even though coenzyme F420 is predominantly associated with methanogenic archaea [190,191], it occurs also in bacteria, and a small amount of this coenzyme has been detected in non-methanogenic archaea, including halophiles [192]. The genes required for the biosynthesis of this coenzyme are encoded in haloarchaeal genomes, but the origin and attachment of the phospholactate moiety are not completely resolved (see below). To the best of our knowledge, only a single coenzyme F420-dependent enzymatic reaction has yet been reported for halophilic archaea [193]. Thus, the importance of this coenzyme in haloarchaeal biology is currently enigmatic and awaits experimental analysis.

(a) The pathway that creates the carbon backbone of this coenzyme has been reconstructed. We list the enzymes with their associated GSPs in Table 4. Coenzyme F420 contains a phospholactate moiety, which was reported to originate from 2-phospho-lactate [194], but this compound is metabolically not well-connected. As summarized in Supplementary Text S1 Section S4, there are various new insights regarding this pathway from recent studies in other prokaryotes [195,196]. To the best of our knowledge, the haloarchaeal coenzyme F420 biosynthesis pathway has never been experimentally analyzed.

(b) The prediction of coenzyme F420-specific oxidoreductases in *Mycobacterium* and actinobacteria has been reported [197], leading to patterns and domains that are also found in haloarchaea. Several such enzymes are described in Supplementary Text S1 Section S4.

(c) HVO_1937 might be a coenzyme F420-dependent 5,10-methylenetetrahydrofolate reductase (see, also, below: C1 metabolism, and Supplementary Text S1 Section S4).

(d) The precursor for coenzyme F420 may be used by a photolyase involved in DNA repair.

**Table 4:** Proteins with open annotation issues and their Gold Standard Protein homologs (Section 3.4). For a description of this table, see the legend to Table 1.

			Gold Standard Protein						
Section	Code	Gene	Isofunc	%seq_id	Locus Tag	UniProt	Reference	PMID	Comment
4a	HVO_2198	*cofH*	yes	35%	MJ1431	Q58826	[198] [199]	14593448 25781338	
4a	HVO_2201	*cofG*	yes	43%	MJ0446	Q57888	[198] [200] [199]	14593448 23072415 25781338	
4a	HVO_2202	*cofC*	yes	25%	MJ0887	Q58297	[194] [195] [196]	18260642 30952857 31469543	
4a	HVO_2479	*cofD*	yes	39%	MM_1874	Q8PVT6	[201] [196]	18252724 31469543	
4a	HVO_2479 (cont.)		yes	32%	MJ1256	Q58653	[202]	11888293	
4a	HVO_1936	*cofE*	yes	47%	AF_2256	O28028	[203]	17669425	
4a	HVO_1936 (cont.)		yes	38%	MJ0768	Q58178	[204]	12911320	
4b	HVO_0433	*npdG*	yes	38%	AF_0892	O29370	[205]	not in PubMed	
4b	HVO_B0113	-	no	27%	Rv0132c	P96809	[206]	24349169	too distant to assume isofunctionality
4b	HVO_B0342	-	unknown	29%	-	O93734	[207] [208]	8706724 15016352	too distant to assume isofunctionality
4b	NP_1902A	-	no	28%	-	Q9UXP0	[209] [210]	1735436 9933933	too distant to assume isofunctionality
4b	NP_4006A	-	no	27%	MJ0870	Q58280	[211]	16048999	too distant to assume isofunctionality
4c/5c	HVO_1937	*mer*	no	38%	MTH_1752	O27784	[212] [213] [214]	2298726 7649177 10891279	
4d	HVO_2911	*phr2*	yes	62%	VNG_1335G OE_2907R	Q9HQ46 B0R5D6	[215] [216]	2681164 12773185	
4d	HVO_2843	*phr1*	no	45%	sll1629	P77967	[217]	12535521	sll1629 implicated in transcription regulation
4d	HVO_2843 (cont.)		possibly	45%	At5g24850	Q84KJ5	[218] [219]	12834405 17062752	mediates photo-repair of ssDNA
4d	HVO_1234	*phr3*	possibly	40%	Atu4765	A9CH39	[220]	23589886	

### 3.5. Coenzymes III: Coenzymes of C1 Metabolism: Tetrahydrofolate in Haloarchaea and Methanopterin in Methanogens

**Figure 2:**
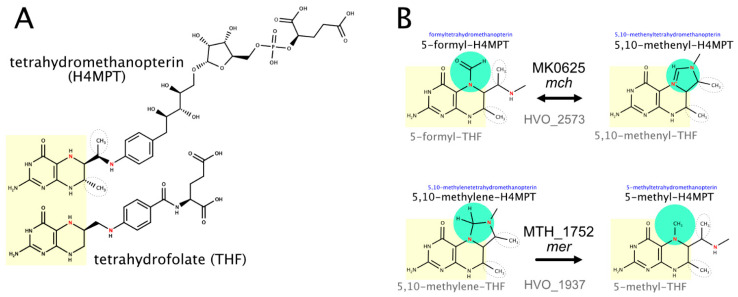
The structure of the C1 coenzymes tetrahydrofolate and methanopterin and two enzymes that act on the attached C1 compound. (**A**) The structures of tetrahydromethanopterin (top) and tetrahydrofolate (bottom) illustrate the similarities and differences between these C1 coenzymes. The common pteridine-based ring system is highlighted in yellow, and the initial biosynthesis step that generates this ring system is catalyzed by homologous enzymes (topic (b)). Two methanopterin-specific methyl groups are outlined by dashed ovals. N5 and N10, which are involved in the binding of the C1 compound, are colored red. (**B**) Two enzymatic reactions that alter the oxidation level of the C1 compound are illustrated. The methanogenic and haloarchaeal enzymes are homologous, even though they use distinct C1 coenzymes (topic (c)). It should be noted that MTH-1752 uses coenzyme F420 (not illustrated, Section 3.4, topic (c)), and this might also hold true for HVO_1937.

Halophilic and methanogenic archaea use distinct coenzymes as one-carbon carriers (C1 metabolism): tetrahydrofolate in haloarchaea and methanopterin in methanogens [221,222]. Several characterized methanogenic proteins that act on or with methanopterin have comparably close homologs in haloarchaea (Table 5), which results in the misannotation of haloarchaeal proteins (e.g., in SwissProt) as being involved in methanopterin biology. We assume that the haloarchaeal proteins function with the haloarchaeal one-carbon carrier tetrahydrofolate and that this shift in coenzyme specificity is possible due to the structural similarity between methanopterin and tetrahydrofolate (a near-identical core structure consisting of a pterin heterocyclic ring linked via a methylene bridge to a phenyl ring) (Figure 2). A detailed review on the many variants of the tetrahydrofolate biosynthetic pathway is available [223].

(a) Folate biosynthesis requires aminobenzoate. We proposed candidates for a pathway from chorismate to para-aminobenzoate [66,224] (for details, see Supplementary Text S1 Section S5). However, these predictions have not been adopted by KEGG (accessed April 2021), and without experimental confirmation, this is unlikely to ever happen.

(b) GTP cyclohydrolase MptA (HVO_2348) catalyzes a reaction in the common part of tetrahydrofolate and methanopterin biosynthesis. The enzymes specific for methanopterin biosynthesis are absent from haloarchaea, and thus, the assignment of HVO_2348 to the methanopterin biosynthesis pathway in UniProt is invalid (accessed March 2021). 

The next common pathway step (EC 3.1.4.56) has been resolved in *M. jannaschii* (MJ0837) but is still a pathway gap in halophilic archaea. MJ0837 is very distantly related to HVO_A0533, which is a promising candidate for experimental analysis.

HVO_2628 shows 30% protein sequence identity with the enzyme catalyzing the first committed step to methanopterin biosynthesis. As detailed in Supplementary Text S1 Section S5, we consider it likely that it does not catalyze that reaction.

(c) Two enzymes that alter the oxidation level of the coenzyme-attached one-carbon compound probably function with tetrahydrofolate, even though their methanogenic homologs function with methanopterin. In contrast to their assignments in KEGG and UniProt (as of March 2021), their probable functions are thus methenyltetrahydrofolate cyclohydrolase (HVO_2573) and 5,10-methylenetetrahydrofolate reductase (HVO_1937) (see Figure 2 and Supplementary Text S1 Section S5). 

**Table 5:** Proteins with open annotation issues and their Gold Standard Protein homologs (Section 3.5). For a description of this table, see the legend to Table 1.

			Gold Standard Protein						
Section	Code	Gene	Isofunc	%seq_id	Locus Tag	UniProt	Reference	PMID	Comment
5a	HVO_0709	*pabA*	no	47%	TTHA1843	P05379	[225]	2844259	Trp biosynthesis
5a	HVO_0709 (cont.)		yes/no	39%	BSU00750	P28819	[226]	2123867	TrpG works with TrpE and with PabB
5a	HVO_0710	*pabB*	no	46%	TTHA1844	P05378	[225]	2844259	Trp biosynthesis
5a	HVO_0710 (cont.)		yes	44%	BSU00740	P28820	[227]	19275258	PabB; para-aminobenzoate biosynthesis
5a	HVO_0708	*pabC*	no	36%	AF_0933	O29329	[228]	30733943	branched-chain amino acids
5b	HVO_2348	*mptA*			self		[229]	19478918	gene deletion phenotypes
5b	HVO_2348 (cont.)		yes	41%	MJ0775	Q58185	[230]	17497938	common part of methanopterin and tetrahydrofolate biosynthesis
5b	HVO_A0533	-	unknown	27%	MJ0837	Q58247	[231]	19746965	if isofunctional would resolve a pathway gap
5b	HVO_2628	-	no	31%	AF_2089	O28190	[232]	12142414	first committed step to methanopterin biosynthesis
5b	HVO_2628 (cont.)		no	26%	MJ1427	Q58822	[233]	15262968	first committed step to methanopterin biosynthesis
5c	HVO_2573	*mch*	no	45%	MK0625	P94954	[234]	9676239	acts on a one-carbon attached to methanopterin
4c/5c	HVO_1937	*mer*	no	38%	MTH_1752	O27784	[212] [213] [214]	2298726 7649177 10891279	acts on a one-carbon compound attached to methanopterin

### 3.6. Coenzymes IV: NAD and FAD (Riboflavin)

(a) The energy source for NAD kinase may be ATP or polyphosphate. This is unresolved for the two paralogs of probable NAD kinase (HVO_2363, *nadK1* and HVO_0837, *nadK2*). These show only 25% protein sequence identity to each other (see Supplementary Text S1 Section S6). Polyphosphate was not found in exponentially growing *Hfx. volcanii* cells [235], and thus ATP is the more likely energy source.

(b) HVO_0781 is encoded in nearly all haloarchaeal genomes, according to OrthoDB, and shows very strong syntenic coupling with the adjacent gene, HVO_0782, according to SyntTax analysis. Characterized homologs to HVO_0781 cleave S-adenosyl-methionine into methionine and adenosine, a reaction that seems wasteful. If so, then this gene would not be expected to be retained in most species and neither would it maintain a strongly conserved gene clustering (see Supplementary Text S1 Section S6). HVO_0782 is an enzyme involved in NAD biosynthesis, which is encoded in most haloarchaeal and archaeal genomes. Thus, HVO_0781 is also a candidate for being involved in NAD biosynthesis. 

(c) We described the reconstruction of riboflavin biosynthesis based on a detailed bioinformatic reconstruction [236]. The enzymes and their associated GSPs are listed in Table 6. Three pathway gaps remain, with candidate genes predicted for two of these [236] (for details, see Supplementary Text S1 Section S6).

**Table 6:** Proteins with open annotation issues and their Gold Standard Protein homologs (Section 3.6). For a description of this table, see the legend to Table 1.

			Gold Standard Protein						
Section	Code	Gene	Isofunc	%seq_id	Locus Tag	UniProt	Reference	PMID	Comment
6a	HVO_2363	*nadK1*	unclear	37%	Rv1695	P9WHV7	[237]	11006082	can use ATP and PP
6a	HVO_2363 (cont.)		unclear	31%	AF_2373	O30297			ATP or PP usage unresolved
6a	HVO_0837	*nadK2*	unclear	28%	Rv1695	P9WHV7			can use ATP and PP
6a	HVO_0837 (cont.)		unclear	partial	AF_2373	O30297			ATP or PP usage unresolved
6b	HVO_0782	*nadM*	yes	53%	MJ0541	Q57961	[238] [239]	9401030 10331644	
6b	HVO_0781	*-*	unknown	42%	Sare_1364	A8M783	[240]	18720493	
6b	HVO_0781 (cont.)		unknown	35%	PH0463	O58212	[241]	18551689	
6c	HVO_0327	*ribB*	yes	43%	MJ0055	Q60364	[242]	12200440	
6c	HVO_0974	*ribH*	yes	45%	MJ0303	Q57751	[243]	12603336	
6c	HVO_1284	*arfA*		self			[244]	21999246	gene deletion leads to riboflavin auxotrophy
6c	HVO_1284 (cont.)		yes	44%	MJ0145	Q57609	[245]	12475257	
6c	HVO_1235	-	prediction				[236]	28073944	*arfB* candidate
6c	HVO_1341	*arfC*	yes	36%	MJ0671	Q58085	[246] [247]	11889103 18671734	
6c	HVO_2483	*-*	prediction	34%	MJ0699	Q58110	[236]	28073944	also predicted for MJ0699
6c	pathway gap								EC 3.1.3.104
6c	HVO_0326	*rbkR*	yes	37%	TA1064	Q9HJA6	[236]	28073944	bifunctional as gene regulator and enzyme
6c	HVO_0326 (cont.)		yes/no	32%	MJ0056	Q60365	[248]	18073108	enzyme only; lacks an N-terminal HTH domain
6c	HVO_1015	*ribL*	yes	50%	MJ1179	Q58579	[249]	20822113	

### 3.7. Biosynthesis of Membrane Lipids, Bacterioruberin and Menaquinone

**Figure 3:**
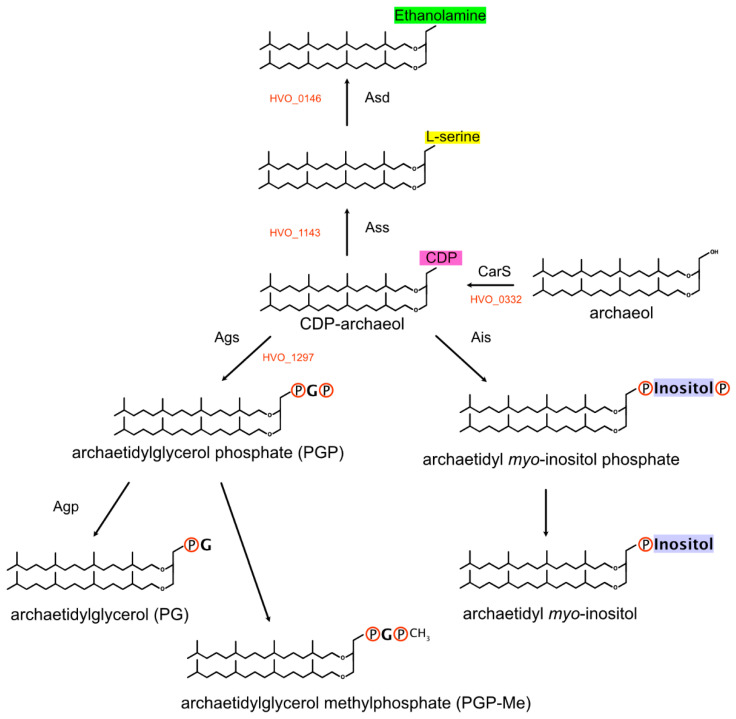
Biosynthesis of polar lipids. A key intermediate is CDP-archaeol, which is generated from archaeol (displayed as fully saturated) by CarS. Members of the InterPro:IPR000462 family then transfer the CDP-archaeol to the hydroxyl group (alcohol group) of the target molecule (backbone: serine, glycerol and myo-inositol). Subsequent modifications contribute to the diversity of polar lipids.

Archaeal membrane lipids contain ether-linked isoprenoid side chains (see [250] and the references cited therein). The isoprenoid precursor isopentenyl diphosphate is synthesized in haloarchaea by a modified version of the mevalonate pathway [251]. Isoprenoid units are then linearly condensed into the C20 compound geranylgeranyl diphosphate. The haloarchaeal core lipid, archaeol, consists of 2,3-sn-glycerol with two C20 isoprenoid side chains attached by ether linkages. In some archaea, especially alkaliphiles, C25 isoprenoids are also found (see, e.g., [252,253]). Additionally, a number of distinct headgroups are found in polar lipids (phospholipids) (reviewed in [250]) (Figure 3). Even though polar lipids are used as important taxonomic markers [254], their biosynthetic pathways are not completely resolved.

Haloarchaea typically have a red color, which is due to carotenoids, mainly the C50 carotenoid bacterioruberin [255,256,257]. For carotenoid biosynthesis, two molecules of geranylgeranyl diphosphate, a C20 compound, are linked head-to-head to generate phytoene, which is desaturated to lycopene [66,258]. The pathway from lycopene to the C50 compound bacterioruberin has been experimentally characterized [257,259].

(a) We assigned HVO_2725 (*idsA1*, paralog of NP_3696A) and HVO_0303 (*idsA2*, paralog of NP_0604A) for the linear isoprenoid condensation reactions, resulting in a C20 isoprenoid (EC 2.5.1.10 and EC 2.5.1.29, short-chain isoprenyl diphosphate synthase) (see, also, Supplementary Text S1 Section S7). Some archaea, mainly haloalkaliphiles, also contain C25 isoprenoid side chains. Geranylfarnesyl diphosphate synthase, the enzyme that generates the C25 isoprenoids, has been purified and enzymatically characterized from *Nmn. pharaonis* [260], but data required for the assignment to a specific gene have not been collected. Three paralogous genes from *Nmn. pharaonis* are candidates for this function (NP_0604A, NP_3696A and NP_4556A). Since NP_0604A and NP_3696A have orthologs in *Hfx. volcanii*, a species devoid of C25 lipids, we assigned the synthesis of C25 isoprenoids (geranylfarnesyl diphosphate synthase activity) to the third paralog, NP_4556A. UniProt assigned C25 biosynthesis activity to NP_3696A for undescribed reasons (as of April 2021), and KEGG does not make this assignment for any of the three paralogs (as of April 2021). Our assignments are supported by an analysis of the key residues that determine the length of the isoprenoid chain [261]. These authors labeled the cluster containing NP_3696A (WP011323557.1) as “C15/C20” and the cluster containing NP_4556A (WP011323984.1) as “C20->C25->C30?”.

(b) Typical polar lipids in haloarchaea (Figure 3) are phosphatidylglycerophosphate methyl ester (PGP-Me) and phosphatidylglycerol (PG) but, also, phosphatidylglycerosulfate (PGS) [261,262,263]. Other polar lipids are archaetidylserine and its decarboxylation product archaetidylethanolamine, both of which are found in rather low quantities in *Haloferax* [264]. A third group of polar lipids has a headgroup derived from myo-inositol. The biosynthetic pathway of the headgroup is only partially resolved. One CDP-archaeol 1-archaetidyltransferase that belongs to a highly conserved three-gene operon may attach either glycerol phosphate or myo-inositol phosphate. In Supplementary Text S1 Section S7, we summarize the arguments in favor of each of these candidates, but the true function can only be decided by experimental analysis.

(c) Carotenoid biosynthesis involves the head-to-head condensation of the C20 isoprenoid geranylgeranyl diphosphate to phytoene, which is desaturated to lycopene [66,258]. The *crtB* gene product (e.g., HVO_2524) catalyzes the head-to-head condensation. It is yet uncertain which gene product is responsible for the desaturation of phytoene to lycopene. The further pathway from lycopene to bacterioruberin has been experimentally characterized in *Haloarcula japonica* [257]. A three-gene cluster (*crtD*-*lyeJ*-*cruF*) codes for the three enzymes of this pathway. The synteny of this three gene cluster is strongly conserved, according to SyntTax analysis. Several genes that are certainly or possibly involved in carotenoid biosynthesis are encoded in the vicinity of this cluster (for details, see Supplementary Text S1 Section S7).

(d) Halophilic archaea contain menaquinone as a lipid-based two-electron carrier of the respiratory chain [264,265]. We described the reconstruction of the menaquinone biosynthesis pathway (Table 7), with two pathway gaps remaining open (see Supplementary Text S1 Section S7 for details).

**Table 7:** Proteins with open annotation issues and their Gold Standard Protein homologs (Section 3.7). For a description of this table, see the legend to Table 1.

			Gold Standard Protein						
Section	Code	Gene	Isofunc	%seq_id	Locus Tag	UniProt	Reference	PMID	Comment
7a	NP_0604A	*idsA2*	yes	32%	GACE_1337	A0A0A7GEY4	[266]	30062607	ortholog of HVO_0303 (66%); produces a C20 isoprenoid (same assignment for NP_0604A)
7a	NP_0604A (cont.)	*idsA2*	no	30%	APE_1764	Q9YB31 Q9UWR6	[267]	10632701	produces a C25 isoprenoid (C20 assigned to NP_0604A)
7a	NP_3996A	*idsA3*	yes	44%	GACE_1337	A0A0A7GEY4	[266]	30062607	ortholog of HVO_2725 (67%); produces a C20 isoprenoid (same assignment for NP_3996A)
7a	NP_3996A (cont.)	*idsA2*	no	36%	APE_1764	Q9YB31 Q9UWR6	[267]	10632701	produces a C25 isoprenoid (C20 assigned to NP_3996A)
7a	NP_4556A	*idsA1*	no	34%	GACE_1337	A0A0A7GEY4	[266]	30062607	no ortholog in *Hfx. volcanii*; produces a C20 isoprenoid (C25 assigned to NP_4556A)
7a	NP_4556A (cont.)	*idsA1*	yes	29%	APE_1764	Q9YB31 Q9UWR6	[267]	10632701	produces a C25 isoprenoid (same assignment for NP_4556A)
7b	HVO_0332	*carS*	yes	45%	AF_1740	O28537	[268]	25219966	
7b	HVO_1143	*assA*	yes	32%	MTH_1027	O27106	[269]	12562787	gene synonym: *pgsA3*
7b	HVO_1297	*aisA*	yes	25%	MTH_1691	O27726	[270]	19740749	gene synonym: *pgsA2*
7b	HVO_1136	*pgsA1*	-						only distant partial matches to GSPs
7b	HVO_1971	*pgsA4*	unclear	26%	MTH_1027	O27106	[269]	12562787	MTH_1027 is less distant to HVO_1143
7b	HVO_0146	*asd*	no	39%	SMc00551	Q9FDI9	[271]	18708506	equivalent function for the bacterial lipid
7b	HVO_1295	*hisC*		self			[272]	2345144	complements a His auxotrophy mutant
7b	HVO_1295 (cont.)		yes	31%	b2021	P06986	[273]	2999081	weak support, see text
7b	HVO_1296	*adk2*	unclear	34%	PAB0757	Q9UZK4	[274]	24823650	*Pyrococcus*: involved in ribosome biogenesis
7b	HVO_1296 (cont.)		unclear	32%	-	Q9Y3D8	[275]	15630091	human: adenylate kinase; HVO_1296 may be inositol kinase
7b	HVO_2496	*adk1*	yes	45%	BSU01370	P16304	[276]	31111079	*Bacillus*: adenylate kinase
7b	HVO_B0213	-	yes	43%	AF_1794	O28480	[277] [278]	11015222 22261071	*Archaeoglobus*: adenylate kinase
7b	HVO_1135	*-*	-						a SAM-dependent methyltransferase
7c	HVO_2524	*crtB*		self			[9] [279]	25488358 29038254	*crtB* mutants are colorless
7c	HVO_2524 (cont.)		yes	32%	Synpcc7942 _1984	P37269	[280]	1537409	
7c	HVO_2527	*lyeJ*		self			[259]	21840984	
7c	HVO_2527 (cont.)		yes	65%	VNG_1682C OE_3380R	Q9HPD9 B0R651	[259]	21840984	
7c	HVO_2527 (cont.)		yes	61%	C444_12922	M0L7V9	[257]	25712483	
7c	HVO_2528	*crtD*		self			[279]	29038254	a HVO_2528 mutant was white
7c	HVO_2528 (cont.)		yes	71%	C444_12917	A0A0A1GKA2	[257]	25712483	
7c	HVO_2526	*cruF*	yes	59%	C444_12927	A0A0A1GNF2	[257]	25712483	
7d	HVO_1470	*menF*	yes	38%	PA4231	Q51508	[281]	7500944	
7d	HVO_1469	*menD*	yes	37%	BSU30820	P23970	[282]	20600129	
7d	pathway gap								EC 4.2.99.20
7d	HVO_1461	*menC*	no	29%	BSU12980	O34508	[283]	11747447	Ala/Glu epimerase
7d	HVO_1461 (cont.)		yes	24%	BSU30780	O34514	[284]	10194342	o-succinylbenzoate synthase
7d	HVO_1375	*menE*	yes	36%	BSU30790	P23971	[285]	27933791	
7d	HVO_1465	*menB*	yes	66%	Rv0548c	P9WNP5	[286]	20643650	
7d	pathway gap								EC 3.1.2.28
7d	HVO_1462	*menA*	yes	37%	b3930	P32166	[287]	9573170	
7d	HVO_0309	*menG*	yes/no	44%	At3g63410	Q9LY74	[288]	14508009	*A. thaliana* enzyme also involved in tocopherol biosynthesis
7d	HVO_0309 (cont.)		yes	27%	-	O86169	[289]	9139683	

### 3.8. Issues Concerning RNA Polymerase, Protein Translation Components and Signal Peptide Degradation

(a) Haloarchaeal RNA polymerase consists of a set of canonical subunits (encoded by *rpoA1A2B1B2DEFHKLNP*). *Hbt. salinarum* and a subset of other haloarchaea contain an additional subunit called epsilon [290,291]. Purified RNA polymerase containing the epsilon subunit transcribes native templates efficiently, in contrast to the RNA polymerase devoid of this subunit [291]. The biological relevance of this subunit is enigmatic (see Supplementary Text S1 Section S8).

(b) Two distant paralogs are found for haloarchaeal ribosomal protein S10 (uS10) in nearly all haloarchaeal genomes. It is uncertain if both occur in the ribosome, whether they occur together or are mutually exclusive. The latter distribution would result in heterogeneity of the ribosomes. Alternatively, one of the paralogs may exclusively have a non-ribosomal function. 

In a subset of archaea, two distant paralogs are found for haloarchaeal ribosomal protein S14 (uS14) (ca 20% of the genomes, e.g., in *Nmn. pharaonis*). For more details, see Supplementary Text S1 Section S8.

(c) The ribosomal protein L43e (eL43) shows heterogeneity with respect to the presence of the C2–C2-type zinc finger motif. This zinc finger is found in L43e from all *Halobacteriales* and all euryarchaeal proteins outside the order *Halobacteria* but is not found in *Haloferacales* and is very rare in *Natrialbales*. Eukaryotic orthologs (e.g., from rat and yeast) contain this zinc finger, and its biological importance has been experimentally shown for the yeast protein [292] (for details, see Supplementary Text S1 Section S8).

(d) Diphthamide is a complex covalent modification of a histidine residue of translation elongation factor a-EF2. This pathway has been reconstructed (Table 8) based on distant homologs (enzymes encoded by *dph2* and *dph5*) and by a detailed bioinformatic analysis (enzyme encoded by *dph6*) [293] (for details, see Supplementary Text S1 Section S8). These uncertain function assignments await experimental confirmation.

(e) N-terminal signal sequences target proteins to the secretion machinery. Subsequent to membrane insertion or transmembrane transfer, the signal sequence is cleaved off by a signal peptidase. After cleavage, the signal peptide must be degraded to avoid clogging of the membrane. Degradation is catalyzed by signal peptide peptidase. Candidates for this activity have been predicted from two protein families [294,295] (for details, see Supplementary Text S1 Section S8).

**Table 8:** Proteins with open annotation issues and their Gold Standard Protein homologs (Section 3.8). For a description of this table, see the legend to Table 1.

			Gold Standard Protein						
Section	Code	Gene	Isofunc	%seq_id	Locus Tag	UniProt	Reference	PMID	Comment
8a	OE_1279R	*rpoeps*		self			[290] [291]	2495365 6852054	
8b	HVO_0360	*rps10a*	yes	94%	rrnAC2405	P23357	[296]	1764513	
8b	HVO_1392	*rps10b*	-						no GSP; 24% seq_id to HVO_0360 (*rps10a*)
8b	NP_4882A	*rps14a*	yes	72%	rrnAC1597.1	P26816	[297]	1832208	full-length similarity; *Haloarcula* protein was not isolated or characterized
8b	NP_4882A (cont.)		yes	57%	YDL061C	P41058	[298]	18782943	yeast YS29B; N-term 20 aa divergent
8b	NP_1768A	*rps14b*	unclear	80%	rrnAC1597.1	P26816	[297]	1832208	N-term 20 aa divergent
8c	OE_1373R	*rpl43e*	yes	69%	rrnAC1669	P60619	[299]	10937989	
8c	OE_1373R (cont.)		yes	39%	YPR043W	P0CX25	[292] [300]	10588896 11866512	
8c	HVO_0654	*rpl43e*	yes	54%	rrnAC1669	P60619	[299]	10937989	*Haloarcula*: has zinc finger; *Haloferax*; lacks zinc finger
8d	HVO_1631	*dph2*	yes	35%	PH1105	O58832	[301]	20931132	
8d	HVO_0916	*dph5*	yes	39%	PH0725	O58456	[302]	20873788	
8d	HVO_1077	*dph6*	yes	31%	YLR143W	Q12429	[303] [304]	23169644 23468660	
8e	HVO_0881	*sppA1*	yes	33%	BSU19530	O34525	[305] [306]	10455123 22472423	
8e	HVO_1987	*sppA2*	probably	23%	BSU19530	O34525	[305] [306]	10455123 22472423	
8e	HVO_1107	*-*	prediction						no GSP

### 3.9. Miscellaneous Metabolic Enzymes and Proteins with Other Functions

Here, we list a few other enzymatic or nonenzymatic functions for which candidate genes have been assigned but without experimental validation.

(a) Ketohexokinase from *Haloarcula vallismortis* has been experimentally characterized [307]. However, the activity was not assigned to a gene. Detailed bioinformatic analyses have been made [308,309] and point to a small set of orthologs represented by Hmuk_2662, the ortholog of HVO_1812 (for further details, see Supplementary Text S1 Section S9).

(b) The assignment of fructokinase activity to the *Hht. litchfieldiae* candidate gene halTADL_1913 (UniProt:A0A1H6QYL4) is based on a differential proteomic analysis [309] (see Supplementary Text S1 Section S9 for details). Very close homologs are rare in haloarchaea. For this protein family (carbohydrate kinase), it is unclear if more distant homologs (with about 50% protein sequence identity) are isofunctional.

(c) A candidate gene for glucoamylase is HVO_1711 for the reasons described in Supplementary Text S1 Section S9. The enzyme from *Halorubrum sodomense* has been characterized [310], but the activity has not yet been assigned to a gene.

(d) A strong candidate for having glucose-6-phosphate isomerase activity is *Hfx. volcanii* HVO_1967 (*pgi*), based on 36% protein sequence identity to the characterized enzyme from *M. jannaschii* (MJ1605) [311] (Table 9).

**Table 9:** Proteins with open annotation issues and their Gold Standard Protein homologs (Section 3.9). For a description of this table, see the legend to Table 1.

			Gold Standard Protein						
Section	Code	Gene	Isofunc	%seq_id	Locus Tag	UniProt	Reference	PMID	Comment
9a	HVO_1812	*-*	prediction						no GSP
9b	halTADL_1913	*-*	yes	37%	-	P26984	[312]	1809835	
9b	halTADL_1913 (cont.)	*-*	yes	31%	OCC_03567	Q7LYW8 H3ZP68	[313]	15138858	
9c	HVO_1711	*-*	probably	33%	-	P29761	[314]	1633799	P29761 matches to C-term half of HVO_1711
9c	HVO_1711 (cont.)	*-*	probably	51%	SAMN 04487937_ 2677	A0A1I6HD35	[310]	8305855	correlation between PMID:8305855 and A0A1I6HD35 likely (see text)
9d	HVO_1967	*pgi*	yes	36%	MJ1605	Q59000	[311]	14655001	
9e	OE_1665R	*kdgA*	no	31%	PA1010	Q9I4W3	[139]	21396954	GSP for *dapA* (see under 2a)
9e	OE_1665R (cont.)		probably	30%	TTX_1156.1 TTX_1156a	G4RJQ2	[315]	15869466	
9e	OE_1665R (cont.)		probably	25%	SSO3197	Q97U28	[315]	15869466	
9f	HVO_1692	*ludB*		self			[21]	30707467	
9f	HVO_1692 (cont.)		probably	35%	BSU34040	O07021	[316]	19201793	matches up to HVO_1692 pos 490 of 733
9f	HVO_1692 (cont.)		probably	35%	PST_3338	O4VPR6	[317]	25917905	matches up to HVO_1692 pos 400 of 733
9f	HVO_1693	*ludC*		self			[21]	30707467	
9f	HVO_1693 (cont.)		probably	30%	BSU34030	O32259	[316]	19201793	
9f	HVO_1693 (cont.)		probably	33%	PST_3339	O4VPR7	[317]	25917905	partial match
9f	HVO_1697	*-*	unclear	24%	PST_3340	O4VPR8	[317]	25917905	
9f	HVO_1696	*lctP*	probably	44%	PST_3336	O4VPR4	[317]	25917905	
9g	HVO_B0300	*pucL1*	yes	49%	BSU32450	O32141	[318]	20168977	*Bacillus*: bifunctional, matches to C-term
9g	HVO_B0299	*pucM*	yes	43%	BSU32460	O32142	[319]	16098976	
9g	HVO_B0301	*pucL2*	yes	43%	BSU32450	O32141	[320]	17567580	*Bacillus*: bifunctional, matches to N-term
9g	HVO_B0302	*pucH1*	no	33%	-	Q8VTT5	[321]	12148274	paper in Chinese, abstract in English; pyrimidine degradation
9g	HVO_B0302 (cont.)		yes	30%	STM0523	Q7CR08	[322]	23287969	purine degradation
9g	HVO_B0302 (cont.)		yes	29%	BSU32410	O32137	[323]	11344136	purine degradation
9g	HVO_B0306	*amaB4*	no	39%	-	Q53389	[324]	22904279	carbamoyl-AA hydrolysis
9g	HVO_B0306 (cont.)		yes	34%	At5g43600	Q8VXY9	[325] [326]	19935661 23940254	purine degradation
9g	HVO_B0308	*coxS*	no	46%	Saci_2270	Q4J6M5	[327]	10095793	GAPDH
9g	HVO_B0308 (cont.)		no	41%	-	P19915	[328]	10482497	CO-DH
9g	HVO_B0308 (cont.)		yes	39%	b2868	Q46801	[329]	10986234	xanthine DH
9g	HVO_B0309	*coxL*	yes	33%	b2866	Q46799	[329]	10986234	xanthine DH
9g	HVO_B0309 (cont.)		no	28%	-	P19913	[328]	10482497	CO-DH
9g	HVO_B0309 (cont.)		no	26%	Saci_2271	Q4J6M3	[327]	10095793	GAPDH
9g	HVO_B0310	*coxM*	no	31%	Saci_2269	Q4J6M6	[327]	10095793	GAPDH
9g	HVO_B0310 (cont.)		no	31%	-	P19914	[328]	10482497	CO-DH
9g	HVO_B0310 (cont.)		yes	25%	b2867	Q46800	[329]	10986234	xanthine DH
9g	HVO_B0303	*uraA4*	yes	38%	b3654	P0AGM9	[330]	16096267	
9h	HVO_0197	*-*	possibly	39%	lp_0105	F9UST0	[331]	27114550	LarB family protein
9h	HVO_2381	*-*	possibly	31%	lp_0106/ lp_0107	F9UST1	[331]	27114550	LarC family protein
9h	HVO_0190	*-*	possibly	34%	lp_0109	F9UST4	[331]	27114550	LarE family protein
9i	HVO_1660	*dacZ*		self			[37]	30884174	
9i	HVO_0756	*-*	prediction				[332]	32095817	
9i	HVO_0990	*-*	prediction				[332]	32095817	
9i	HVO_1690	*-*	prediction				[332]	32095817	
9j	HVO_2763	*-*		self			[333]	22350204	no function could be assigned
9j	HVO_2763 (cont.)		no	27%	HVO_0144	D4GZ88	[334]	18437358	Rnase Z
9k	HVO_2410	*dabA*	yes	33%	Hneap_0211	D0KWS7	[335]	31406332	
9k	HVO_2411	*dabB*	yes	31%	Hneap_0212	D0KWS8	[335]	31406332	

(e) A candidate gene for specifying an enzyme with 2-dehydro-3-deoxy-(phospho)gluconate aldolase activity is *Hbt. salinarum kdgA* (OE_1665R). It is rather closely related (36% protein sequence) to *Hfx. volcanii* HVO_1101 (encoded by *dapA*), which is involved in lysine biosynthesis, a biosynthetic pathway that is absent from *Hbt. salinarum*. The function assignment is based on distant homologs from *Saccharolobus (Sulfolobus) solfataricus* and *Thermoproteus tenax*, which have been characterized [315] (for details, see Supplementary Text S1 Section S9).

(f) Haloarchaea may contain an NAD-independent L-lactate dehydrogenase, LudBC (HVO_1692 and HVO_1693). The deletion of this gene pair impairs growth on rhamnose, which is catabolized to pyruvate and lactate [21]. There is a very distant relationship (for details, see Supplementary Text S1 Section S9) to the LldABC subunits of the characterized L-lactate dehydrogenase from *Pseudomonas stutzeri* A1501 [317] and to the LutABC proteins from *B. subtilis*, which have been shown to be involved in lactate utilization [316].

(g) *Hfx. volcanii* may be able to convert urate into allantoin using the gene cluster HVO_B0299-HVO_B0302. This could be part of a complete degradation pathway for purines, but this has to be considered highly speculative (see Supplementary Text S1 Section S9 and Supplementary Figure S2).

(h) *Hfx. volcanii* may contain an enzyme having a “nickel-pincer cofactor”. The biogenesis of this cofactor may be catalyzed by *larBCE* (as detailed in Supplementary Text S1 Section S9). 

(i) Cyclic di-AMP (c-di-AMP) is an important nucleotide signaling molecule in bacteria and archaea. It is generated from two molecules of ATP by diadenylate cyclase (encoded by *dacZ*) and is degraded to pApA by phosphodiesterases [336]. The level of this signaling molecule is strictly controlled [337,338], thus requiring a sophisticated interplay of cyclase and phosphodiesterase. DacZ from *Hfx. volcanii* has been characterized, and it was shown that the c-di-AMP levels must be tightly regulated [37]. The degrading enzyme, however, has not yet been identified in *Haloferax*, but candidates have been proposed [332,336,339] (see Supplementary Text S1 Section S9). 

(j) HVO_2763 is distantly related to RNase Z (HVO_0144, *rnz*). The experimental characterization of HVO_2763 [333] excluded activity as an exonuclease but did not reveal its physiological function. Upon transcriptome analysis, the downregulation of several genes was detected. Several of these were uncharacterized at the time of the experiment but have since been shown to be involved in the minor N-glycosylation pathway that was initially detected under low-salt conditions (see Supplementary Text S1 Section S9 for further details).

(k) A pair of genes (*dabAB*, HVO_2410 and HVO_2411) is predicted to function as a carbon dioxide transporter, based on the identification of such transporters in *Halothiobacillus neapolitanus* [335]. Being a member of the proton-conducting membrane transporter family, this protein may be misannotated as a subunit of the *nuo* or *mrp* complexes (see Supplementary Text S1 Section S9 for further details).

## 4. Conclusions

We described a large number of cases where the protein function cannot be correctly predicted when restricting considerations to the computational analyses without taking the biological contexts into account. An example was the switch from methanopterin to tetrahydrofolate as a C1 carrier in haloarchaea. Homologous enzymes, inherited from the common ancestor, have adapted to the new C1 carrier, rather than being replaced by non-homologous proteins. Function prediction tools may misannotate haloarchaeal proteins to work with methanopterin. Another example was the *nuo* complex and its misannotation as a type I NADH dehydrogenase. In other cases, even a distant sequence similarity may allow a valid function prediction if additional evidence (e.g., from a gene neighborhood analysis or from a detailed evaluation of the metabolic pathway gaps) is taken into account. Examples include cobalamin cluster proteins, which probably close the two residual pathway gaps, and the predicted degradation pathway for purines. In all these cases, we presented reasonable hypotheses based on the current knowledge, and in many cases, these were so well-supported as to be compelling, but to be certain, experimental data are required. With this overview, we attempted to arouse the curiosity of our colleagues, hoping that they will confirm or disprove our speculations and, thus, advance the knowledge about haloarchaeal biology. *Hfx. volcanii* is a model species for halophilic archaea, and the more complete and correctly its genome is annotated, the higher will be its value for system biology analyses (modeling) and for synthetic biology (metabolic engineering) and biotechnology.

## Figures and Tables

**Table 1:** Proteins with open annotation issues and their Gold Standard Protein homologs (Section 3.1).

			Gold Standard Protein						
Section	Code	Gene	Isofunc	%seq_id	Locus Tag	UniProt	Reference	PMID	Comment
1a	HVO_1305 HVO_1304	*porAB*	yes	67% 80%	OE2623R OE2622R	B0R4 × 6 B0R4 × 5	[87] [88] [89]	1555599 6266826 6266827	
1a	HVO_0888 HVO_0887	*korAB*	yes	77% 77%	OE1711R OE1710R	B0R3G0 B0R3F9	[88] [89]	6266826 6266827	
1a/1b	HVO_2995	*fdx*	yes	88%	OE4217R	B0R7I9	[100] [101] [86]	964365 188650 201489	role in oxidative decarboxylation
1a/1b	HVO_2995 (cont.)				self	D4GY89	[90]	22103537	role in nitrate assimilation
1c	HVO_0979 (complex)	*nuoB*	possibly	50%	tlr0705	Q8DKZ4	[102] [103] [104]	15910282 30573545 32001694	reoxidizes ferredoxin
1c	HVO_0979 (cont.)		no	48%	b2287	P0AFC7	[92] [93]	7607227 9485311	reoxidizes NADH in *E.coli*
1d	NP_3508A	*ndh1*	special	26% (N-term 140 aa)	-	Q7ZAG8			function of Q7ZAG8 was reassigned (from ndh1 to sqr) after annotation transfer
1d	NP_3508A (cont.)		possibly	30%	BpOF4_04810	A7LKG4	[105]	18359284	type II NADH dehydrogenase
1e	HVO_2620 HVO_0842 HVO_0841	*petABD*	yes	39%	SYNPCC7002_ A0842	P28056	[106]	11245788	HVO_0842 (*petB*) related to cytochrome b6
1f	HVO_2810	*sdhD*	yes	66%	NP_4268A	Q3INS7	[81] [94]	9109654 PhD_Mattar	
1g	HVO_0943	*cbaD*	yes	57%	NP_2966A	A0A1U7EWW4	[107]	9428682	
	HVO_0943 (cont.)		-	63%	OE_4073R (C-term)	B0R7A9		-	halocyanin/cbaD fusion protein, uncharacterized
1g	HVO_2150	*hcpG*	-	44%	OE_4073R (N-term)	B0R7A9		-	halocyanin/cbaD fusion protein, uncharacterized
1h	HVO_0945 (complex)	*cbaA*	yes	64%	NP_2966A	A0A1U7EWW4	[107]	9428682	
1h	HVO_0907 (complex)	*coxA1*			self		[108]	11790755	
1h	HVO_0907 (cont.)		yes	70%	VNG_0657G (OE_1979R)	P33588	[109] [110]	2542239 1659810	
1h	HVO_1645 (complex)	*coxAC2*	yes	43%	APE_0793.1	Q9YdX6	[111]	12471503	
1h	HVO_0462 HVO_0461	*cydAB*	yes	32% 24%	- -	Q09049 Q05780	[112]	1655703	
1h	HVO_0462 HVO_0461 (cont.)		yes	30% 27%	b0733 b0734	P0ABJ9 P0ABK2	[113]	6307994	
1h	NP_4296A NP_4294A	*coxA3* *coxB3*	yes	28% 33%	TTHA1135 TTHA1134	Q5SJ79 Q5SJ80	[114] [115]	2842747 7657607	
1i	HVO_2958 HVO_2959	*oadhAB1*			self	D4GY15 D4GY17	[116]	19910413	Ile indirectly assigned as substrate
1i	HVO_2958 HVO_2959 (cont.)				self		[117] [118] [119]	10832633 17571210 17906130	no substrate was identified; pyruvate and alphaKG excluded
1i	HVO_2595 HVO_2596	*oadhAB2*			self		[120] [119] [116]	12003954 17906130 19910413	no substrate was identified; pyruvate and alphaKG excluded
1i	HVO_0669 HVO_0668	*oadhAB3*			self		[119] [116]	17906130 19910413	no substrate was identified; pyruvate and alphaKG excluded
1i	HVO_2209	*oadhA4*			self				not yet analyzed experimentally
1i	HVO_2958 HVO_2959 (cont.)		yes/no	38% 52%	TA1438 TA1437	Q9HIA3 Q9HIA4	[121]	17894823	substrates are Ile, Leu, Val
1i	HVO_2595 HVO_2596 (cont.)		no	41% 41%	- -	Q57102 Q57041	[122]	1898934	substrate is acetoin
1i	HVO_2595 HVO_2596 (cont.)		unknown	40% 43%	BSU08060 BSU08070	O31404 O34591	[123]	10368162	substrate is acetoin
1i	HVO_0669 HVO_0668 (cont.)		unknown	54% 47%	BSU08060 BSU08070	O31404 O34591	[123]	10368162	substrate is acetoin
1i	HVO_0669 HVO_0668 (cont.)		unknown	49% 43%	- -	Q57102 Q57041	[122]	1898934	substrate is acetoin
1i	HVO_2209 (cont.)		unknown	38%	TA1438	Q9HIA3	[121]	17894823	substrates are Ile, Leu, Val

The column Section refers to the table listing the protein and to the section in the Results and in Supplementary Text S1. As an example, 2c covers topic (c) from the decimal-numbered Results Section 3.2. Amino Acid Biosynthesis. In Supplementary Text S1, this is covered under Section S2 subsection S2.c. The corresponding proteins are listed in Table 2. For a few proteins, two sections are indicated (e.g., 1a/1b). The column Code refers to a haloarchaeal protein by its locus tag, which is mainly from *Haloferax volcanii* (HVO) but, also, from *Halobacterium salinarum* (OE), *Natronomonas pharaonis* (NP) and *Halohasta litchfieldiae* (halTADL). When the reconstruction of a complete pathway is presented, the unassigned genes are indicated as a “pathway gap”. In one case, we indicate the absence of a haloarchaeal ortholog by a dash. In the case of a complex, we either list more than one code or we list only one subunit together with the term (complex). All subunits of these complexes are listed groupwise in Table S1. A protein may be shown in more than one row. From the 2nd row onwards, this is indicated by the term (cont.). The column Gene lists the assigned gene or a dash if no gene has been assigned. The assigned gene is only indicated in the first row of a protein. A set of four columns is used to relate a query protein to an experimentally characterized homolog, a GSP (Gold Standard Protein) (isofunc, %seq_id, Locus tag, UniProt). The column isofunc indicates if the query protein and its Gold Standard Protein homolog are isofunctional. The meanings of the terms used in this column in Table 1, Table 2, Table 3, Table 4, Table 5, Table 6, Table 7, Table 8 and Table 9 (yes, no, yes/no, probably, possibly, unclear, unknown, prediction, special and “-“) are described at the end of this legend. The column %seq_id indicates the protein sequence identity between the query protein and the homologous GSP. The column Locus tag contains the locus tag, if assigned. The column UniProt contains the UniProt accession of the GSP. GSPs are experimentally characterized as described in a publication. The column Reference links to the reference list of the manuscript. The column PMID lists the PubMed ID of the publication, if available. Otherwise, this is indicated as “not in PubMed”. Additionally, one PhD thesis is indicated (PhD_Mattar). The column Comment provides various types of additional information. The terms used in the column isofunc in Table 1, Table 2, Table 3, Table 4, Table 5, Table 6, Table 7, Table 8 and Table 9 have the following meanings: The term “yes” indicates that we consider the two proteins as isofunctional and annotate the query protein accordingly. The term “no” is used when we conclude that the proteins differ in function. Additional terms are used for more difficult cases. The term “yes/no” is used for GSPs that are multifunctional, and we assign only a subset of these functions to the query protein. The term “probably” is used when we consider it likely that the proteins are isofunctional and annotated the query protein accordingly (with the term probable added to the protein name). The term “possibly” is used when we see a good chance that the proteins are isofunctional but consider it too speculative to annotate the protein accordingly. The term “unclear” is used when we consider it likely that the same overall reaction is catalyzed but when reaction details, e.g., the energy-providing compound, are unresolved. The term “unknown” is used when it is not possible to predict the substrate of the query protein. The term “prediction” is used if a function assignment is based on bioinformatic analyses but not yet on an experimentally characterized homologous protein. The term “special” is used when multiple arguments have to be considered, with the full details provided in the corresponding section of Supplementary Text S1. Finally, a hyphen (“-“) is used when isofunctionality does not apply, e.g., when a homologous Gold Standard Protein could not be identified.

## Data Availability

Not applicable.

## Supplementary Materials

The following are available online at https://www.mdpi.com/article/10.3390/genes12070963/s1: Text S1: Detailed background information for all open annotation issues. Figure S1: Alignment of the 5’ end of *leuA2* (HVO_1510) from *Hfx. volcanii* DS2 with homologs from other species of *Haloferax*. Figure S2: The proposed purine degradation pathway. Table S1: Listing of all proteins mentioned in the text and in Supplementary Text S1. Table S2: Listing of the genomes which are manually curated and kept up to date.

## References

[1] Hartman A.L., Norais C., Badger J.H., Delmas S., Haldenby S., Madupu R., Robinson J., Khouri H., Ren Q., Lowe T.M. (2010). The complete genome sequence of *Haloferax volcanii* DS2, a model archaeon. PLoS ONE.

[2] Schulze S., Adams Z., Cerletti M., De Castro R., Ferreira-Cerca S., Fufezan C., Gimenez M.I., Hippler M., Jevtic Z., Knuppel R. (2020). The Archaeal Proteome Project advances knowledge about archaeal cell biology through comprehensive proteomics. Nat. Commun..

[3] Leigh J.A., Albers S.V., Atomi H., Allers T. (2011). Model organisms for genetics in the domain Archaea: Methanogens, halophiles, *Thermococcales* and *Sulfolobales*. FEMS Microbiol. Rev..

[4] Perez-Arnaiz P., Dattani A., Smith V., Allers T. (2020). *Haloferax volcanii*-a model archaeon for studying DNA replication and repair. Open Biol.

[5] Soppa J. (2011). Functional genomic and advanced genetic studies reveal novel insights into the metabolism, regulation, and biology of *Haloferax volcanii*. Archaea.

[6] Haque R.U., Paradisi F., Allers T. (2020). *Haloferax volcanii* for biotechnology applications: Challenges, current state and perspectives. Appl Microbiol Biotechnol.

[7] Allers T., Barak S., Liddell S., Wardell K., Mevarech M. (2010). Improved strains and plasmid vectors for conditional overexpression of His-tagged proteins in *Haloferax volcanii*. Appl Environ Microbiol.

[8] Allers T., Mevarech M. (2005). Archaeal genetics—the third way. Nat. Rev. Genet..

[9] Kiljunen S., Pajunen M.I., Dilks K., Storf S., Pohlschroder M., Savilahti H. (2014). Generation of comprehensive transposon insertion mutant library for the model archaeon, *Haloferax volcanii*, and its use for gene discovery. BMC Biol..

[10] Pfeiffer F., Broicher A., Gillich T., Klee K., Mejia J., Rampp M., Oesterhelt D. (2008). Genome information management and integrated data analysis with HaloLex. Arch. Microbiol..

[11] Pfeiffer F., Oesterhelt D. (2015). A manual curation strategy to improve genome annotation: Application to a set of haloarchael genomes. Life (Basel).

[12] Turkowyd B., Schreiber S., Wortz J., Segal E.S., Mevarech M., Duggin I.G., Marchfelder A., Endesfelder U. (2020). Establishing live-cell single-molecule localization microscopy imaging and single-particle tracking in the archaeon *Haloferax volcanii*. Front. Microbiol..

[13] Walsh J.C., Angstmann C.N., Bisson-Filho A.W., Garner E.C., Duggin I.G., Curmi P.M.G. (2019). Division plane placement in pleomorphic archaea is dynamically coupled to cell shape. Mol. Microbiol..

[14] de Silva R.T., Abdul-Halim M.F., Pittrich D.A., Brown H.J., Pohlschroder M., Duggin I.G. (2021). Improved growth and morphological plasticity of *Haloferax volcanii*. Microbiology (Reading).

[15] Duggin I.G., Aylett C.H., Walsh J.C., Michie K.A., Wang Q., Turnbull L., Dawson E.M., Harry E.J., Whitchurch C.B., Amos L.A. (2015). CetZ tubulin-like proteins control archaeal cell shape. Nature.

[16] Liao Y., Ithurbide S., Evenhuis C., Lowe J., Duggin I.G. (2021). Cell division in the archaeon *Haloferax volcanii* relies on two FtsZ proteins with distinct functions in division ring assembly and constriction. Nat. Microbiol..

[17] Brasen C., Schonheit P. (2001). Mechanisms of acetate formation and acetate activation in halophilic archaea. Arch. Microbiol..

[18] Johnsen U., Dambeck M., Zaiss H., Fuhrer T., Soppa J., Sauer U., Schonheit P. (2009). D-xylose degradation pathway in the halophilic archaeon *Haloferax volcanii*. J. Biol. Chem..

[19] Pickl A., Johnsen U., Schonheit P. (2012). Fructose degradation in the haloarchaeon *Haloferax volcanii* involves a bacterial type phosphoenolpyruvate-dependent phosphotransferase system, fructose-1-phosphate kinase, and class II fructose-1,6-bisphosphate aldolase. J. Bacteriol..

[20] Sutter J.M., Tastensen J.B., Johnsen U., Soppa J., Schonheit P. (2016). Key enzymes of the semiphosphorylative Entner-Doudoroff pathway in the haloarchaeon *Haloferax volcanii*: Characterization of glucose dehydrogenase, gluconate dehydratase and 2-keto-3-deoxy-6-phosphogluconate aldolase. J. Bacteriol..

[21] Reinhardt A., Johnsen U., Schonheit P. (2019). L-Rhamnose catabolism in archaea. Mol. Microbiol..

[22] Kuprat T., Ortjohann M., Johnsen U., Schonheit P. (2021). Glucose metabolism and acetate switch in Archaea: The enzymes in *Haloferax volcanii*. J. Bacteriol..

[23] Kuprat T., Johnsen U., Ortjohann M., Schonheit P. (2020). Acetate metabolism in Archaea: Characterization of an acetate transporter and of enzymes involved in acetate activation and gluconeogenesis in *Haloferax volcanii*. Front. Microbiol..

[24] Sutter J.M., Johnsen U., Reinhardt A., Schonheit P. (2020). Pentose degradation in archaea: *Halorhabdus* species degrade D-xylose, L-arabinose and D-ribose via bacterial-type pathways. Extremophiles.

[25] Tästensen J.B., Johnsen U., Reinhardt A., Ortjohann M., Schonheit P. (2020). D-galactose catabolism in archaea: Operation of the DeLey-Doudoroff pathway in *Haloferax volcanii*. FEMS Microbiol. Lett..

[26] Abdul-Halim M.F., Schulze S., DiLucido A., Pfeiffer F., Bisson Filho A.W., Pohlschroder M. (2020). Lipid anchoring of archaeosortase substrates and midcell growth in haloarchaea. mBio.

[27] Abdul Halim M.F., Rodriguez R., Stoltzfus J.D., Duggin I.G., Pohlschroder M. (2018). Conserved residues are critical for *Haloferax volcanii* archaeosortase catalytic activity: Implications for convergent evolution of the catalytic mechanisms of non-homologous sortases from archaea and bacteria. Mol. Microbiol..

[28] Abdul Halim M.F., Pfeiffer F., Zou J., Frisch A., Haft D., Wu S., Tolic N., Brewer H., Payne S.H., Pasa-Tolic L. (2013). *. Haloferax volcanii* archaeosortase is required for motility, mating, and C-terminal processing of the S-layer glycoprotein. Mol. Microbiol..

[29] Storf S., Pfeiffer F., Dilks K., Chen Z.Q., Imam S., Pohlschroder M. (2010). Mutational and bioinformatic analysis of haloarchaeal lipobox-containing proteins. Archaea.

[30] Schiller H., Schulze S., Mutan Z., de Vaulx C., Runcie C., Schwartz J., Rados T., Bisson Filho A.W., Pohlschroder M. (2020). *Haloferax volcanii* immersed liquid biofilms develop independently of known biofilm machineries and exhibit rapid honeycomb pattern formation. mSphere.

[31] Pohlschroder M., Esquivel R.N. (2015). Archaeal type IV pili and their involvement in biofilm formation. Front. Microbiol..

[32] Li Z., Rodriguez-Franco M., Albers S.V., Quax T.E.F. (2020). The switch complex ArlCDE connects the chemotaxis system and the archaellum. Mol. Microbiol..

[33] Collins M., Afolayan S., Igiraneza A.B., Schiller H., Krespan E., Beiting D.P., Dyall-Smith M., Pfeiffer F., Pohlschroder M. (2020). Mutations affecting HVO_1357 or HVO_2248 cause hypermotility in *Haloferax volcanii*, suggesting roles in motility regulation. Genes (Basel).

[34] Quax T.E.F., Altegoer F., Rossi F., Li Z., Rodriguez-Franco M., Kraus F., Bange G., Albers S.V. (2018). Structure and function of the archaeal response regulator CheY. Proc. Natl. Acad. Sci. USA.

[35] Nussbaum P., Ithurbide S., Walsh J.C., Patro M., Delpech F., Rodriguez-Franco M., Curmi P.M.G., Duggin I.G., Quax T.E.F., Albers S.V. (2020). An oscillating MinD protein determines the cellular positioning of the motility machinery in Archaea. Curr. Biol..

[36] Shalev Y., Turgeman-Grott I., Tamir A., Eichler J., Gophna U. (2017). Cell surface glycosylation is required for efficient mating of *Haloferax volcanii*. Front. Microbiol..

[37] Braun F., Thomalla L., van der Does C., Quax T.E.F., Allers T., Kaever V., Albers S.V. (2019). Cyclic nucleotides in archaea: Cyclic di-AMP in the archaeon *Haloferax volcanii* and its putative role. Microbiologyopen.

[38] Maier L.K., Stachler A.E., Brendel J., Stoll B., Fischer S., Haas K.A., Schwarz T.S., Alkhnbashi O.S., Sharma K., Urlaub H. (2019). The nuts and bolts of the *Haloferax* CRISPR-Cas system I-B. RNA Biol..

[39] Reuter C.J., Maupin-Furlow J.A. (2004). Analysis of proteasome-dependent proteolysis in *Haloferax volcanii* cells, using short-lived green fluorescent proteins. Appl. Environ. Microbiol..

[40] Reuter C.J., Uthandi S., Puentes J.A., Maupin-Furlow J.A. (2010). Hydrophobic carboxy-terminal residues dramatically reduce protein levels in the haloarchaeon *Haloferax volcanii*. Microbiology (Reading).

[41] Prunetti L., Reuter C.J., Hepowit N.L., Wu Y., Barrueto L., Miranda H.V., Kelly K., Maupin-Furlow J.A. (2014). Structural and biochemical properties of an extreme ’salt-loving’ proteasome activating nucleotidase from the archaeon *Haloferax volcanii*. Extremophiles.

[42] Cerletti M., Paggi R., Troetschel C., Ferrari M.C., Guevara C.R., Albaum S., Poetsch A., De Castro R. (2018). LonB protease is a novel regulator of carotenogenesis controlling degradation of phytoene synthase in *Haloferax volcanii*. J. Proteome Res..

[43] Cerletti M., Martinez M.J., Gimenez M.I., Sastre D.E., Paggi R.A., De Castro R.E. (2014). The LonB protease controls membrane lipids composition and is essential for viability in the extremophilic haloarchaeon *Haloferax volcanii*. Environ. Microbiol..

[44] Costa M.I., Cerletti M., Paggi R.A., Trotschel C., De Castro R.E., Poetsch A., Gimenez M.I. (2018). *Haloferax volcanii* proteome response to deletion of a rhomboid protease gene. J. Proteome Res..

[45] Cao S., Hepowit N., Maupin-Furlow J.A. (2015). Ubiquitin-like protein SAMP1 and JAMM/MPN+ metalloprotease HvJAMM1 constitute a system for reversible regulation of metabolic enzyme activity in Archaea. PLoS ONE.

[46] Kaminski L., Eichler J. (2014). *Haloferax volcanii* N-glycosylation: Delineating the pathway of dTDP-rhamnose biosynthesis. PLoS ONE.

[47] Tripepi M., You J., Temel S., Onder O., Brisson D., Pohlschroder M. (2012). N-Glycosylation of *Haloferax volcanii* flagellins requires known Agl proteins and Is essential for biosynthesis of stable flagella. J. Bacteriol..

[48] Gribaldo S., Schulze S., Pfeiffer F., Garcia B.A., Pohlschroder M. (2021). Comprehensive glycoproteomics shines new light on the complexity and extent of glycosylation in archaea. PLoS Biol..

[49] Shalev Y., Soucy S.M., Papke R.T., Gogarten J.P., Eichler J., Gophna U. (2018). Comparative analysis of surface layer glycoproteins and genes involved in protein glycosylation in the genus *Haloferax*. Genes (Basel).

[50] Kandiba L., Lin C.W., Aebi M., Eichler J., Guerardel Y. (2016). Structural characterization of the N-linked pentasaccharide decorating glycoproteins of the halophilic archaeon *Haloferax volcanii*. Glycobiology.

[51] Qi Q., Ito Y., Yoshimatsu K., Fujiwara T. (2016). Transcriptional regulation of dimethyl sulfoxide respiration in a haloarchaeon, *Haloferax volcanii*. Extremophiles.

[52] Rawls K.S., Yacovone S.K., Maupin-Furlow J.A. (2010). GlpR represses fructose and glucose metabolic enzymes at the level of transcription in the haloarchaeon *Haloferax volcanii*. J. Bacteriol..

[53] Hattori T., Shiba H., Ashiki K., Araki T., Nagashima Y.K., Yoshimatsu K., Fujiwara T. (2016). Anaerobic growth of haloarchaeon *Haloferax volcanii* by denitrification is controlled by the transcription regulator NarO. J. Bacteriol..

[54] Hwang S., Cordova B., Abdo M., Pfeiffer F., Maupin-Furlow J.A. (2017). ThiN as a versatile domain of transcriptional repressors and catalytic enzymes of thiamine biosynthesis. J. Bacteriol..

[55] Johnsen U., Sutter J.M., Schulz A.C., Tastensen J.B., Schonheit P. (2015). XacR—A novel transcriptional regulator of D-xylose and L-arabinose catabolism in the haloarchaeon *Haloferax volcanii*. Environ. Microbiol..

[56] Zahn S., Kubatova N., Pyper D.J., Cassidy L., Saxena K., Tholey A., Schwalbe H., Soppa J. (2021). Biological functions, genetic and biochemical characterization, and NMR structure determination of the small zinc finger protein HVO_2753 from *Haloferax volcanii*. FEBS J..

[57] Nagel C., Machulla A., Zahn S., Soppa J. (2019). Several one-domain zinc finger micro-proteins of *Haloferax volcanii* are important for stress adaptation, biofilm formation, and swarming. Genes (Basel).

[58] Kubatova N., Jonker H.R.A., Saxena K., Richter C., Vogel V., Schreiber S., Marchfelder A., Schwalbe H. (2020). Solution Structure and Dynamics of the Small Protein HVO_2922 from *Haloferax volcanii*. ChemBioChem.

[59] Straub J., Brenneis M., Jellen-Ritter A., Heyer R., Soppa J., Marchfelder A. (2009). Small RNAs in haloarchaea: Identification, differential expression and biological function. RNA Biol..

[60] Heyer R., Dorr M., Jellen-Ritter A., Spath B., Babski J., Jaschinski K., Soppa J., Marchfelder A. (2012). High throughput sequencing reveals a plethora of small RNAs including tRNA derived fragments in *Haloferax volcanii*. RNA Biol..

[61] Babski J., Maier L.K., Heyer R., Jaschinski K., Prasse D., Jager D., Randau L., Schmitz R.A., Marchfelder A., Soppa J. (2014). Small regulatory RNAs in Archaea. RNA Biol..

[62] Wyss L., Waser M., Gebetsberger J., Zywicki M., Polacek N. (2018). mRNA-specific translation regulation by a ribosome-associated ncRNA in *Haloferax volcanii*. Sci. Rep..

[63] Schnoes A.M., Brown S.D., Dodevski I., Babbitt P.C. (2009). Annotation error in public databases: Misannotation of molecular function in enzyme superfamilies. PLoS Comput. Biol..

[64] Promponas V.J., Iliopoulos I., Ouzounis C.A. (2015). Annotation inconsistencies beyond sequence similarity-based function prediction—Phylogeny and genome structure. Stand Genomic Sci.

[65] Danchin A., Ouzounis C., Tokuyasu T., Zucker J.D. (2018). No wisdom in the crowd: Genome annotation in the era of big—Current status and future prospects. Microb. Biotechnol..

[66] Falb M., Muller K., Konigsmaier L., Oberwinkler T., Horn P., von Gronau S., Gonzalez O., Pfeiffer F., Bornberg-Bauer E., Oesterhelt D. (2008). Metabolism of halophilic archaea. Extremophiles.

[67] Kanehisa M., Sato Y., Furumichi M., Morishima K., Tanabe M. (2019). New approach for understanding genome variations in KEGG. Nucleic Acids Res..

[68] UniProt C. (2021). UniProt: The universal protein knowledgebase in 2021. Nucleic Acids Res..

[69] Pfeiffer F., Losensky G., Marchfelder A., Habermann B., Dyall-Smith M. (2020). Whole-genome comparison between the type strain of *Halobacterium salinarum* (DSM 3754(T) ) and the laboratory strains R1 and NRC-1. Microbiologyopen.

[70] Tittes C., Schwarzer S., Pfeiffer F., Dyall-Smith M., Rodriguez-Franco M., Oksanen H.M., Quax T.E.F. (2021). Cellular and genomic properties of *Haloferax gibbonsii* LR2-5, the host of euryarchaeal virus HFTV1. Front. Microbiol..

[71] Hunter S., Apweiler R., Attwood T.K., Bairoch A., Bateman A., Binns D., Bork P., Das U., Daugherty L., Duquenne L. (2009). InterPro: The integrative protein signature database. Nucleic Acids Res..

[72] Kriventseva E.V., Kuznetsov D., Tegenfeldt F., Manni M., Dias R., Simao F.A., Zdobnov E.M. (2019). OrthoDB v10: Sampling the diversity of animal, plant, fungal, protist, bacterial and viral genomes for evolutionary and functional annotations of orthologs. Nucleic Acids Res..

[73] Oberto J. (2013). SyntTax: A web server linking synteny to prokaryotic taxonomy. BMC Bioinformatics.

[74] Johnson M., Zaretskaya I., Raytselis Y., Merezhuk Y., McGinnis S., Madden T.L. (2008). NCBI BLAST: A better web interface. Nucleic Acids Res..

[75] Altschul S.F., Madden T.L., Schaffer A.A., Zhang J., Zhang Z., Miller W., Lipman D.J. (1997). Gapped BLAST and PSI-BLAST: A new generation of protein database search programs. Nucleic Acids Res..

[76] Rich P.R., Marechal A. (2010). The mitochondrial respiratory chain. Essays Biochem..

[77] Guo R., Gu J., Zong S., Wu M., Yang M. (2018). Structure and mechanism of mitochondrial electron transport chain. Biomed. J..

[78] Crofts A.R., Hong S., Wilson C., Burton R., Victoria D., Harrison C., Schulten K. (2013). The mechanism of ubihydroquinone oxidation at the Qo-site of the cytochrome bc1 complex. Biochim. Biophys. Acta.

[79] Kaila V.R.I., Wikstrom M. (2021). Architecture of bacterial respiratory chains. Nat. Rev. Microbiol..

[80] Schafer G., Engelhard M., Muller V. (1999). Bioenergetics of the Archaea. Microbiol. Mol. Biol. Rev..

[81] Scharf B., Wittenberg R., Engelhard M. (1997). Electron transfer proteins from the haloalkaliphilic archaeon *Natronobacterium pharaonis*: Possible components of the respiratory chain include cytochrome bc and a terminal oxidase cytochrome ba3. Biochemistry.

[82] Sreeramulu K., Schmidt C.L., Schafer G., Anemuller S. (1998). Studies of the electron transport chain of the euryarcheon *Halobacterium salinarum*: Indications for a type II NADH dehydrogenase and a complex III analog. J. Bioenerg. Biomembr..

[83] Gradin C.H., Hederstedt L., Baltscheffsky H. (1985). Soluble succinate dehydrogenase from the halophilic archaebacterium, *Halobacterium halobium*. Arch. Biochem. Biophys..

[84] Steinert K., Wagner V., Kroth Pancic P.G., Bickel Sandkoetter S. (1997). Characterization and subunit structure of the ATP synthase of the halophilic archaeon *Haloferax volcanii* and organization of the ATP synthase genes. J. Biol. Chem..

[85] Nanba T., Mukohata Y. (1987). A membrane-bound ATPase from *Halobacterium halobium*: Purification and characterization. J. Biochem. (Tokyo).

[86] Kerscher L., Oesterhelt D. (1977). Ferredoxin is the coenzyme of α-ketoacid oxidoreductases in *Halobacterium halobium*. FEBS Lett..

[87] Plaga W., Lottspeich F., Oesterhelt D. (1992). Improved purification, crystallization and primary structure of pyruvate:ferredoxin oxidoreductase from *Halobacterium halobium*. Eur. J. Biochem..

[88] Kerscher L., Oesterhelt D. (1981). Purification and properties of two 2-oxoacid:ferredoxin oxidoreductases from *Halobacterium halobium*. Eur. J. Biochem..

[89] Kerscher L., Oesterhelt D. (1981). The catalytic mechanism of 2-oxoacid:ferredoxin oxidoreductases from *Halobacterium halobium*. One-electron transfer at two distinct steps of the catalytic cycle. Eur. J. Biochem..

[90] Zafrilla B., Martinez-Espinosa R.M., Bonete M.J., Butt J.N., Richardson D.J., Gates A.J. (2011). A haloarchaeal ferredoxin electron donor that plays an essential role in nitrate assimilation. Biochem. Soc. Trans..

[91] Falb M., Pfeiffer F., Palm P., Rodewald K., Hickmann V., Tittor J., Oesterhelt D. (2005). Living with two extremes: Conclusions from the genome sequence of *Natronomonas pharaonis*. Genome Res..

[92] Leif H., Sled V.D., Ohnishi T., Weiss H., Friedrich T. (1995). Isolation and characterization of the proton-translocating NADH: Ubiquinone oxidoreductase from *Escherichia coli*. Eur. J. Biochem..

[93] Braun M., Bungert S., Friedrich T. (1998). Characterization of the overproduced NADH dehydrogenase fragment of the NADH:ubiquinone oxidoreductase (complex I) from *Escherichia coli*. Biochemistry.

[94] Mattar S. (1996). Molekularbiologische und Biochemische Charakterisierung zweier Komplexe der Atmungskette von *Natronobacterium pharaonis*. Ph.D. Thesis.

[95] Kletzin A., Heimerl T., Flechsler J., van Niftrik L., Rachel R., Klingl A. (2015). Cytochromes c in Archaea: Distribution, maturation, cell architecture, and the special case of *Ignicoccus hospitalis*. Front. Microbiol..

[96] Sreeramulu K. (2003). Purification and partial characterization of cytochrome c552 from *Halobacterium salinarium*. Indian J. Biochem. Biophys..

[97] Mattar S., Scharf B., Kent S.B., Rodewald K., Oesterhelt D., Engelhard M. (1994). The primary structure of halocyanin, an archaeal blue copper protein, predicts a lipid anchor for membrane fixation. J. Biol. Chem..

[98] Scharf B., Engelhard M. (1993). Halocyanin, an archaebacterial blue copper protein (type I) from *Natronobacterium pharaonis*. Biochemistry.

[99] Hildebrandt P., Matysik J., Schrader B., Scharf B., Engelhard M. (1994). Raman spectroscopic study of the blue copper protein halocyanin from *Natronobacterium pharaonis*. Biochemistry.

[100] Kerscher L., Oesterhelt D. (1976). A ferredoxin from halobacteria. FEBS Lett..

[101] Kerscher L., Oesterhelt D., Cammack R., Hall D.O. (1976). A new plant-type ferredoxin from halobacteria. Eur. J. Biochem..

[102] Zhang P., Battchikova N., Paakkarinen V., Katoh H., Iwai M., Ikeuchi M., Pakrasi H.B., Ogawa T., Aro E.M. (2005). Isolation, subunit composition and interaction of the NDH-1 complexes from *Thermosynechococcus elongatus* BP-1. Biochem. J..

[103] Schuller J.M., Birrell J.A., Tanaka H., Konuma T., Wulfhorst H., Cox N., Schuller S.K., Thiemann J., Lubitz W., Setif P. (2019). Structural adaptations of photosynthetic complex I enable ferredoxin-dependent electron transfer. Science.

[104] Pan X., Cao D., Xie F., Xu F., Su X., Mi H., Zhang X., Li M. (2020). Structural basis for electron transport mechanism of complex I-like photosynthetic NAD(P)H dehydrogenase. Nat. Commun..

[105] Liu J., Krulwich T.A., Hicks D.B. (2008). Purification of two putative type II NADH dehydrogenases with different substrate specificities from alkaliphilic *Bacillus pseudofirmus* OF4. Biochim. Biophys. Acta.

[106] Lee T.-X., Metzger S.U., Cho Y.S., Whitmarsh J., Kallas T. (2001). Modification of inhibitor binding sites in the cytochrome bf complex by directed mutagenesis of cytochrome b6 in *Synechococcus* sp. PCC 7002. Biochim. Biophys. Acta.

[107] Mattar S., Engelhard M. (1997). Cytochrome ba3 from *Natronobacterium pharaonis*—An archaeal four-subunit cytochrome-c-type oxidase. Eur. J. Biochem..

[108] Tanaka M., Ogawa N., Ihara K., Sugiyama Y., Mukohata Y. (2002). Cytochrome aa(3) in *Haloferax volcanii*. J. Bacteriol..

[109] Fujiwara T., Fukumori Y., Yamanaka T. (1989). Purification and properties of *Halobacterium halobium* “cytochrome aa3” which lacks CuA and CuB. J. Biochem. (Tokyo).

[110] Denda K., Fujiwara T., Seki M., Yoshida M., Fukumori Y., Yamanaka T. (1991). Molecular cloning of the cytochrome aa3 gene from the archaeon (Archaebacterium) *Halobacterium halobium*. Biochem. Biophys. Res. Commun..

[111] Ishikawa R., Ishido Y., Tachikawa A., Kawasaki H., Matsuzawa H., Wakagi T. (2002). *Aeropyrum pernix* K1, a strictly aerobic and hyperthermophilic archaeon, has two terminal oxidases, cytochrome ba3 and cytochrome aa3. Arch. Microbiol..

[112] Moshiri F., Chawla A., Maier R.J. (1991). Cloning, characterization, and expression in *Escherichia coli* of the genes encoding the cytochrome d oxidase complex from *Azotobacter vinelandii*. J. Bacteriol..

[113] Miller M.J., Gennis R.B. (1983). The purification and characterization of the cytochrome d terminal oxidase complex of the *Escherichia coli* aerobic respiratory chain. J. Biol. Chem..

[114] Zimmermann B.H., Nitsche C.I., Fee J.A., Rusnak F., Munck E. (1988). Properties of a copper-containing cytochrome ba3: A second terminal oxidase from the extreme thermophile *Thermus thermophilus*. Proc. Natl. Acad. Sci. USA.

[115] Keightley J.A., Zimmermann B.H., Mather M.W., Springer P., Pastuszyn A., Lawrence D.M., Fee J.A. (1995). Molecular genetic and protein chemical characterization of the cytochrome ba3 from *Thermus thermophilus* HB8. J. Biol. Chem..

[116] Sisignano M., Morbitzer D., Gatgens J., Oldiges M., Soppa J. (2010). A 2-oxoacid dehydrogenase complex of *Haloferax volcanii* is essential for growth on isoleucine but not on other branched-chain amino acids. Microbiology.

[117] Jolley K.A., Maddocks D.G., Gyles S.L., Mullan Z., Tang S.L., Dyall-Smith M.L., Hough D.W., Danson M.J. (2000). 2-Oxoacid dehydrogenase multienzyme complexes in the halophilic Archaea? Gene sequences and protein structural predictions. Microbiology (Reading).

[118] Al-Mailem D.M., Hough D.W., Danson M.J. (2008). The 2-oxoacid dehydrogenase multienzyme complex of *Haloferax volcanii*. Extremophiles.

[119] van Ooyen J., Soppa J. (2007). Three 2-oxoacid dehydrogenase operons in *Haloferax volcanii*: Expression, deletion mutants and evolution. Microbiology.

[120] Wanner C., Soppa J. (2002). Functional role for a 2-oxo acid dehydrogenase in the halophilic archaeon *Haloferax volcanii*. J. Bacteriol..

[121] Heath C., Posner M.G., Aass H.C., Upadhyay A., Scott D.J., Hough D.W., Danson M.J. (2007). The 2-oxoacid dehydrogenase multi-enzyme complex of the archaeon *Thermoplasma acidophilum*—Recombinant expression, assembly and characterization. FEBS J..

[122] Oppermann F.B., Schmidt B., Steinbuchel A. (1991). Purification and characterization of acetoin:2,6-dichlorophenolindophenol oxidoreductase, dihydrolipoamide dehydrogenase, and dihydrolipoamide acetyltransferase of the *Pelobacter carbinolicus* acetoin dehydrogenase enzyme system. J. Bacteriol..

[123] Huang M., Oppermann-Sanio F.B., Steinbuchel A. (1999). Biochemical and molecular characterization of the *Bacillus subtilis* acetoin catabolic pathway. J. Bacteriol..

[124] Horie A., Tomita T., Saiki A., Kono H., Taka H., Mineki R., Fujimura T., Nishiyama C., Kuzuyama T., Nishiyama M. (2009). Discovery of proteinaceous N-modification in lysine biosynthesis of *Thermus thermophilus*. Nat. Chem. Biol..

[125] Yoshida A., Tomita T., Atomi H., Kuzuyama T., Nishiyama M. (2016). Lysine biosynthesis of *Thermococcus kodakarensis* with the capacity to function as an ornithine biosynthetic system. J. Biol. Chem..

[126] Ouchi T., Tomita T., Horie A., Yoshida A., Takahashi K., Nishida H., Lassak K., Taka H., Mineki R., Fujimura T. (2013). Lysine and arginine biosyntheses mediated by a common carrier protein in *Sulfolobus*. Nat. Chem. Biol..

[127] Hochuli M., Patzelt H., Oesterhelt D., Wuthrich K., Szyperski T. (1999). Amino acid biosynthesis in the halophilic archaeon *Haloarcula hispanica*. J. Bacteriol..

[128] Yoshida A., Tomita T., Fujimura T., Nishiyama C., Kuzuyama T., Nishiyama M. (2015). Structural insight into amino group-carrier protein-mediated lysine biosynthesis: Crystal structure of the LysZ.LysW complex from *Thermus thermophilus*. J. Biol. Chem..

[129] Shimizu T., Tomita T., Kuzuyama T., Nishiyama M. (2016). Crystal structure of the LysY.LysW complex from *Thermus thermophilus*. J. Biol. Chem..

[130] Miyazaki J., Kobashi N., Nishiyama M., Yamane H. (2001). Functional and evolutionary relationship between arginine biosynthesis and prokaryotic lysine biosynthesis through alpha-aminoadipate. J. Bacteriol..

[131] Fujita S., Cho S.H., Yoshida A., Hasebe F., Tomita T., Kuzuyama T., Nishiyama M. (2017). Crystal structure of LysK, an enzyme catalyzing the last step of lysine biosynthesis in *Thermus thermophilus*, in complex with lysine: Insight into the mechanism for recognition of the amino-group carrier protein, LysW. Biochem. Biophys. Res. Commun..

[132] Issaly I.M., Issaly A.S. (1974). Control of ornithine carbamoyltransferase activityby arginase in *Bacillus subtilis*. Eur. J. Biochem..

[133] Ruepp A., Muller H.N., Lottspeich F., Soppa J. (1995). Catabolic ornithine transcarbamylase of *Halobacterium halobium* (*salinarium*)—Purification, characterization, sequence determination, and evolution. J. Bacteriol..

[134] Shaheen N., Kobayashi K., Terazono H., Fukushige T., Horiuchi M., Saheki T. (1994). Characterization of human wild-type and mutant argininosuccinate synthetase proteins expressed in bacterial cells. Enzyme Protein.

[135] Lemke C., Yeung M., Howell P.L. (1999). Expression, purification, crystallization and preliminary X-ray analysis of *Escherichia coli* argininosuccinate synthetase. Acta Crystallogr. D Biol. Crystallogr..

[136] Cohen-Kupiec R., Kupiec M., Sandbeck K., Leigh J.A. (1999). Functional conservation between the argininosuccinate lyase of the archaeon *Methanococcus maripaludis* and the corresponding bacterial and eukaryal genes. FEMS Microbiol. Lett..

[137] Kato C., Kurihara T., Kobashi N., Yamane H., Nishiyama M. (2004). Conversion of feedback regulation in aspartate kinase by domain exchange. Biochem. Biophys. Res. Commun..

[138] Faehnle C.R., Ohren J.F., Viola R.E. (2005). A new branch in the family: Structure of aspartate-beta-semialdehyde dehydrogenase from *Methanococcus jannaschii*. J. Mol. Biol..

[139] Kaur N., Gautam A., Kumar S., Singh A., Singh N., Sharma S., Sharma R., Tewari R., Singh T.P. (2011). Biochemical studies and crystal structure determination of dihydrodipicolinate synthase from *Pseudomonas aeruginosa*. Int. J. Biol. Macromol..

[140] Reddy S.G., Sacchettini J.C., Blanchard J.S. (1995). Expression, purification, and characterization of *Escherichia coli* dihydrodipicolinate reductase. Biochemistry.

[141] Simms S.A., Voige W.H., Gilvarg C. (1984). Purification and characterization of succinyl-CoA: Tetrahydrodipicolinate N-succinyltransferase from *Escherichia coli*. J. Biol. Chem..

[142] Lin Y.K., Myhrman R., Schrag M.L., Gelb M.H. (1988). Bacterial N-succinyl-L-diaminopimelic acid desuccinylase. Purification, partial characterization, and substrate specificity. J. Biol. Chem..

[143] Wiseman J.S., Nichols J.S. (1984). Purification and properties of diaminopimelic acid epimerase from *Escherichia coli*. J. Biol. Chem..

[144] White P.J., Kelly B. (1965). Purification and properties of diaminopimelate decarboxylase from *Escherichia coli*. Biochem. J..

[145] Gulko M.K., Dyall-Smith M., Gonzalez O., Oesterhelt D. (2014). How do haloarchaea synthesize aromatic amino acids?. PLoS ONE.

[146] White R.H. (2004). L-Aspartate semialdehyde and a 6-deoxy-5-ketohexose 1-phosphate are the precursors to the aromatic amino acids in *Methanocaldococcus jannaschii*. Biochemistry.

[147] Porat I., Waters B.W., Teng Q., Whitman W.B. (2004). Two biosynthetic pathways for aromatic amino acids in the archaeon *Methanococcus maripaludis*. J. Bacteriol..

[148] Phillips R.S., Gollnick P.D. (1989). Evidence that cysteine 298 Is in the active site of tryptophan indole-lyase. J. Biol. Chem..

[149] Newton W.A., Morino Y., Snell E.E. (1965). Properties of crystalline tryptophanase. J. Biol. Chem..

[150] Oda M., Sugishita A., Furukawa K. (1988). Cloning and nucleotide sequences of histidase and regulatory genes in the *Bacillus subtilis hut* operon and positive regulation of the operon. J. Bacteriol..

[151] Hartwell L.H., Magasanik B. (1963). The molecular basis of histidase induction in *Bacillus subtilis*. J. Mol. Biol..

[152] Kaminskas E., Kimhi Y., Magasanik B. (1970). Urocanase and N-formimino-L-glutamate formiminohydrolase of *Bacillus subtilis*, two enzymes of the histidine degradation pathway. J. Biol. Chem..

[153] Yu Y., Liang Y.H., Brostromer E., Quan J.M., Panjikar S., Dong Y.H., Su X.D. (2006). A catalytic mechanism revealed by the crystal structures of the imidazolonepropionase from *Bacillus subtilis*. J. Biol. Chem..

[154] Howell D.M., Xu H., White R.H. (1999). (R)-citramalate synthase in methanogenic archaea. J. Bacteriol..

[155] Howell D.M., Harich K., Xu H.M., White R.H. (1998). Alpha-keto acid chain elongation reactions involved in the biosynthesis of coenzyme b (7-mercaptoheptanoyl threonine phosphate) in methanogenic archaea. Biochemistry.

[156] Porat I., Sieprawska-Lupa M., Teng Q., Bohanon F.J., White R.H., Whitman W.B. (2006). Biochemical and genetic characterization of an early step in a novel pathway for the biosynthesis of aromatic amino acids and p-aminobenzoic acid in the archaeon *Methanococcus maripaludis*. Mol. Microbiol..

[157] Large A., Stamme C., Lange C., Duan Z., Allers T., Soppa J., Lund P.A. (2007). Characterization of a tightly controlled promoter of the halophilic archaeon *Haloferax volcanii* and its use in the analysis of the essential *cct1* gene. Mol. Microbiol..

[158] Bali S., Lawrence A.D., Lobo S.A., Saraiva L.M., Golding B.T., Palmer D.J., Howard M.J., Ferguson S.J., Warren M.J. (2011). Molecular hijacking of siroheme for the synthesis of heme and d1 heme. Proc. Natl. Acad. Sci. USA.

[159] Siddaramappa S., Challacombe J.F., Decastro R.E., Pfeiffer F., Sastre D.E., Gimenez M.I., Paggi R.A., Detter J.C., Davenport K.W., Goodwin L.A. (2012). A comparative genomics perspective on the genetic content of the alkaliphilic haloarchaeon *Natrialba magadii* ATCC 43099^T^. BMC Genomics.

[160] Moore S.J., Lawrence A.D., Biedendieck R., Deery E., Frank S., Howard M.J., Rigby S.E., Warren M.J. (2013). Elucidation of the anaerobic pathway for the corrin component of cobalamin (vitamin B12). Proc. Natl. Acad. Sci. USA.

[161] Rodionov D.A., Vitreschak A.G., Mironov A.A., Gelfand M.S. (2003). Comparative genomics of the vitamin B12 metabolism and regulation in prokaryotes. J. Biol. Chem..

[162] Kosugi N., Araki T., Fujita J., Tanaka S., Fujiwara T. (2017). Growth phenotype analysis of heme synthetic enzymes in a halophilic archaeon, *Haloferax volcanii*. PLoS ONE.

[163] Raux E., Leech H.K., Beck R., Schubert H.L., Santander P.J., Roessner C.A., Scott A.I., Martens J.H., Jahn D., Thermes C. (2003). Identification and functional analysis of enzymes required for precorrin-2 dehydrogenation and metal ion insertion in the biosynthesis of sirohaem and cobalamin in *Bacillus megaterium*. Biochem. J..

[164] Brindley A.A., Raux E., Leech H.K., Schubert H.L., Warren M.J. (2003). A story of chelatase evolution: Identification and characterization of a small 13-15-kDa "ancestral" cobaltochelatase (CbiXS) in the archaea. J. Biol. Chem..

[165] Yin J., Xu L.X., Cherney M.M., Raux-Deery E., Bindley A.A., Savchenko A., Walker J.R., Cuff M.E., Warren M.J., James M.N. (2006). Crystal structure of the vitamin B12 biosynthetic cobaltochelatase, CbiXS, from *Archaeoglobus fulgidus*. J. Struct. Funct. Genomics.

[166] Storbeck S., Rolfes S., Raux-Deery E., Warren M.J., Jahn D., Layer G. (2010). A novel pathway for the biosynthesis of heme in Archaea: Genome-based bioinformatic predictions and experimental evidence. Archaea.

[167] Stroupe M.E., Leech H.K., Daniels D.S., Warren M.J., Getzoff E.D. (2003). CysG structure reveals tetrapyrrole-binding features and novel regulation of siroheme biosynthesis. Nat. Struct. Biol..

[168] Pennington J.M., Kemp M., McGarry L., Chen Y., Stroupe M.E. (2020). Siroheme synthase orients substrates for dehydrogenase and chelatase activities in a common active site. Nat. Commun..

[169] Schubert H.L., Rose R.S., Leech H.K., Brindley A.A., Hill C.P., Rigby S.E., Warren M.J. (2008). Structure and function of SirC from *Bacillus megaterium*: A metal-binding precorrin-2 dehydrogenase. Biochem. J..

[170] Roessner C.A., Warren M.J., Santander P.J., Atshaves B.P., Ozaki S.-i., Stolowich N.J., Iida K., Scott A.I. (1992). Expression of 9 *Salmonella typhimurium* enzymes for cobinamide synthesis. Identification of the 11-methyl and 20-methyl transferases of corrin biosynthesis. FEBS Lett..

[171] Santander P.J., Roessner C.A., Stolowich N.J., Holderman M.T., Scott A.I. (1997). How corrinoids are synthesized without oxygen: Nature’s first pathway to vitamin B12. Chem. Biol..

[172] Santander P.J., Kajiwara Y., Williams H.J., Scott A.I. (2006). Structural characterization of novel cobalt corrinoids synthesized by enzymes of the vitamin B12 anaerobic pathway. Bioorg. Med. Chem..

[173] Kajiwara Y., Santander P.J., Roessner C.A., Perez L.M., Scott A.I. (2006). Genetically engineered synthesis and structural characterization of cobalt-precorrin 5A and -5B, two new intermediates on the anaerobic pathway to vitamin B12: Definition of the roles of the CbiF and CbiG enzymes. J. Am. Chem. Soc..

[174] Fresquet V., Williams L., Raushel F.M. (2004). Mechanism of cobyrinic acid a,c-diamide synthetase from *Salmonella typhimurium* LT2. Biochemistry.

[175] Buan N.R., Rehfeld K., Escalante-Semerena J.C. (2006). Studies of the CobA-type ATP:Co(I)rrinoid adenosyltransferase enzyme of *Methanosarcina mazei* strain Go1. J. Bacteriol..

[176] Fonseca M.V., Buan N.R., Horswill A.R., Rayment I., Escalante-Semerena J.C. (2002). The ATP:Co(I)rrinoid adenosyltransferase (CobA) enzyme of *Salmonella enterica* requires the 2’-OH group of ATP for function and yields inorganic triphosphate as its reaction byproduct. J. Biol. Chem..

[177] Johnson C.L., Pechonick E., Park S.D., Havemann G.D., Leal N.A., Bobik T.A. (2001). Functional genomic, biochemical, and genetic characterization of the *Salmonella pduO* gene, an ATP:cob(I)alamin adenosyltransferase gene. J. Bacteriol..

[178] Woodson J.D., Zayas C.L., Escalante-Semerena J.C. (2003). A new pathway for salvaging the coenzyme B12 precursor cobinamide in archaea requires cobinamide-phosphate synthase (CbiB) enzyme activity. J. Bacteriol..

[179] Woodson J.D., Escalante-Semerena J.C. (2004). CbiZ, an amidohydrolase enzyme required for salvaging the coenzyme B12 precursor cobinamide in archaea. Proc. Natl. Acad. Sci. USA.

[180] Woodson J.D., Peck R.F., Krebs M.P., Escalante-Semerena J.C. (2003). The *cobY* gene of the archaeon *Halobacterium* sp. strain NRC-1 is required for de novo cobamide synthesis. J. Bacteriol..

[181] Zayas C.L., Escalante-Semerena J.C. (2007). Reassessment of the late steps of coenzyme B12 synthesis in *Salmonella enterica*: Evidence that dephosphorylation of adenosylcobalamin-5’-phosphate by the CobC phosphatase is the last step of the pathway. J. Bacteriol..

[182] O’Toole G.A., Trzebiatowski J.R., Escalante-Semerena J.C. (1994). The cobC gene of *Salmonella typhimurium* codes for a novel phosphatase involved in the assembly of the nucleotide loop of cobalamin. J. Biol. Chem..

[183] Brushaber K.R., O’Toole G.A., Escalante-Semerena J.C. (1998). CobD, a novel enzyme with L-threonine-O-3-phosphate decarboxylase activity, is responsible for the synthesis of (R)-1-amino-2-propanol O-2-phosphate, a proposed new intermediate in cobalamin biosynthesis in *Salmonella typhimurium* LT2. J. Biol. Chem..

[184] Trzebiatowski J.R., O’Toole G.A., Escalante-Semerena J.C. (1994). The cobT gene of *Salmonella typhimurium* encodes the NaMN: 5,6-dimethylbenzimidazole phosphoribosyltransferase responsible for the synthesis of N1-(5-phospho-alpha-D-ribosyl)-5,6-dimethylbenzimidazole, an intermediate in the synthesis of the nucleotide loop of cobalamin. J. Bacteriol..

[185] Debussche L., Couder M., Thibaut D., Cameron B., Crouzet J., Blanche F. (1992). Assay, purification, and characterization of cobaltochelatase, a unique complex enzyme catalyzing cobalt insertion in hydrogenobyrinic acid a,c-diamide during coenzyme B12 biosynthesis in *Pseudomonas denitrificans*. J. Bacteriol..

[186] Jensen P.E., Gibson L.C., Henningsen K.W., Hunter C.N. (1996). Expression of the *chlI*, *chlD*, and *chlH* genes from the Cyanobacterium *Synechocystis* PCC6803 in *Escherichia coli* and demonstration that the three cognate proteins are required for magnesium-protoporphyrin chelatase activity. J. Biol. Chem..

[187] Jensen P.E., Gibson L.C., Hunter C.N. (1998). Determinants of catalytic activity with the use of purified I, D and H subunits of the magnesium protoporphyrin IX chelatase from *Synechocystis* PCC6803. Biochem. J..

[188] Kuhner M., Haufschildt K., Neumann A., Storbeck S., Streif J., Layer G. (2014). The alternative route to heme in the methanogenic archaeon *Methanosarcina barkeri*. Archaea.

[189] Dailey H.A., Dailey T.A., Gerdes S., Jahn D., Jahn M., O’Brian M.R., Warren M.J. (2017). Prokaryotic heme biosynthesis: Multiple pathways to a common essential product. Microbiol. Mol. Biol. Rev..

[190] Eirich L.D., Vogels G.D., Wolfe R.S. (1979). Distribution of coenzyme F420 and properties of its hydrolytic fragments. J. Bacteriol..

[191] Jaenchen R., Schonheit P., Thauer R.K. (1984). Studies on the biosynthesis of coenzyme F420 in methanogenic bacteria. Arch. Microbiol..

[192] Lin X.L., White R.H. (1986). Occurrence of coenzyme F420 and its gamma-monoglutamyl derivative in nonmethanogenic archaebacteria. J. Bacteriol..

[193] de Wit L.E.A., Eker A.P.M. (1987). 8-Hydroxy-5-deazaflavin-dependent electron transfer in the extreme halophile *Halobacterium cutirubrum*. FEMS Microbiol. Lett..

[194] Grochowski L.L., Xu H., White R.H. (2008). Identification and characterization of the 2-phospho-L-lactate guanylyltransferase involved in coenzyme F420 biosynthesis. Biochemistry.

[195] Bashiri G., Antoney J., Jirgis E.N.M., Shah M.V., Ney B., Copp J., Stuteley S.M., Sreebhavan S., Palmer B., Middleditch M. (2019). A revised biosynthetic pathway for the cofactor F420 in prokaryotes. Nat. Commun..

[196] Braga D., Last D., Hasan M., Guo H., Leichnitz D., Uzum Z., Richter I., Schalk F., Beemelmanns C., Hertweck C. (2019). Metabolic pathway rerouting in *Paraburkholderia rhizoxinica* evolved long-overlooked derivatives of coenzyme F420. ACS Chem. Biol..

[197] Selengut J.D., Haft D.H. (2010). Unexpected abundance of coenzyme F(420)-dependent enzymes in *Mycobacterium tuberculosis* and other actinobacteria. J. Bacteriol..

[198] Graham D.E., Xu H., White R.H. (2003). Identification of the 7,8-didemethyl-8-hydroxy-5-deazariboflavin synthase required for coenzyme F(420) biosynthesis. Arch. Microbiol..

[199] Philmus B., Decamps L., Berteau O., Begley T.P. (2015). Biosynthetic versatility and coordinated action of 5’-deoxyadenosyl radicals in deazaflavin biosynthesis. J. Am. Chem. Soc..

[200] Decamps L., Philmus B., Benjdia A., White R., Begley T.P., Berteau O. (2012). Biosynthesis of F0, precursor of the F420 cofactor, requires a unique two radical-SAM domain enzyme and tyrosine as substrate. J. Am. Chem. Soc..

[201] Forouhar F., Abashidze M., Xu H., Grochowski L.L., Seetharaman J., Hussain M., Kuzin A., Chen Y., Zhou W., Xiao R. (2008). Molecular insights into the biosynthesis of the F420 coenzyme. J. Biol. Chem..

[202] Graupner M., Xu H., White R.H. (2002). Characterization of the 2-phospho-L-lactate transferase enzyme involved in coenzyme F(420) biosynthesis in *Methanococcus jannaschii*. Biochemistry.

[203] Nocek B., Evdokimova E., Proudfoot M., Kudritska M., Grochowski L.L., White R.H., Savchenko A., Yakunin A.F., Edwards A., Joachimiak A. (2007). Structure of an amide bond forming F(420):gamma-glutamyl ligase from *Archaeoglobus fulgidus*—A member of a new family of non-ribosomal peptide synthases. J. Mol. Biol..

[204] Li H., Graupner M., Xu H., White R.H. (2003). CofE catalyzes the addition of two glutamates to F420-0 in F420 coenzyme biosynthesis in *Methanococcus jannaschii*. Biochemistry.

[205] Kunow J., Schwoërer B., Stetter K.O., Thauer R.K. (1993). A F420-dependent NADP reductase in the extremely thermophilic sulfate-reducing *Archaeoglobus fulgidus*. Arch. Microbiol..

[206] Purwantini E., Mukhopadhyay B. (2013). Rv0132c of *Mycobacterium tuberculosis* encodes a coenzyme F420-dependent hydroxymycolic acid dehydrogenase. PLoS ONE.

[207] Klein A.R., Berk H., Purwantini E., Daniels L., Thauer R.K. (1996). Si-face stereospecificity at C5 of coenzyme F420 for F420-dependent glucose-6-phosphate dehydrogenase from *Mycobacterium smegmatis* and F420-dependent alcohol dehydrogenase from *Methanoculleus thermophilicus*. Eur. J. Biochem..

[208] Aufhammer S.W., Warkentin E., Berk H., Shima S., Thauer R.K., Ermler U. (2004). Coenzyme binding in F420-dependent secondary alcohol dehydrogenase, a member of the bacterial luciferase family. Structure.

[209] Haase P., Deppenmeier U., Blaut M., Gottschalk G. (1992). Purification and characterization of F420H2-dehydrogenase from *Methanolobus tindarius*. Eur. J. Biochem..

[210] Westenberg D.J., Braune A., Ruppert C., Muller V., Herzberg C., Gottschalk G., Blaut M. (1999). The F420H2-dehydrogenase from *Methanolobus tindarius*: Cloning of the *ffd* operon and expression of the genes in *Escherichia coli*. FEMS Microbiol. Lett..

[211] Johnson E.F., Mukhopadhyay B. (2005). A new type of sulfite reductase, a novel coenzyme F420-dependent enzyme, from the methanarchaeon *Methanocaldococcus jannaschii*. J. Biol. Chem..

[212] te Brömmelstroet B.W., Hensgens C.M., Keltjens J.T., van der Drift C., Vogels G.D. (1990). Purification and properties of 5,10-methylenetetrahydromethanopterin reductase, a coenzyme F420-dependent enzyme, from *Methanobacterium thermoautotrophicum* strain delta H. J. Biol. Chem..

[213] Vaupel M., Thauer R.K. (1995). Coenzyme F420-dependent N5,N10-methylenetetrahydromethanopterin reductase (Mer) from *Methanobacterium thermoautotrophicum* strain Marburg. Cloning, sequencing, transcriptional analysis, and functional expression in *Escherichia coli* of the *mer* gene. Eur. J. Biochem..

[214] Shima S., Warkentin E., Grabarse W., Sordel M., Wicke M., Thauer R.K., Ermler U. (2000). Structure of coenzyme F(420) dependent methylenetetrahydromethanopterin reductase from two methanogenic archaea. J. Mol. Biol..

[215] Takao M., Kobayashi T., Oikawa A., Yasui A. (1989). Tandem arrangement of photolyase and superoxide dismutase genes in *Halobacterium halobium*. J. Bacteriol..

[216] McCready S., Marcello L. (2003). Repair of UV damage in *Halobacterium salinarum*. Biochem. Soc. Trans..

[217] Brudler R., Hitomi K., Daiyasu H., Toh H., Kucho K.-i., Ishiura M., Kanehisa M., Roberts V.A., Todo T., Tainer J.A. (2003). Identification of a new cryptochrome class. Mol. Cell.

[218] Kleine T., Lockhart P., Batschauer A. (2003). An *Arabidopsis* protein closely related to *Synechocystis* cryptochrome is targeted to organelles. Plant J..

[219] Selby C.P., Sancar A. (2006). A cryptochrome/photolyase class of enzymes with single-stranded DNA-specific photolyase activity. Proc. Natl. Acad. Sci. USA.

[220] Zhang F., Scheerer P., Oberpichler I., Lamparter T., Krauss N. (2013). Crystal structure of a prokaryotic (6-4) photolyase with an Fe-S cluster and a 6,7-dimethyl-8-ribityllumazine antenna chromophore. Proc. Natl. Acad. Sci. USA.

[221] White R.H. (1988). Analysis and characterization of the folates in the nonmethanogenic archaebacteria. J. Bacteriol..

[222] Maden B.E. (2000). Tetrahydrofolate and tetrahydromethanopterin compared: Functionally distinct carriers in C1 metabolism. Biochem. J..

[223] de Crecy-Lagard V. (2014). Variations in metabolic pathways create challenges for automated metabolic reconstructions: Examples from the tetrahydrofolate synthesis pathway. Comput. Struct. Biotechnol. J..

[224] Pfeiffer F., Schuster S.C., Broicher A., Falb M., Palm P., Rodewald K., Ruepp A., Soppa J., Tittor J., Oesterhelt D. (2008). Evolution in the laboratory: The genome of *Halobacterium salinarum* strain R1 compared to that of strain NRC-1. Genomics.

[225] Sato S., Nakada Y., Kanaya S., Tanaka T. (1988). Molecular cloning and nucleotide sequence of *Thermus thermophilus* HB8 *trpE* and *trpG*. Biochim. Biophys. Acta.

[226] Slock J., Stahly D.P., Han C.Y., Six E.W., Crawford I.P. (1990). An apparent *Bacillus subtilis* folic acid biosynthetic operon containing *pab*, an amphibolic *trpG* gene, a third gene required for synthesis of para-aminobenzoic acid, and the dihydropteroate synthase gene. J. Bacteriol..

[227] Schadt H.S., Schadt S., Oldach F., Sussmuth R.D. (2009). 2-Amino-2-deoxyisochorismate is a key intermediate in *Bacillus subtilis* p-aminobenzoic acid biosynthesis. J. Am. Chem. Soc..

[228] Isupov M.N., Boyko K.M., Sutter J.M., James P., Sayer C., Schmidt M., Schonheit P., Nikolaeva A.Y., Stekhanova T.N., Mardanov A.V. (2019). Thermostable branched-chain amino acid transaminases from the archaea *Geoglobus acetivorans* and *Archaeoglobus fulgidus*: Biochemical and structural characterization. Front. Bioeng. Biotechnol..

[229] El Yacoubi B., Phillips G., Blaby I.K., Haas C.E., Cruz Y., Greenberg J., de Crecy-Lagard V. (2009). A Gateway platform for functional genomics in *Haloferax volcanii*: Deletion of three tRNA modification genes. Archaea.

[230] Grochowski L.L., Xu H., Leung K., White R.H. (2007). Characterization of an Fe(2+)-dependent archaeal-specific GTP cyclohydrolase, MptA, from *Methanocaldococcus jannaschii*. Biochemistry.

[231] Mashhadi Z., Xu H., White R.H. (2009). An Fe^2+^-dependent cyclic phosphodiesterase catalyzes the hydrolysis of 7,8-dihydro-D-neopterin 2’,3’-cyclic phosphate in methanopterin biosynthesis. Biochemistry.

[232] Scott J.W., Rasche M.E. (2002). Purification, overproduction, and partial characterization of beta-RFAP synthase, a key enzyme in the methanopterin biosynthesis pathway. J. Bacteriol..

[233] Dumitru R.V., Ragsdale S.W. (2004). Mechanism of 4-(beta-D-ribofuranosyl)aminobenzene 5’-phosphate synthase, a key enzyme in the methanopterin biosynthetic pathway. J. Biol. Chem..

[234] Vaupel M., Vorholt J.A., Thauer R.K. (1998). Overproduction and one-step purification of the N5,N10-methenyltetrahydromethanopterin cyclohydrolase (Mch) from the hyperthermophilic *Methanopyrus kandleri*. Extremophiles.

[235] Zerulla K., Chimileski S., Nather D., Gophna U., Papke R.T., Soppa J. (2014). DNA as a phosphate storage polymer and the alternative advantages of polyploidy for growth or survival. PLoS ONE.

[236] Rodionova I.A., Vetting M.W., Li X., Almo S.C., Osterman A.L., Rodionov D.A. (2017). A novel bifunctional transcriptional regulator of riboflavin metabolism in Archaea. Nucleic Acids Res..

[237] Kawai S., Mori S., Mukai T., Suzuki S., Yamada T., Hashimoto W., Murata K. (2000). Inorganic Polyphosphate/ATP-NAD kinase of *Micrococcus flavus* and *Mycobacterium tuberculosis* H37Rv. Biochem. Biophys. Res. Commun..

[238] Raffaelli N., Pisani F.M., Lorenzi T., Emanuelli M., Amici A., Ruggieri S., Magni G. (1997). Characterization of nicotinamide mononucleotide adenylyltransferase from thermophilic archaea. J. Bacteriol..

[239] Raffaelli N., Emanuelli M., Pisani F.M., Amici A., Lorenzi T., Ruggieri S., Magni G. (1999). Identification of the archaeal NMN adenylyltransferase gene. Mol. Cell. Biochem..

[240] Eustaquio A.S., Harle J., Noel J.P., Moore B.S. (2008). S-Adenosyl-L-methionine hydrolase (adenosine-forming), a conserved bacterial and archaeal protein related to SAM-dependent halogenases. ChemBioChem.

[241] Deng H., Botting C.H., Hamilton J.T., Russell R.J., O’Hagan D. (2008). S-adenosyl-L-methionine:hydroxide adenosyltransferase: A SAM enzyme. Angew. Chem. Int. Ed. Engl..

[242] Fischer M., Romisch W., Schiffmann S., Kelly M., Oschkinat H., Steinbacher S., Huber R., Eisenreich W., Richter G., Bacher A. (2002). Biosynthesis of riboflavin in archaea studies on the mechanism of 3,4-dihydroxy-2-butanone-4-phosphate synthase of *Methanococcus jannaschii*. J. Biol. Chem..

[243] Haase I., Mortl S., Kohler P., Bacher A., Fischer M. (2003). Biosynthesis of riboflavin in archaea. 6,7-dimethyl-8-ribityllumazine synthase of *Methanococcus jannaschii*. Eur. J. Biochem..

[244] Phillips G., Grochowski L.L., Bonnett S., Xu H., Bailly M., Blaby-Haas C., El Yacoubi B., Iwata-Reuyl D., White R.H., de Crecy-Lagard V. (2012). Functional promiscuity of the COG0720 family. ACS Chem. Biol..

[245] Graham D.E., Xu H., White R.H. (2002). A member of a new class of GTP cyclohydrolases produces formylaminopyrimidine nucleotide monophosphates. Biochemistry.

[246] Graupner M., Xu H., White R.H. (2002). The pyrimidine nucleotide reductase step in riboflavin and F(420) biosynthesis in archaea proceeds by the eukaryotic route to riboflavin. J. Bacteriol..

[247] Romisch-Margl W., Eisenreich W., Haase I., Bacher A., Fischer M. (2008). 2,5-diamino-6-ribitylamino-4(3H)-pyrimidinone 5’-phosphate synthases of fungi and archaea. FEBS J..

[248] Ammelburg M., Hartmann M.D., Djuranovic S., Alva V., Koretke K.K., Martin J., Sauer G., Truffault V., Zeth K., Lupas A.N. (2007). A CTP-dependent archaeal riboflavin kinase forms a bridge in the evolution of cradle-loop barrels. Structure.

[249] Mashhadi Z., Xu H., Grochowski L.L., White R.H. (2010). Archaeal RibL: A new FAD synthetase that is air sensitive. Biochemistry.

[250] Caforio A., Driessen A.J.M. (2017). Archaeal phospholipids: Structural properties and biosynthesis. Biochim. Biophys. Acta Mol. Cell Biol. Lipids.

[251] Vannice J.C., Skaff D.A., Keightley A., Addo J.K., Wyckoff G.J., Miziorko H.M. (2014). Identification in *Haloferax volcanii* of phosphomevalonate decarboxylase and isopentenyl phosphate kinase as catalysts of the terminal enzyme reactions in an archaeal alternate mevalonate pathway. J. Bacteriol..

[252] De Rosa M., Gambacorta A. (1988). The lipids of archaebacteria. Prog. Lipid Res..

[253] Dawson K.S., Freeman K.H., Macalady J.L. (2012). Molecular characterization of core lipids from halophilic archaea grown under different salinity conditions. Org. Geochem..

[254] Oren A., Ventosa A., Grant W.D. (1997). Proposed minimal standards for description of new taxa in the order *Halobacteriales*. Int. J. Syst. Bacteriol..

[255] Oren A. (2002). Molecular ecology of extremely halophilic Archaea and Bacteria. FEMS Microbiol. Ecol..

[256] Kushwaha S.C., Kramer J.K.G., Kates M. (1975). Isolation and characterization of C50-carotenoid pigments and other polar isoprenoids from *Halobacterium cutirubrum*. Biochim. Biophys. Acta.

[257] Yang Y., Yatsunami R., Ando A., Miyoko N., Fukui T., Takaichi S., Nakamura S. (2015). Complete biosynthetic pathway of the C50 carotenoid bacterioruberin from lycopene in the extremely halophilic archaeon *Haloarcula japonica*. J. Bacteriol..

[258] Giani M., Miralles-Robledillo J.M., Peiro G., Pire C., Martinez-Espinosa R.M. (2020). Deciphering pathways for carotenogenesis in haloarchaea. Molecules.

[259] Dummer A.M., Bonsall J.C., Cihla J.B., Lawry S.M., Johnson G.C., Peck R.F. (2011). Bacterioopsin-mediated regulation of bacterioruberin biosynthesis in *Halobacterium salinarum*. J. Bacteriol..

[260] Tachibana A. (1994). A novel prenyltransferase, farnesylgeranyl diphosphate synthase, from the haloalkaliphilic archaeon, *Natronobacterium pharaonis*. FEBS Lett..

[261] Bale N.J., Sorokin D.Y., Hopmans E.C., Koenen M., Rijpstra W.I.C., Villanueva L., Wienk H., Sinninghe Damste J.S. (2019). New insights into the polar lipid composition of extremely halo(alkali)philic euryarchaea from hypersaline lakes. Front. Microbiol..

[262] Kates M. (1993). Biology of halophilic bacteria, Part II. Membrane lipids of extreme halophiles: Biosynthesis, function and evolutionary significance. Experientia.

[263] Kates M., Moldoveanu N., Stewart L.C. (1993). On the revised structure of the major phospholipid of *Halobacterium salinarium*. Biochim. Biophys. Acta.

[264] Kellermann M.Y., Yoshinaga M.Y., Valentine R.C., Wormer L., Valentine D.L. (2016). Important roles for membrane lipids in haloarchaeal bioenergetics. Biochim. Biophys. Acta.

[265] Elling F.J., Becker K.W., Konneke M., Schroder J.M., Kellermann M.Y., Thomm M., Hinrichs K.U. (2016). Respiratory quinones in Archaea: Phylogenetic distribution and application as biomarkers in the marine environment. Environ. Microbiol..

[266] Petrova T.E., Boyko K.M., Nikolaeva A.Y., Stekhanova T.N., Gruzdev E.V., Mardanov A.V., Stroilov V.S., Littlechild J.A., Popov V.O., Bezsudnova E.Y. (2018). Structural characterization of geranylgeranyl pyrophosphate synthase GACE1337 from the hyperthermophilic archaeon *Geoglobus acetivorans*. Extremophiles.

[267] Tachibana A., Yano Y., Otani S., Nomura N., Sako Y., Taniguchi M. (2000). Novel prenyltransferase gene encoding farnesylgeranyl diphosphate synthase from a hyperthermophilic archaeon, *Aeropyrum pernix*—Molecular evolution with alteration in product specificity. Eur. J. Biochem..

[268] Jain S., Caforio A., Fodran P., Lolkema J.S., Minnaard A.J., Driessen A.J.M. (2014). Identification of CDP-archaeol synthase, a missing link of ether lipid biosynthesis in Archaea. Chem. Biol..

[269] Morii H., Koga Y. (2003). CDP-2,3-Di-O-geranylgeranyl-sn-glycerol:L-serine O-archaetidyltransferase (archaetidylserine synthase) in the methanogenic archaeon *Methanothermobacter thermautotrophicus*. J. Bacteriol..

[270] Morii H., Kiyonari S., Ishino Y., Koga Y. (2009). A novel biosynthetic pathway of archaetidyl-myo-inositol via archaetidyl-myo-inositol phosphate from CDP-archaeol and D-glucose 6-phosphate in methanoarchaeon *Methanothermobacter thermautotrophicus* cells. J. Biol. Chem..

[271] Vences-Guzman M.A., Geiger O., Sohlenkamp C. (2008). *Sinorhizobium meliloti* mutants deficient in phosphatidylserine decarboxylase accumulate phosphatidylserine and are strongly affected during symbiosis with alfalfa. J. Bacteriol..

[272] Conover R.K., Doolittle W.F. (1990). Characterization of a gene involved in histidine biosynthesis in *Halobacterium* (*Haloferax*) *volcanii*: Isolation and rapid mapping by transformation of an auxotroph with cosmid DNA. J. Bacteriol..

[273] Grisolia V., Carlomagno M.S., Nappo A.G., Bruni C.B. (1985). Cloning, structure, and expression of the *Escherichia coli* K-12 *hisC* gene. J. Bacteriol..

[274] Loc’h J., Blaud M., Rety S., Lebaron S., Deschamps P., Bareille J., Jombart J., Robert-Paganin J., Delbos L., Chardon F. (2014). RNA mimicry by the fap7 adenylate kinase in ribosome biogenesis. PLoS Biol..

[275] Ren H., Wang L., Bennett M., Liang Y., Zheng X., Lu F., Li L., Nan J., Luo M., Eriksson S. (2005). The crystal structure of human adenylate kinase 6: An adenylate kinase localized to the cell nucleus. Proc. Natl. Acad. Sci. USA.

[276] Moon S., Kim J., Koo J., Bae E. (2019). Structural and mutational analyses of psychrophilic and mesophilic adenylate kinases highlight the role of hydrophobic interactions in protein thermal stability. Struct. Dyn..

[277] Chen L., Zhou C., Yang H., Roberts M.F. (2000). Inositol-1-phosphate synthase from *Archaeoglobus fulgidus* is a class II aldolase. Biochemistry.

[278] Neelon K., Roberts M.F., Stec B. (2011). Crystal structure of a trapped catalytic intermediate suggests that forced atomic proximity drives the catalysis of mIPS. Biophys. J..

[279] Maurer S., Ludt K., Soppa J. (2018). Characterization of copy number control of two *Haloferax volcanii* replication origins using deletion mutants and haloarchaeal artificial chromosomes. J. Bacteriol..

[280] Chamovitz D., Misawa N., Sandmann G., Hirschberg J. (1992). Molecular cloning and expression in *Escherichia coli* of a cyanobacterial gene coding for phytoene synthase, a carotenoid biosynthesis enzyme. FEBS Lett..

[281] Serino L., Reimmann C., Baur H., Beyeler M., Visca P., Haas D. (1995). Structural genes for salicylate biosynthesis from chorismate in *Pseudomonas aeruginosa*. Mol. Gen. Genet..

[282] Dawson A., Chen M., Fyfe P.K., Guo Z., Hunter W.N. (2010). Structure and reactivity of *Bacillus subtilis* MenD catalyzing the first committed step in menaquinone biosynthesis. J. Mol. Biol..

[283] Schmidt D.M., Hubbard B.K., Gerlt J.A. (2001). Evolution of enzymatic activities in the enolase superfamily: Functional assignment of unknown proteins in *Bacillus subtilis* and *Escherichia coli* as L-Ala-D/L-Glu epimerases. Biochemistry.

[284] Palmer D.R., Garrett J.B., Sharma V., Meganathan R., Babbitt P.C., Gerlt J.A. (1999). Unexpected divergence of enzyme function and sequence: "N-acylamino acid racemase" is o-succinylbenzoate synthase. Biochemistry.

[285] Chen Y., Jiang Y., Guo Z. (2016). Mechanistic insights from the crystal structure of *Bacillus subtilis* o-succinylbenzoyl-CoA synthetase complexed with the adenylate intermediate. Biochemistry.

[286] Jiang M., Chen M., Guo Z.F., Guo Z. (2010). A bicarbonate cofactor modulates 1,4-dihydroxy-2-naphthoyl-coenzyme a synthase in menaquinone biosynthesis of *Escherichia coli*. J. Biol. Chem..

[287] Suvarna K., Stevenson D., Meganathan R., Hudspeth M.E. (1998). Menaquinone (vitamin K2) biosynthesis: Localization and characterization of the menA gene from *Escherichia coli*. J. Bacteriol..

[288] Cheng Z., Sattler S., Maeda H., Sakuragi Y., Bryant D.A., DellaPenna D. (2003). Highly divergent methyltransferases catalyze a conserved reaction in tocopherol and plastoquinone synthesis in cyanobacteria and photosynthetic eukaryotes. Plant Cell.

[289] Koike-Takeshita A., Koyama T., Ogura K. (1997). Identification of a novel gene cluster participating in menaquinone (vitamin K2) biosynthesis. Cloning and sequence determination of the 2-heptaprenyl-1,4-naphthoquinone methyltransferase gene of *Bacillus stearothermophilus*. J. Biol. Chem..

[290] Leffers H., Gropp F., Lottspeich F., Zillig W., Garrett R.A. (1989). Sequence, organization, transcription and evolution of RNA polymerase subunit genes from the archaebacterial extreme halophiles *Halobacterium halobium* and *Halococcus morrhuae*. J. Mol. Biol..

[291] Madon J., Zillig W. (1983). A form of the DNA-dependent RNA polymerase of *Halobacterium halobium*, containing an additional component, is able to transcribe native DNA. Eur. J. Biochem..

[292] Rivlin A.A., Chan Y.L., Wool I.G. (1999). The contribution of a zinc finger motif to the function of yeast ribosomal protein YL37a. J. Mol. Biol..

[293] de Crecy-Lagard V., Forouhar F., Brochier-Armanet C., Tong L., Hunt J.F. (2012). Comparative genomic analysis of the DUF71/COG2102 family predicts roles in diphthamide biosynthesis and B12 salvage. Biol. Direct.

[294] Ng S.Y., Chaban B., VanDyke D.J., Jarrell K.F. (2007). Archaeal signal peptidases. Microbiology (Reading).

[295] Raut P., Glass J.B., Lieberman R.L. (2021). Archaeal roots of intramembrane aspartyl protease siblings signal peptide peptidase and presenilin. Proteins.

[296] Arndt E., Scholzen T., Kromer W., Hatakeyama T., Kimura M. (1991). Primary structures of ribosomal proteins from the archaebacterium *Halobacterium marismortui* and the eubacterium *Bacillus stearothermophilus*. Biochimie.

[297] Scholzen T., Arndt E. (1991). Organization and nucleotide sequence of ten ribosomal protein genes from the region equivalent to the spectinomycin operon in the archaebacterium *Halobacterium marismortui*. Mol. Genet. Genomics.

[298] Otaka E., Higo K., Itoh T. (1984). Yeast ribosomal proteins. VIII. Isolation of two proteins and sequence characterization of twenty-four proteins from cytoplasmic ribosomes. Mol. Gen. Genet..

[299] Ban N., Nissen P., Hansen J., Moore P.B., Steitz T.A. (2000). The complete atomic structure of the large ribosomal subunit at 2.4 A resolution. Science.

[300] Dresios J., Chan Y.L., Wool I.G. (2002). The role of the zinc finger motif and of the residues at the amino terminus in the function of yeast ribosomal protein YL37a. J. Mol. Biol..

[301] Zhu X., Dzikovski B., Su X., Torelli A.T., Zhang Y., Ealick S.E., Freed J.H., Lin H. (2011). Mechanistic understanding of *Pyrococcus horikoshii* Dph2, a [4Fe-4S] enzyme required for diphthamide biosynthesis. Mol. Biosyst..

[302] Zhu X., Kim J., Su X., Lin H. (2010). Reconstitution of diphthine synthase activity in vitro. Biochemistry.

[303] Su X., Lin Z., Chen W., Jiang H., Zhang S., Lin H. (2012). Chemogenomic approach identified yeast YLR143W as diphthamide synthetase. Proc. Natl. Acad. Sci. USA.

[304] Uthman S., Bar C., Scheidt V., Liu S., ten Have S., Giorgini F., Stark M.J., Schaffrath R. (2013). The amidation step of diphthamide biosynthesis in yeast requires DPH6, a gene identified through mining the DPH1-DPH5 interaction network. PLoS Genet..

[305] Bolhuis A., Matzen A., Hyyrylainen H.L., Kontinen V.P., Meima R., Chapuis J., Venema G., Bron S., Freudl R., van Dijl J.M. (1999). Signal peptide peptidase- and ClpP-like proteins of *Bacillus subtilis* required for efficient translocation and processing of secretory proteins. J. Biol. Chem..

[306] Nam S.E., Kim A.C., Paetzel M. (2012). Crystal structure of *Bacillus subtilis* signal peptide peptidase A. J. Mol. Biol..

[307] Rangaswamy V., Altekar W. (1994). Ketohexokinase (ATP:D-fructose 1-phosphotransferase) from a halophilic archaebacterium, *Haloarcula vallismortis*: Purification and properties. J. Bacteriol..

[308] Anderson I., Scheuner C., Goker M., Mavromatis K., Hooper S.D., Porat I., Klenk H.P., Ivanova N., Kyrpides N. (2011). Novel insights into the diversity of catabolic metabolism from ten haloarchaeal genomes. PLoS ONE.

[309] Williams T.J., Allen M.A., Liao Y., Raftery M.J., Cavicchioli R. (2019). Sucrose metabolism in haloarchaea: Reassessment using genomics, proteomics, and metagenomics. Appl. Environ. Microbiol..

[310] Chaga G., Porath J., Illeni T. (1993). Isolation and purification of amyloglucosidase from *Halobacterium sodomense*. Biomed. Chromatogr..

[311] Rudolph B., Hansen T., Schonheit P. (2004). Glucose-6-phosphate isomerase from the hyperthermophilic archaeon *Methanococcus jannaschii*: Characterization of the first archaeal member of the phosphoglucose isomerase superfamily. Arch. Microbiol..

[312] Aulkemeyer P., Ebner R., Heilenmann G., Jahreis K., Schmid K., Wrieden S., Lengeler J.W. (1991). Molecular analysis of two fructokinases involved in sucrose metabolism of enteric bacteria. Mol. Microbiol..

[313] Qu Q., Lee S.J., Boos W. (2004). Molecular and biochemical characterization of a fructose-6-phosphate-forming and ATP-dependent fructokinase of the hyperthermophilic archaeon *Thermococcus litoralis*. Extremophiles.

[314] Ohnishi H., Kitamura H., Minowa T., Sakai H., Ohta T. (1992). Molecular cloning of a glucoamylase gene from a thermophilic *Clostridium* and kinetics of the cloned enzyme. Eur. J. Biochem..

[315] Ahmed H., Ettema T.J., Tjaden B., Geerling A.C., van der Oost J., Siebers B. (2005). The semi-phosphorylative Entner-Doudoroff pathway in hyperthermophilic archaea: A re-evaluation. Biochem. J..

[316] Chai Y., Kolter R., Losick R. (2009). A widely conserved gene cluster required for lactate utilization in *Bacillus subtilis* and its involvement in biofilm formation. J. Bacteriol..

[317] Gao C., Wang Y., Zhang Y., Lv M., Dou P., Xu P., Ma C. (2015). NAD-independent L-lactate dehydrogenase required for L-lactate utilization in *Pseudomonas stutzeri* A1501. J. Bacteriol..

[318] Pfrimer P., de Moraes L.M., Galdino A.S., Salles L.P., Reis V.C., De Marco J.L., Prates M.V., Bloch C., Torres F.A. (2010). Cloning, purification, and partial characterization of *Bacillus subtilis* urate oxidase expressed in *Escherichia coli*. J. Biomed. Biotechnol..

[319] Lee Y., Lee D.H., Kho C.W., Lee A.Y., Jang M., Cho S., Lee C.H., Lee J.S., Myung P.K., Park B.C. (2005). Transthyretin-related proteins function to facilitate the hydrolysis of 5-hydroxyisourate, the end product of the uricase reaction. FEBS Lett..

[320] Kim K., Park J., Rhee S. (2007). Structural and functional basis for (S)-allantoin formation in the ureide pathway. J. Biol. Chem..

[321] Xu Z., Jiang W.H., Jiao R.S., Yang Y.L. (2002). [Cloning, sequencing and high expression in *Escherichia coli* of D-hydantoinase gene from *Burkholderia pickettii*]. Sheng Wu Gong Cheng Xue Bao.

[322] Ho Y.Y., Huang Y.H., Huang C.Y. (2013). Chemical rescue of the post-translationally carboxylated lysine mutant of allantoinase and dihydroorotase by metal ions and short-chain carboxylic acids. Amino Acids.

[323] Schultz A.C., Nygaard P., Saxild H.H. (2001). Functional analysis of 14 genes that constitute the purine catabolic pathway in *Bacillus subtilis* and evidence for a novel regulon controlled by the PucR transcription activator. J. Bacteriol..

[324] Martinez-Rodriguez S., Garcia-Pino A., Las Heras-Vazquez F.J., Clemente-Jimenez J.M., Rodriguez-Vico F., Garcia-Ruiz J.M., Loris R., Gavira J.A. (2012). Mutational and structural analysis of L-N-carbamoylase reveals new insights into a peptidase M20/M25/M40 family member. J. Bacteriol..

[325] Werner A.K., Romeis T., Witte C.P. (2010). Ureide catabolism in *Arabidopsis thaliana* and *Escherichia coli*. Nat. Chem. Biol..

[326] Werner A.K., Medina-Escobar N., Zulawski M., Sparkes I.A., Cao F.Q., Witte C.P. (2013). The ureide-degrading reactions of purine ring catabolism employ three amidohydrolases and one aminohydrolase in *Arabidopsis*, soybean, and rice. Plant Physiol..

[327] Kardinahl S., Schmidt C.L., Hansen T., Anemuller S., Petersen A., Schafer G. (1999). The strict molybdate-dependence of glucose-degradation by the thermoacidophile *Sulfolobus acidocaldarius* reveals the first crenarchaeotic molybdenum containing enzyme—An aldehyde oxidoreductase. Eur. J. Biochem..

[328] Kang B.S., Kim Y.M. (1999). Cloning and molecular characterization of the genes for carbon monoxide dehydrogenase and localization of molybdopterin, flavin adenine dinucleotide, and iron-sulfur centers in the enzyme of *Hydrogenophaga pseudoflava*. J. Bacteriol..

[329] Xi H., Schneider B.L., Reitzer L. (2000). Purine catabolism in *Escherichia coli* and function of xanthine dehydrogenase in purine salvage. J. Bacteriol..

[330] Karatza P., Frillingos S. (2005). Cloning and functional characterization of two bacterial members of the NAT/NCS2 family in *Escherichia coli*. Mol. Membr. Biol..

[331] Desguin B., Soumillion P., Hols P., Hausinger R.P. (2016). Nickel-pincer cofactor biosynthesis involves LarB-catalyzed pyridinium carboxylation and LarE-dependent sacrificial sulfur insertion. Proc. Natl. Acad. Sci. USA.

[332] He J., Yin W., Galperin M.Y., Chou S.H. (2020). Cyclic di-AMP, a second messenger of primary importance: Tertiary structures and binding mechanisms. Nucleic Acids Res..

[333] Fischer S., John von Freyend S., Sabag-Daigle A., Daniels C.J., Allers T., Marchfelder A. (2012). Assigning a function to a conserved archaeal metallo-beta-lactamase from *Haloferax volcanii*. Extremophiles.

[334] Spath B., Schubert S., Lieberoth A., Settele F., Schutz S., Fischer S., Marchfelder A. (2008). Two archaeal tRNase Z enzymes: Similar but different. Arch. Microbiol..

[335] Desmarais J.J., Flamholz A.I., Blikstad C., Dugan E.J., Laughlin T.G., Oltrogge L.M., Chen A.W., Wetmore K., Diamond S., Wang J.Y. (2019). DABs are inorganic carbon pumps found throughout prokaryotic phyla. Nat. Microbiol..

[336] Corrigan R.M., Grundling A. (2013). Cyclic di-AMP: Another second messenger enters the fray. Nat. Rev. Microbiol..

[337] Gundlach J., Mehne F.M., Herzberg C., Kampf J., Valerius O., Kaever V., Stulke J. (2015). An essential poison: Synthesis and degradation of cyclic di-AMP in *Bacillus subtilis*. J. Bacteriol..

[338] Commichau F.M., Heidemann J.L., Ficner R., Stulke J. (2019). Making and breaking of an essential poison: The cyclases and phosphodiesterases that produce and degrade the essential second messenger cyclic di-AMP in bacteria. J. Bacteriol..

[339] Yin W., Cai X., Ma H., Zhu L., Zhang Y., Chou S.H., Galperin M.Y., He J. (2020). A decade of research on the second messenger c-di-AMP. FEMS Microbiol. Rev..

