# Supplementary material for: Open Issues for Protein Function Assignment in Haloferax volcanii and Other Halophilic Archaea
Source: Genes (Basel). 2021 Jun 24;12(7):963. doi: 10.3390/genes12070963 (PMC8305020; doi:10.3390/genes12070963)
Supplement: Supplementary file 1 [file genes-12-00963-s001.zip › asSubm_openIssues_Supp_210622.pdf]

SUPPLEMENTARY TABLES

Section	Code (*Hfx. volcanii*)	Gene	Code (other species or paralog)	Gene	%seq_id	Comment
1a	**HVO_1305**	*porA*	OE2623R	*porA*	67%	
1a	**HVO_1304**	*porB*	OE2622R	*porB*	80%	
1a	**HVO_0888**	*korA*	OE1711R	*korA*	77%	
1a	**HVO_0887**	*korB*	OE1710R	*korB*	77%	
1a/1b	HVO_2995	*fdx*	OE4217R	*fdx*	88%	
1c	**HVO_0978**	*nuoA*				
1c	**HVO_0979**	*nuoB*				
1c	**HVO_0980**	*nuoCD1*				gene fusion
1c	**HVO_0981**	*nuoH*				
1c	**HVO_0982**	*nuoI*				
1c	**HVO_0983**	*nuoJ1*				N-term of NuoJ
1c	**HVO_0984**	*nuoJ2*				C-term of NuoJ
1c	**HVO_0985**	*nuoK*				
1c	**HVO_0986**	*nuoL*				
1c	**HVO_0987**	*nuoM*				
1c	**HVO_0988**	*nuoN*				
1c	HVO_0968	*nuoCD2*	HVO_0980	*nuoCD1*	75%	
1d	HVO_1578	*ndh1*	NP_3508A	*ndh*		
1d	HVO_1413	*ndh2*	HVO_1578	*ndh1*	75%	
1e	**HVO_2620**	*petA*	OE_1876R	*petA*	54%	
1e	**HVO_0842**	*petB*	OE_1874R	*petB*	81%	
1e	**HVO_0841**	*petD*	OE_1872R	*petD*	77%	
1f	**HVO_2808**	*sdhA*	NP_4264A	*sdhA*	76%	
1f	**HVO_2809**	*sdhB*	NP_4266A	*sdhB*	76%	
1f	**HVO_2810**	*sdhD*	NP_4268A	*sdhD*	66%	
1f	**HVO_2811**	*sdhC*	NP_4270A	*sdhC*	81%	
1g/1h	HVO_0943	*cbaD*	NP_2966A	*cbaD*	57%	
1g/1h	HVO_0943	*cbaD*	OE_4073R	*hcpB*	63%	matches to C-term
1g	HVO_2150	*hcpG*	OE_4073R	*hcpB*	44%	matches to N-term
1h	**HVO_0945**	*cbaA*	NP_2962A	*cbaA*	64%	
1h	**HVO_0944**	*cbaB*	NP_2964A	*cbaB*	60%	
1g/1h	**HVO_0943**	*cbaD*	NP_2966A	*cbaD*	57%	
1h	**HVO_0942**	*cbaE*	NP_2968A	*cbaE*	45%	
1h	HVO_0945	*cbaA*	OE_4070R	*cbaA*	65%	
1h	HVO_0944	*cbaB*	OE_4071R	*cbaB*	56%	
1g/1h	HVO_0943	*cbaD*	OE_4073R	*hcpB*	63%	gene function in *Hbt. salinarum*; matches to C-term
1h	HVO_0942	*cbaE*	-	-		no ortholog in *Hbt. salinarum*
1h	**HVO_0907**	*coxA1*	NP_2456A	*coxA1*	68%	
1h	**HVO_1014**	*coxB1*	NP_2448A	*coxB1*	50%	
1h	**HVO_1138**	*coxC1*	NP_2450A	*coxC1*	59%	
1h	HVO_0907	*coxA1*	OE_1979R	*coxA1*	70%	
1h	HVO_1014	*coxB1*	OE_1988R	*coxB1*	43%	
1h	HVO_1138	*coxC1*	OE_1984F	*coxC1*	65%	
1h	**HVO_1645**	*coxAC2*				
1h	**HVO_1646**	*coxB2*				
1h	**HVO_1644**	*coxD*				

1h	HVO_1645	*coxAC2*	HVO_0907	*coxA1*	39%	matches to N-term
1h	HVO_1645	*coxA23*	HVO_1138	*coxC1*	28%	matches to C-term
1h	**HVO_0462**	*cydA*	OE_6185F OE_7065F	*cydA*	56%	encoded on a plasmid pHS1/pHS2 duplication
1h	**HVO_0461**	*cydB*	OE_6186F OE_7066F	*cydB*	45%	encoded on a plasmid pHS1/pHS2 duplication
1h	**NP_4296A**	*coxA3*	Hhub_2087		60%	
1h	**NP_4294A**	*coxB3*	Hhub_2086		57%	
1h	NP_4296A	*coxA3*	NP_2962A	*cbaA*	28%	
1h	NP_4294A	*coxB3*	NP_2964A	*cbaB*	-	33% seq_id in the C-term ca 80 aa
1i	**HVO_2958**	*oadhA1*				component E1 alpha subunit
1i	**HVO_2959**	*oadhB1*				component E1 beta subunit
1i	**HVO_2960**	*dsa1*				component E2
1i	**HVO_2961**	*lpdA*				component E3
1i	HVO_2209	*oadhA4*				component E1 alpha subunit; no gene cluster
1i	**HVO_0669**	*oadhA3*				component E1 alpha subunit
1i	**HVO_0668**	*oadhB3*				component E1 beta subunit
1i	**HVO_0667**	-				homolog to NAD kinase
1i	**HVO_0666**	*dsa2*				component E2
1i	**HVO_2595**	*oadhA2*				component E1 alpha subunit
1i	**HVO_2596**	*oadhB2*				component E1 beta subunit
1i	**HVO_2597**	*oadhL*				a short protein consisting of a lipoyl domain
1i	HVO_2958	*oadhA1*	HVO_2209	*oadhA4*	59%	
1i	HVO_2958	*oadhA1*	HVO_0669	*oadhA3*	33%	
1i	HVO_2958	*oadhA1*	HVO_2595	*oadhA2*	33%	
1i	HVO_0669	*oadhA3*	HVO_2595	*oadhA2*	41%	
1i	HVO_0669	*oadhA3*	HVO_2209	*oadhA4*	30%	
1i	HVO_2595	*oadhA2*	HVO_2209	*oadhA4*	32%	
1i	HVO_2959	*oadhB1*	HVO_0668	*oadhB3*	43%	
1i	HVO_2959	*oadhB1*	HVO_2596	*oadhB2*	41%	
1i	HVO_0668	*oadhB3*	HVO_2596	*oadhB2*	42%	
1i	HVO_2960	*dsa1*	HVO_0666	*dsa2*	31%	
1i	HVO_2597	*oadhL*	HVO_2960	*dsa1*	-	38% seq_id in N-term ca 80 aa of *dsa1*
1i	HVO_2597	*oadhL*	HVO_0666	*dsa2*	-	35% seq_id in N-term ca 80 aa of *dsa2*
1i	HVO_2597	*oadhL*	HVO_2486	*pccA*		35% seq_id in N-term ca 80 aa of *pccA*
2a	HVO_0041	*argF*				Arg biosynthesis
2a	HVO_0042	*argE*				Arg biosynthesis
2a	HVO_0043	*argD*				Arg biosynthesis
2a	HVO_0044	*argB*				Arg biosynthesis
2a	HVO_0045	*argC*				Arg biosynthesis
2a	HVO_0046	*argX*				Arg biosynthesis
2a	HVO_0047	*argW*				Arg biosynthesis
2a	HVO_0048	*argH*				Arg biosynthesis
2a	HVO_0049	*argG*				Arg biosynthesis
2a	HVO_0008	*lysC*				Lys biosynthesis
2a	HVO_1096	*dapE*				Lys biosynthesis
2a	HVO_1097	*dapF*				Lys biosynthesis
2a	HVO_1098	*lysA*				Lys biosynthesis

2a	HVO_1099	*dapD*				Lys biosynthesis
2a	HVO_1100	*dapB*				Lys biosynthesis
2a	HVO_1101	*dapA*				Lys biosynthesis
2a	HVO_2487	*asd*				Lys biosynthesis
2a	HVO_A0634	-	HVO_1096	*dapE*	25%	KEGG annotates HVO_A0634 also as DapE
2b	HVO_0790	*fba2*				
2b	HVO_0792	*aroB*				
2b	HVO_0602	*aroD1*	HVO_0603	*aroD2*	45%	
2b	HVO_0603	*aroD2*				
2c	HVO_0009	*tnaA*				
2d	HVO_A0559	*hutH*				
2d	HVO_A0562	*hutU*				
2d	HVO_A0560	*hutI*				
2d	HVO_A0561	*hutG*				
2e	HVO_0431	-				
2e/7b	HVO_1295	*hisC*				
2e	HVO_1153	-				
2e	HVO_0644	*leuA1*	HVO_1510	*leuA2*	39%	
2e	HVO_0644	*leuA1*	HVO_A0489	-	37%	
2e/2f	HVO_1510	*leuA2*	HfgLR_07660	*leuA2*	99%	Met-1/Met-1
2e/2f	HVO_1510	*leuA2*	HFX_1573	*leuA2*	95%	Leu-27/Leu-1; no start codon
2e/2f	HVO_1510	*leuA2*	HQ2700A	*leuA1*	76%	Cys-30/Cys-10
2e/2f	HVO_1510	*leuA2*	Hqrw_3051	*leuA1*	76%	Cys-30/Cys-10
2e/2f	HVO_1510	*leuA2*	rrnAC0329	*leuA2*	78%	Cys-30/Cys-8
2e/2f	HVO_1510	*leuA2*	HAH_1067	*leuA1*	77%	Cys-30/Cys-8
2e/2f	HVO_1510	*leuA2*	Nmag_0906	*leuA1*	73%	Cys-30/Cys-18
2e/2f	HVO_1510	*leuA2*	HBSAL_05970	*leuA*	68%	Phe-46/Phe-6; no start codon
2e/2f	HVO_1510	*leuA2*	Hhub_3057	*leuA1*	70%	Glu-45/Glu-7
2e/2f	HVO_1510	*leuA2*	NP_2206A	*leuA1*	79%	Arg-36/Arg-4; no start codon
2e/2f	HVO_1510	*leuA2*	Nmlp_3164	*leuA1*	76%	Arg-36/Arg-4; no start codon
2e/2f	HVO_1510	*leuA2*	halTADL_0359	*leuA1*	78%	Cys-30/Cys-1; no start codon
2e	HVO_A0489	-				
2e	HVO_1153	-				
2e	HVO_1154	-				
2e	HVO_1155	-				
3a	HVO_B0054	*cbiX1*	NP1108A	*cbiX1*	71%	
3a	HVO_1128	*cbiX2*	NP1588A	*cbiX2*	79%	
3a	HVO_1128	*cbiX2*	NP0734A	*cbiX3*	26%	
3a	HVO_1128	*cbiX2*	Nmag_3212	*cbiX*	79%	no de novo cobalamin biosynthesis genes in *Nab. magadii*
3a	-		NP_0734A	*cbiX3*		no ortholog in *Hfx.volcanii*
3a	HVO_2312	*sirC*				
3b	HVO_B0061	*cbiL*				
3b	HVO_B0057	*cbiH2*	HVO_B0058	*cbiH1*	38%	
3b	HVO_B0058	*cbiH1*				
3b	HVO_B0060	*cbiF*				
3b	HVO_B0059	*cbiG*				
3b	HVO_B0062	*cbiT*				
3b	HVO_B0048	*cbiE*				
3b	HVO_B0049	*cbiC*				
3b	HVO_A0487	*cbiA*				
3b	HVO_B0059	*cbiG*	G3A49_06890		98%	

3b	HVO_B0058	*cbiH1*	G3A49_06895		94%	
3b	HVO_B0057	*cbiH2*	G3A49_06900		99%	
3b	HVO_B0056		G3A49_06905		97%	
3b	HVO_B0055		G3A49_06910		90%	
3b	HVO_B0054	*cbiX1*	G3A49_06915		98%	
3b	HVO_B0053		G3A49_06920		99%	
3b	HVO_B0052		G3A49_06925		99%	
3b	HVO_B0051	*chlID*	G3A49_06930		97%	
3b	HVO_B0050	*cobN*	G3A49_06935		98%	
3b	HVO_B0049	*cbiC*	G3A49_06940		99%	
3b	HVO_B0048	*cbiE*	G3A49_06945		94%	
3c	HVO_A0488	*cobA*				
3c	HVO_2395	*pduO*				
3c	HVO_A0553	*cbiP*				
3c	HVO_0587	*cbiB*				
3c	HVO_0592	*cbiZ*				
3c	HVO_0589	*cobY*				
3c	HVO_0588	*cobS*				
3c	HVO_0586	-	VNG_1577C<br>OE_3249F	-	56%	predicted to be isofunctional to *cobC*
3c	HVO_0591	*cobD1*				
3c	HVO_0593	*cobD2*	HVO_0591	*cobD1*	51%	
3c	HVO_0590	*cobT*	VNG_1572C<br>OE_3242F	*cobT*	57%	
3c	-		halTADL_3045	*cobT*		
3d	HVO_B0051	*cobN*				
3d	HVO_B0050	*chlID*				
3e	HVO_1121	*ahbC*				
3e	HVO_2144	*ahbD*				
3e	HVO_2227	*ahbA*				
3e	HVO_2313	*ahbB*	HVO_2227	*ahbA*	39%	
4a	HVO_2198	*cofH*				
4a	HVO_2201	*cofG*				
4a	HVO_2202	*cofC*				
4a	HVO_2479	*cofD*				
4a	HVO_1936	*cofE*				
4b	HVO_0433	*npdG*				
4b	HVO_B0113	-				
4b	HVO_B0342	-				
4b	HVO_A0572	-	NP_1902A	-	44%	
4b	NP_1902A	-	NP_4374A	-	33%	
4b	-		NP_4006A	-		
4b	HVO_A0605	-				
4c/5c	HVO_1937	*mer*				
4d	HVO_2911	*phr2*	HVO_2843	*phr1*	31%	
4d	HVO_2843	*phr1*				
4d	HVO_1234	*phr3*				
5a	HVO_0709	*pabA*	HVO_2453	*trpG*	44%	
5a	HVO_0710	*pabB*	HVO_2454	*trpE*	37%	
5a	HVO_0708	*pabC*	HVO_0329	*ilvE*	32%	
5b	HVO_2348	*mptA*				
5b	HVO_A0533	-				
5b	HVO_2628	-				
5c	HVO_2573	*mch*				

4c/5c	HVO_1937	*mer*				
6a	HVO_2363	*nadK1*	HVO_0837	*nadK2*	25%	
6a	HVO_0837	*nadK2*				
6b	HVO_0782	*nadM*				
6b	HVO_0781	-				
6c	HVO_0326	*rbkR*				
6c	HVO_0327	*ribB*				
6c	HVO_0974	*ribH*				
6c	HVO_1015	*ribL*				
6c	HVO_1284	*arfA*				
6c	HVO_1235	-				*arfB* candidate
6c	HVO_1341	*arfC*				
6c	HVO_2483	-				*RIB2* candidate
6c	HVO_0326	*rbkR*				
6c	HVO_1015	*ribL*				
7a	HVO_0303	*idsA2*	NP_0604A	*idsA2*	66%	
7a	HVO_2725	*idsA1*	NP_3996A	*idsA3*	67%	
7a	-		NP_4556A	*idsA1*		
7b	HVO_0332	*carS*				
7b	HVO_1136	*pgsA1*				
7b	HVO_1143	*assA*	VNG_0784G<br>OE_2155R	*assA*	56%	
7b	HVO_1297	*aisA*	VNG_1030G<br>OE_2503R	*agsA*	64%	
7b	HVO_1971	*pgsA4*				
7b	HVO_1297	*aisA*	NP2144A	*aisA*	66%	
7b	HVO_1135	-				
7b	HVO_0146	*asd*	VNG_2255C<br>OE_4164F	*asd*	61%	
2e/7b	HVO_1295	*hisC*				
7b	HVO_1296	*adk2*				
7b	HVO_2496	*adk1*				
7b	HVO_B0213	-				
7c	HVO_2524	*crtB*	NP_4770A	*crtB*	57%	
7c	HVO_2527	*lyeJ*	NP_4766A	*lyeJ*	61%	
7c	HVO_2528	*crtD*	NP_4764A	*crtD*	72%	
7c	HVO_2528	*crtD*	NP_0204A	*crtI2*	57%	
7c	HVO_2526	*cruF*	NP_4768A	*cruF*	60%	
7c	HVO_0817	-	NP_1630A	-	54%	
7c	HVO_0817	-	HFX_0786	-	88%	
7c	HVO_2340	-	NP_4520A	-	69%	
7c	NP_4764A	*crtD*	NP_0204A	*crtI2*	60%	
7c	NP_0204A	*crtI2*	NP_4520A	-	26%	
7c	NP_0204A	*crtI2*	NP_1630A	-	26%	
7c	HVO_2529	-	NP_4762A	-	60%	
7d	HVO_1470	*menF*				
7d	HVO_1469	*menD*				
7d	HVO_1461	*menC*				
7d	HVO_1375	*menE*				
7d	HVO_1465	*menB*				
7d	HVO_1462	*menA*				
7d	HVO_0309	*menG*				
7d	HVO_1463	*trxB4*				in *men* gene cluster
7d	HVO_1464	-				in *men* gene cluster

7d	HVO_1466	-				in *men* gene cluster
7d	HVO_1467	-				in *men* gene cluster
7d	HVO_1468	-				in *men* gene cluster
8a	-		OE_1279R	*rpoeps*		
8b	HVO_0360	*rps10a*				
8b	HVO_1392	*rps10b*	HVO_0360	*rps10a*	24%	
8b	HVO_0361	*tef1a1*				
8b	HVO_1391	-				
8b	HVO_2550	*rps14*	NP_4882A	*rps14a*	75%	full-length
8b	HVO_2550	*rps14*	NP_1768A	*rps14b*	80%	partial
8c	HVO_0654	*rpl43e*	OE_1373R	*rpl43e*	53%	zinc finger only in OE_1373R
8d	HVO_1631	*dph2*				
8d	HVO_0916	*dph5*				
8d	HVO_1077	*dph6*				
8e	HVO_0881	*sppA1*				
8e	HVO_1987	*sppA2*	HVO_0881	*sppA1*	28%	
8e	HVO_1107	-				
8e	HVO_0002	*sec11b*				
8e	HVO_2603	*sec11a*	HVO_0002	*sec11b*	54%	
9a	HVO_1812	-	Hmuk_2662	-	50%	
9b	-		halTADL_1913	-		
9c	HVO_1711	-				
9d	HVO_1967	*pgi*				
2a/9e	HVO_1101	*dapA*	OE_1665R	*kdgA*	36%	
2a/9e	HVO_1101	*dapA*	Hhub_1591	*dapA*	69%	
2a/9e	HVO_1101	*dapA*	Hhub_1547	*kdgA*	32%	
9e	OE_1665R	*kdgA*	Hhub_1547	*kdgA*	77%	
9e	OE_1665R	*kdgA*	Hhub_1591	*dapA*	37%	
9e	HVO_1488	*gnaD*	OE_1664R	*gnaD*	88%	
9e	HVO_1083	*gdh*	OE_1669F	*gdh*	62%	
9f	HVO_1692	*ludB*	NP_1724A	*ludB*	60%	
9f	HVO_1693	*ludC*	NP_1724A	*ludC*	44%	
9f	HVO_1697	-				
9f	HVO_1696	*lctP*				
9g	HVO_B0300	*pucL1*				
9g	HVO_B0299	*pucM*				
9g	HVO_B0301	*pucL2*				
9g	HVO_B0302	*pucH1*	HVO_A0303	*pucH2*	61%	
9g	HVO_B0306	*amaB4*	HVO_2128	*amaB*	55%	
9g	HVO_B0306	*amaB4*	HVO_A0341	*amaB*	54%	
9g	HVO_B0308	*coxS*				
9g	HVO_B0309	*coxL*				
9g	HVO_B0310	*coxM*				
9g	HVO_B0303	*uraA4*				
9h	HVO_0197	-				
9h	HVO_2381	-				
9h	HVO_0197	-				
9i	HVO_1660	*dacZ*				
9i	HVO_0990	-	HVO_1690	-	39%	full-length similarity
9i	HVO_0990	-	HVO_0756	-	38%	similarity starts at pos 140/55
9i	HVO_1690	-	HVO_0756	-	32%	similarity starts at pos 129/32
9i	HVO_0756	-				
9j	HVO_2763	-	HVO_0144	*rnz*	27%	

9k	HVO_2410	*dabA*				
9k	HVO_2411	*dabB*	HVO_0986	*nuoL*	29%	up to pos 328 of 494; up to pos 372 of 678
9k	HVO_2411	*dabB*	HVO_0987	*nuoM*	24%	up to pos 325 of 494; up to pos 362 of 509
9k	HVO_2411	*dabB*	HVO_0988	*nuoN*	25%	up to pos 322 of 494; up to pos 368 of 506
9k	HVO_2411	*dabB*	HVO_1069	*mrpA*	26%	up to pos 421 of 494; up to pos 454 of 801
9k	HVO_2411	*dabB*	HVO_1066	*mrpD1*	24%	up to pos 359 of 494; up to pos 409 of 565

Table S1: Listing of all proteins mentioned in the text and in Suppl. Text S1. This table lists all haloarchaeal proteins which are mentioned in the manuscript. Proteins may be listed more than once. (1) The column Section refers to the section in the Results and in Suppl. Text S1. As an example, 2c covers topic (c) from the decimal-numbered Results subsection 3.2 Amino Acid Biosynthesis. In Suppl. Text S1, this is covered under Section S2 subsection S2.c. Table 2 lists proteins from this section. For a few proteins, two sections are indicated (e.g. 1a/1b). (2) The two columns headed Code refer to proteins by their locus tag. Nearly all codes in the first Code column are from *Haloferax volcanii* (HVO). The second Code column "(other species or paralog)" contains information only when a paralog or a haloarchaeal homolog is indicated. The correlation between genome and locus tag prefix is available from Table S2. Protein complexes are represented in this table. Subunits that belong to the same complex are listed in consecutive rows which are highlighted in grey. If adjacent rows contain subunits of distinct protein complexes, the highlighting toggles between light grey and dark grey. If subunits of complexes are listed more than once, then the complex is only indicated at first listing. (3) The two columns headed Gene list the assigned gene for the corresponding locus_tag in the left adjacent Code column, or a dash if no gene has been assigned. (4) The column headed %seq_id indicates the protein sequence identity in case of paralogs or haloarchaeal homologs. (5) The column headed Comment provides various types of additional information.

Species	Strain	Collection	Accessions	Locus tag prefix	Reference	PMID	Contribution
Halobacterium salinarum	R1	DSM 671	AM774415-AM774419	OE	[1]	18313895	fpf@oe: seq,anno
Halobacterium salinarum	NRC-1	ATCC 700922	AE004437 AE004438 AF016485	VNG	[2] [3]	11016950 9847077	fpf: 3rd party anno BK010829-BK010831
Halobacterium salinarum	91-R6	DSM 3754	CP038631-CP038633	HBSAL	[4] [5]	31296677 31797576	fpf: seq,anno
Halobacterium hubeiense	JI20	HAMBI 3616	LN831302-LN831305	Hhub	[6]	26628271	fpf: anno
Haloferax volcanii	DS2	ATCC 29605	CP001953-CP001957	HVO	[7]	20333302	fpf: anno
Haloferax gibbonsii	LR2-5	-	CP063205-CP063208	HfgLR	[8]	33664716	fpf: anno
Haloferax mediterranei	R-4	ATCC 33500	CP001868-CP001871	HFX	[9]	22843593	none
Natronomonas pharaonis	Gabara	DSM 2160	CR936257-CR936259	NP	[10]	16169924	fpf@oe: seq,anno
Natronomonas moolapensis	8.8.11	DSM 18674	HF582854	Nmlp	[11]	23516216	fpf@oe: seq,anno
Haloquadratum walsbyi	HBSQ001	DSM 16790	AM180088-AM180089	HQ	[12]	16820047	fpf@oe: seq,anno
Haloquadratum walsbyi	C23	DSM 16854	FR746099-FR746102	Hqrw	[13]	21701686	fpf@oe: seq,anno
Natrialba magadii	MS3	ATCC 43099	CP001932-CP001935	Nmag	[14]	22559199	fpf: anno
Haloarcula marismortui	-	ATCC 43049	AY596290-AY596298	rrnAC rrnB pNG	[15]	15520287	none
Haloarcula hispanica	Y-27	ATCC 33960	CP002921-CP002923	HAH	[15]	21994921	none
Halorubrum lacusprofundi	ACAM 34	ATCC 49239	CP001365-CP001367	Hlac	[15]	27617060	none
Halohasta litchfieldiae	tADL	DSM 22187	-	halTADL	[16]	24082106	none

Table S2: Listing of the genomes which are manually curated and kept up to date. Each row lists one genome. (1) Three columns provide information about the biological source of the genome. These are the column Species, the column Strain, and the column Collection which reports a culture collection and the number in that collection. (2) The column Accessions lists the accession numbers in the GenBank and EMBL nucleotide sequence databases. (3) The column Locus tag prefix provides the term which precedes the underscore and serial number of the locus tag for that genome. This information can be used to back-translate from a locus tag to a genome and the strain and species from which the genome originates. (4) The reference describing

genome sequencing is provided in two columns. (4A) The column Reference links to the reference list. (4B) The column PMID lists the PubMed ID of the publication. (5) The column Contribution provides information about the contribution of one of us (F.P., tagged fpf) to genome sequencing and genome annotation. The term “fpf@oe” is used for genomes which have been sequenced in department Oesterhelt at the Max-Planck-Institute of Biochemistry. The term “seq_anno” indicates participation in genome sequencing and genome annotation, “anno” indicates participation in genome annotation. “none” indicates that the genome has been sequenced and annotated independently and has been adopted for manual curation independent from the original sequencing and annotation consortium. A species case is the genome of *Halobacterium salinarum* strain NRC-1, which has been sequenced and annotated independently, but our reannotation has been submitted to GenBank as a third party annotation.

Supplementray Text S1: Detailed background information for all open annotation issues

This text is organized so as to correspond with the decimal-numbered subsections (.1, .2, .3 etc.) of the Results segment (3) of the main text. For example, details for 3.2 Amino Acid Biosynthesis are in Section S2, and S2.c covers topic (c) in the results.

Section S1: The respiratory chain and oxidative decarboxylation

S1.a Ferredoxin-dependent oxidative decarboxylation

In haloarchaea, oxidative decarboxylation is not linked to reduction of NAD to NADH but to reduction of a 2Fe-2S ferredoxin (encoded by *fdx*) (e.g. OE_4217R, HVO_2995) which has a redox potential similar to that of the NAD/NADH pair [17]. The enzymes for oxidative decarboxylation are pyruvate--ferredoxin oxidoreductase (*porAB*) and 2-oxoglutarate--ferredoxin oxidoreductase (*korAB*), and these have been characterized from *Halobacterium salinarum* [18-20]. In *Haloferax*, a conditionally lethal *porAB* mutant was unable to grow on glucose or pyruvate, but could grow on acetate. This demonstrates that alternative enzymes for conversion of pyruvate to acetyl-CoA do not exist in this species [21].

S1.b Re-oxidation or reduced ferredoxin

It is yet unclear how the reduced ferredoxin Fdx (HVO_2995) is reoxidized. We speculate that the haloarchaeal Nuo complex may be responsible for ferredoxin reoxidation. Additional biological roles might be uncovered by further experimental analyses. In *Haloferax*, the same ferredoxin (HVO_2995) plays an essential role in nitrate assimilation [22], which also is a means for reoxidation of that ferredoxin. However, this is not applicable to organisms which lack the corresponding nitrate assimilation pathway, like *Hbt. salinarum*.

S1.c The haloarchaeal Nuo complex

Mitochondrial complex I functions as NADH dehydrogenase, transferring two electrons to the lipid electron carrier ubiquinone [23]. The equivalent in *Escherichia coli* is the Nuo complex which functions as NADH dehydrogenase [24,25]. Haloarchaeal genomes contain a *nuo* cluster with genes and gene order largely conserved to the gene set in *E. coli*. Apart from a few gene

fissions and fusions, the haloarchaeal *nuo* cluster is characterized by the absence of the three genes *nuoEFG*. The NuoEFG gene products function as a NADH adaptor and make this complex a NADH dehydrogenase [26,27]. Thus, the haloarchaeal Nuo complex is unlikely to reoxidize NADH, even though databases, e.g. KEGG (as of April 2021), assign this function. Our rejection of the haloarchaeal Nuo complex being annotated as NADH dehydrogenase is supported by experimental data which show that the haloarchaeal complex I is not involved in NADH oxidation [28]. Up to now, the substrate of the Nuo complex in haloarchaea has remained enigmatic but reduced ferredoxin Fdx is a reasonable candidate. The thermophilic cyanobacterium *Thermosynechococcus elongatus* has an equivalent complex which also lacks the *nuoEFG* genes and was shown to directly mediate oxidation of ferredoxin, which is reduced by photosystem I in this species [29-31].

S1.d Type II NADH dehydrogenase in halophilic archaea

Halophilic archaea do not use a type I enzyme for NADH oxidation but instead oxidize NADH via a type II NADH dehydrogenase [28]. For *Natronomonas pharaonis,* we have predicted that NP_3508A functions as type II NADH dehydrogenase [10]. The *Hfx. volcanii* ortholog is HVO_1578 (59% protein sequence identity). However, this assignment is highly questionable.

The assignment of NP_3508A as the *Nmn. pharaonis* type II NADH dehydrogenase was based on the enzyme from *Acidianus ambivalens* which at that time was considered to be a characterized type II NADH dehydrogenase [32,33]. There is, however, only 26% protein sequence identity, and this is even restricted to the N-terminal 140 of ca 400 aa. Even more problematic is the fact that the function assignment to the very distant homolog has subsequently been corrected and the enzyme is now known to be a sulfide:quinone oxidoreductase devoid of NADH dehydrogenase activity [34].

HVO_1578 and NP_3508A are distantly related to *E. coli ndh* but with only 24% protein sequence identity. *E. coli ndh* has been characterized as a type II NADH dehydrogenase [35]. NP_3508A is somewhat more closely related (30% protein sequence identity) to *Alkalihalobacillus (Bacillus) pseudofirmus* NDH-2A which has been functionally characterized [36]. The identification of the type II NADH dehydrogenase in halophilic archaea has to be

considered questionable and can only be clarified by experimental characterization. The assignment of type II NADH dehydrogenase activity to HVO_1578/NP_3508A and to HVO_1413) was also made in an extensive analysis of the NADH:quinone oxidoreductase family [37].

S1.e A canonical complex III is absent in many haloarchaea

While the overwhelming majority of haloarchaea code for the equivalent of complexes I and II (Nuo complex and succinate dehydrogenase), about one-third do not code for a complex III equivalent (cytochrome bc_1 complex encoded by *petABC*) according to OrthoDB analysis. While the bc_1 complex is found in most *Haloferacales* and *Halobacteriales*, it is absent in nearly all *Natrialbales*. However, it was also not found in the genus *Natronomonas*, which belongs to the *Halobacteriales*. In *Hbt. salinarum*, the genes for the three subunits (*petABD*) are clustered in the genome (OE_1876R/1874R/1872R) while in *Hfx volcanii* the *petBD* genes (HVO_0842/0841) are clustered and *petA* (HVO_2620) is solitary and separated by 1.1Mb.

The bc_1 complex is required to transfer electrons from the lipid-embedded two-electron carrier (menaquinone in haloarchaea) to the one-electron carrier associated with terminal oxidases (probably halocyanin). Most likely, electrons flow via an alternative pathway, which, however, is yet unresolved.

The haloarchaeal genes share some characteristics with those of the chloroplast b6-f complex rather than those of the mitochondrial bc_1 complex. In chloroplasts and in haloarchaea, the cytochrome b gene is split (chloroplast genes code for cytochrome b6, PetB, and the 17K subunit, PetD). As an example, *Haloferax* PetB shows 39% protein sequence identity to cytochrome b6 from *Synechococcus sp.* PCC7002 [38]. Also, the associated one electron carrier in chloroplasts is a copper protein (plastocyanin) as in haloarchaea (probably halocyanin, see below) and not a heme (and thus iron) protein (mitochondrial cytochrome-c).

S1.f An atypical "bc complex" from *Natronomonas pharaonis* and the claim that a novel way of covalent heme attachment may exist in haloarchaea

An atypical cytochrome bc has been isolated from *Nmn. pharaonis* [39]. While canonical bc_1 complexes have a 2:1 ratio of b-type to c-type cytochrome, the *Natronomonas* complex had an unusual 1:1 stoichiometry [39]. The enzyme was characterized but data allowing its assignment to a gene were not integrated into that publication. However, the authors mention unpublished amino acid composition data: "No Cys could be determined during the analysis of the amino acid composition after performic acid oxidation (data not shown), but since heme C is covalently bound via two cysteine residues to the protein, the hydrolysis product would escape the detection by amino acid analysis".

Sequence data are available from a PhD thesis [40]. Analyses attributed to two diploma theses resulted in a long nearly contiguous protein sequence (41 amino acids). In one experiment, the band from the SDS-PAGE was subjected to N-terminal protein sequencing. In the other attempt, the protein complex was digested with proteases (Glu-C, chymotrypsin) and the peptides were separated by HPLC. A peak absorbing at 400 nm (in addition to 280 nm) was considered to contain covalently attached heme and was subjected to protein sequencing. Two overlapping peptides were obtained in this experiment, and these also overlapped with the sequence obtained by N-terminal sequencing. In total, a region of 41 amino acids was sequenced with only the penultimate residue remaining unassigned. From this partial protein sequence, degenerated primers were designed and could be used successfully for PCR amplification [40]. The PCR product was used as probe to identify and clone a genomic restriction fragment coding for the corresponding gene. The translation of the gene was near-identical to the protein sequencing result, except for two mismatches [40]. The gene identified was *sdhD*, and was part of a four-gene operon coding for the four subunits of succinate dehydrogenase (respiratory complex II). The product of *sdhD* is one of the membrane subunits. Succinate dehydrogenase itself was characterized in the same paper that also described the cytochrome bc, and was found to contain b-type heme [39]. We list all subunits of this enzyme from *Hfx. volcanii* and *Nmn. pharaonis* in Suppl. Table S1.

Expecting the identified gene to code for a sequence with two Cys residues (see above), the authors were surprised to find that SdhD lacks cysteine residues, and concluded that their research strategy had failed and they had inadvertently isolated an unrelated gene. This

conclusion is expressed in the PhD thesis as “Looking at the remainder of the translated sequence of ORF 3, there is neither the described heme C binding motif nor any cysteine residue” and “nevertheless, the lack of a heme c binding motif and any cysteine in the translated sequence indicates that the isolated gene cannot represent a cytochrome-c”.

A similar attitude is conveyed in a recent review on haloarchaeal heme proteins [41] under the heading "The Haloarchaea Paradox". After summarizing descriptions of small cytochrome-c proteins in *Natronomonas*, *Halobacterium*, and *Haloferax*, the authors state, "This leads to the conclusion that they might not be found using similarity and/or pattern searches and that they use non-standard amino acid patterns and heme c linkage".

We speculate that all the described experiments in the Mattar thesis had been successful. In our view, it is well possible that haloarchaeal c-type cytochromes exemplify a novel type of heme attachment which is independent of Cys residues. In this context, it should be noted that the translation of the SdhD gene over the 41 residues that are covered by the protein sequencing results contains three His residues, none of which was identified upon Edman degradation. Two of the His residues correspond to the two mismatches, while the third corresponds to the residue which could not be called.

It is textbook knowledge and a paradigm that heme must be covalently attached via a pair of Cys residues. However, a paradigm shift may be necessary if haloarchaea have developed a cysteine-independent heme attachment mechanism. We hope that our detailed analysis helps to pave the way for an experimental challenge to the existing paradigm.

Next, we consider if the described small cytochrome-c from *Halobacterium* could also correspond to SdhD. This cytochrome c_{552} is reported to be 14.1 kDa. It was purified and covalent heme attachment was shown by heme staining after SDS-PAGE [42]. The original authors considered their cytochrome-c to be the functional equivalent of mitochondrial cytochrome-c [28,42]. The *Halobacterium* c_{552} is likely to correspond to the *Natronomonas* cytochrome bc for a number of reasons: (a) the *Halobacterium* cytochrome c_{552} was purified from membranes which had been solubilized with Triton X-100. The mitochondrial and bacterial

cytochrome-c which functions as one-electron carrier to cytochrome-c oxidase is a soluble and monomeric protein. (b) The *Halobacterium* cytochrome c_{552} contained considerable amounts of a b-type heme. The authors state that hydroxyapatite chromatography "resolved the preparation into fractions with different ratios of cytochromes" [42], which was interpreted as partially separating distinct proteins but an equally consistent interpretation is that a single protein complex might have undergone some degree of disaggregation. (c) The size of the c-type heme containing protein is in the same range (14.1 kDa vs 18 kDa), and the difference might be attributed to a comparably low size resolution for small proteins and the performance of the experiments in different laboratories. (d) A gene coding for a protein homologous to cytochrome-c has never been identified in any haloarchaeal genome. Taking all these points into consideration, there is good evidence to believe that *Halobacterium* c_{552} and *Natronomonas* cytochrome bc are corresponding proteins, and are both SdhD.

S1.g Halocyanin as candidate one electron carrier for cba-type terminal oxidase

The last step of the respiratory electron transport chain is catalyzed by terminal oxidase. There are two classes of terminal oxidases. Some terminal oxidases act directly on the lipid based two electron carrier, which is the product of complexes I and II. The majority of terminal oxidases, however, function with a one electron carrier. The classical (mitochondrial) one electron carrier is the heme protein cytochrome-c. In halophilic archaea, the one electron carrier is most likely halocyanin, a copper containing protein related to plastocyanin from chloroplasts.

A halocyanin from *Nmn. pharaonis* (NP_3954A) has been characterized, including its redox potential [43-45]. It is a protein which is TATLipo positive [46] and thus is secreted by the TAT pathway, followed by signal peptidase II cleavage and N-terminal lipid attachment to the conserved cysteine of the lipobox motif. Protein-chemical characterization identified the attached lipid and provided circumstantial evidence of the N-terminus being additionally acetylated [43,44]. The redox potential would be consistent with it being an electron donor to the cba-type terminal oxidase, but it is unknown if this halocyanin is the cognate electron donor.

The *cbaD* subunit of the cba terminal oxidase is a very short protein in *Natronomonas* (NP_2966A, 54 aa) [47]. In *Halobacterium*, this domain is fused with a pair of blue copper

domains (OE_4073R, 429 aa). In strain NRC-1, one of these domains has been cut out with surgical precision, while both domains are found in strains R1 and the type strain 91-R6 of *Halobacterium* [1,5]. In *Haloferax*, the Cba type terminal oxidase has a short CbaD subunit (HVO_0943, 97 aa) and no halocyanin gene is found in the genomic vicinity of the *cba* gene cluster. Experimental confirmation of the involvement of halocyanin in terminal oxidation by cba-type terminal oxidases is, to our knowledge, yet unavailable. The *Haloferax* halocyanin HVO_2150 is the most closely related to the N-terminal part of OE_4073R, and is thus a candidate to be the electron donor to the cba-type terminal oxidase.

S1.h Terminal oxidases in haloarchaea

Mitochondrial complex IV is the terminal oxidase which transfers electrons from the one electron carrier cytochrome-c to oxygen, finally resulting in the generation of water [23,48]. Halophilic archaea code for a diverse set of terminal oxidases [10,49,50]. Some probably function with one electron carriers, similar to those of mitochondria. However, the one electron carrier is not a heme protein (cytochrome-c) but a copper protein (halocyanin). It cannot be excluded that some halophilic archaea code for terminal oxidases which are able to directly reoxidize the two electron carrier menaquinone.

In three haloarchaea (*Nmn. pharaonis*, *Hfx. volcanii*, *Hbt. salinarum*) at least one terminal oxidase has been experimentally studied and we restrict our analysis to terminal oxidases in these species. All three species code for additional, yet uncharacterized, terminal oxidases, which are also covered.

Nmn. pharaonis codes for three terminal oxidases, of which one belongs to the ba_3-type (encoded by *cbaABDE*) and has been experimentally characterized [47]. Each subunit has an ortholog in *Hfx. volcanii*, which includes a short ortholog of CbaD and an ortholog of CbaE. The long subunits have an ortholog in *Hbt. salinarum*. This species has CbaD fused to a halocyanin with a pair of blue copper domains (OE_4073R) (see above, halocyanin). An ortholog of CbaE is missing in *Hbt. salinarum*. The subunit structure in *Natrialba magadii*, which has two copies of

this terminal oxidase, corresponds to that of *Nmn. pharaonis,* while the subunit structure in *Haloarcula marismortui* corresponds to that of *Hbt. salinarum*.

Another terminal oxidase of *Nmn. pharaonis* belongs to the aa_3-type and consists of three subunits (according to InterPro domain assignments) which are located nearby on the genome (CoxA1, NP_2456A, CoxB1, NP_2448A, CoxC1, NP_2450A). Its long chain (subunit I, CoxA1) is orthologous to the long chain of the characterized aa_3-type terminal oxidases of *Hfx. volcanii* (CoxA1, HVO_0907, 68% protein sequence identity) and to CoxA1 of *Hbt. salinarum* (OE_1979R, 65% protein sequence identity). The characterized enzymes from these species were probably partially disintegrated upon solubilization and purification as only two subunits were found for the *Haloferax* enzyme (only CoxA1 sequenced and thus assigned to a gene) [51] and only a single subunit was found for the *Halobacterium* enzyme [52,53]. However, there are orthologs to *Natronomonas* CoxB1 and CoxC1 in both species. In *Halobacterium,* the genes for the three subunits are located nearby on the genome (CoxA1, OE_1979R, CoxB1, OE1988R, CoxC1, OE1984F). In *Haloferax*, the three subunits are separated from each other (CoxA1, HVO_0907, CoxB1, HVO_1014, CoxC1, HVO_1138). In all three species, the *coxB1* gene is directly adjacent to that of heme o synthase (encoded by *ctaB*).

Hfx. volcanii and *Hbt. salinarum* also contain a cytochrome bd ubiquinol oxidase (encoded by *cydAB*) which is absent from *Nmn. pharaonis*. CydA is distantly related to the characterized enzyme from *Azotobacter vinelandii* and slightly more distant to that of *E. coli*. CydAB directly oxidizes the intramembrane two-electron carrier to the corresponding quinone (ubiquinone in *E. coli* and *A. vinelandii*, menaquinone in haloarchaea).

Hfx. volcanii codes for a fourth terminal oxidase, which does not have an ortholog in the other two species, exemplifying the variability of respiratory chains in haloarchaea. It resembles the characterized aa_3-type terminal oxidase of *Aeropyrum pernix* [54]. This terminal oxidase is characterized by the fusion of the genes for subunits 1 and 3 (encoded by *coxAC2*, HVO_1645),

a subunit 2 (encoded by *coxB2*, HVO_1646) and an additional small subunit (encoded by *coxD2*, HVO_1644).

Nmn. pharaonis also codes for another terminal oxidase (*coxA3*, NP_4296A, *coxB3*, NP_4294A) which does not have an ortholog in *Hbt. salinarum* or *Hfx. volcanii*. This is very distantly related to CbaAB (NP_2962A/2964A). The closest SwissProt homolog (from *Thermus thermophilus*) is more closely related to the cognate CbaAB. Among the 16 haloarchaeal genomes which are under survey [55] (Suppl. Table S2), only *Halobacterium hubeiense* has orthologs with >55% protein sequence identity.

S1.j NAD-dependent oxoacid dehydrogenase complexes

In *E. coli*, pyruvate is oxidized to acetyl-CoA by the classical pyruvate dehydrogenase complex, which results in NADH that is subsequently reoxidized by complex I (NADH dehydrogenase).

In halophilic archaea, pyruvate is oxidized to acetyl-CoA exclusively via a ferredoxin-dependent mechanism, while NAD(P)-dependent pyruvate oxidation could not be detected in cell homogenates [17]. The enzyme (pyruvate--ferredoxin oxidoreductase; EC 1.2.7.1; OE2622R+OE2623R) has been characterized [18]. An equivalent reaction oxidizes alpha-ketoglutarate to succinyl-CoA (oxoglutarate--ferredoxin oxidoreductase; EC 1.2.7.3; OE1711R+OE1710R) [19,20]. Further confirmation that NAD-dependent enzymes are not involved in conversion of pyruvate to acetyl-CoA was obtained by deletion of the *Haloferax porAB* genes. The deletion strain loses the ability to grow on glucose or pyruvate as a single carbon source, but still can grow on acetate [21].

Haloarchaeal genomes code for distant homologs of the classical pyruvate dehydrogenase complex. Because NAD was experimentally shown not to be involved in oxidative decarboxylation of pyruvate or alpha-ketoglutarate, these enzymes must act as 2-oxoacid dehydrogenases using substrates other than pyruvate and alpha-ketoglutarate. In *Haloferax*, several studies have attempted to unravel the substrate, but only with partial success. For example, in one study [56], the authors wrote: "Enzyme activities: In the context of our

discovering genes for a putative ODHC" (Oxoacid DeHydrogenase Complex), "it is worth reiterating that we and others have never detected enzymic activities of PDHC" (Pyruvate DeHydrogenase Complex), "OGDHC" (OxoGlutarate (which is alpha-ketoglutarate) DeHydrogenase Complex) "or the BCODHCs" (Branched Chain Oxoacid DeHydrogenase Complex) in *H. volcanii* or any other halophilic archaeon. However, DHLipDH" (dihydrolipoate dehydrogenase) "activity is present in all strains tested. Assays for the ODHCs have been repeated with the strain of *H. volcanii* used in this work and the absence of enzymatic activity has been reconfirmed. Positive controls for these activities have been performed with cell extracts of *E. coli*." Several other publications have attempted to unravel the substrate of the oxoacid dehydrogenase complexes by analyzing three of the four E1 components without any success [57-59]. Not only were enzyme assays attempted, but also the growth of *Haloferax* with pyruvate and/or alpha-ketoglutarate as sole carbon source was analyzed. Only one publication successfully identified the substrate of one of the oxoacid dehydrogenase complexes [60]. For three of the four genes known at that time to encode an E1 alpha subunit, a number of gene deletions were generated: single gene deletions, all pairs of double deletions, and a triple deletion. Growth on isoleucine was impaired in all deletion strains lacking the *oadh1* gene (HVO_2958). Metabolomic analyses showed that these strains failed to metabolize isoleucine as well as its deamination product while the parent strain and those deletion strains that retain the *oadh1* gene were unaffected. However, even though the combination of experimental results makes the substrate assignment unambiguous, it was not possible to establish an enzyme assay for *Haloferax*, even though the activity of the positive control used in their assays (*Pseudomonas putida* branched-chain OADHC) could be readily measured. The *Haloferax* oxoacid dehydrogenase complexes seem notoriously difficult to study.

The identification of closely related experimentally characterized homologs (GSPs) is not straight-forward due to database incompleteness. HVO_2958 (encoded by *oadA1*) and HVO_2209 (encoded by *oadA4*) are equidistant to a characterized homolog from *Thermoplasma acidophilum* (38% protein sequence identity). That enzyme is involved in the degradation of all three branched chain amino acids [61], while HVO_2958 was proposed to be specific for Ile and HVO_2209 has not yet been characterized as its existence could not be anticipated prior to sequencing of the complete genome. The *Thermoplasma* proteins are still unreviewed in UniProt

(as of April 2021), but a bibliography submission has been provided using the newly introduced community feedback system of UniProt. HVO_0669 (encoded by *oadA3*) is related to the E1 component of a TPP-dependent acetoin dehydrogenase from *Bacillus subtilis* (54% protein sequence identity) and from *Pelobacter carbinolicus* (49% protein sequence identity), both being characterized [62,63]. These are also top matches for HVO_2595 (encoded by *oadA2*) (40-41% protein sequence identity), but only the *B. subtilis* homolog is incorporated into the SwissProt section of UniProt (accessed April 2021). Given database incompleteness, it cannot be excluded that more closely related characterized homologs exist.

Despite strong evidence which excludes pyruvate and alpha-ketoglutarate as substrates, KEGG insists (despite corresponding feedback) to maintain the annotation of these proteins as pyruvate dehydrogenase (EC 1.2.4.1) (accessed April 2021). According to our correspondence with KEGG, this will only be discontinued when the haloarchaea community is able to provide positive identification of the true biological substrate.

To provide further information, we list in Suppl. Table S1 all components of oxoacid dehydrogenase complexes, the alpha and beta chain of component E1, component E2 including the short (86 aa) lipoyl domain protein, and the single component E3. We thus highlight gene clusters and indicate sequence similarity between paralogs.

Section S2: Amino acid metabolism

S2.a Reconstruction of arginine and lysine biosynthesis

Arginine biosynthesis starts from glutamate with phosphorylation of the gamma-carboxylic group. To avoid unfavorable intramolecular circularization, the alpha-amino group must be protected, with the key intermediate metabolite ornithine being released by deprotection. The protecting group is part of the enzyme name for the pathway reactions between glutamate and ornithine, and thus, a reliable annotation requires a correct assignment of the protection mode. We are confident that haloarchaea use a carrier-mediated approach for arginine biosynthesis, but there is yet no experimental proof. The assignment is based on experimentally characterized homologs, which, however, are additionally or even exclusively involved in lysine biosynthesis.

Thermococcus kodakarensis, *Sulfolobus acidocaldarius* and *T. thermophilus* all synthesize lysine via the alpha-aminoadipate pathway using a carrier protein for protection of the alpha-amino group. Thus, they use a carrier protein (encoded by *lysW*) and a ligase (encoded by *lysX*). In *Thermococcus*, all enzymes of the arginine and lysine biosynthesis pathway are bifunctional (promiscuous) and are involved in both pathways [64]. In *Sulfolobus*, the same carrier protein (LysW) is used for both, lysine and arginine biosynthesis [65], but the ligases which attach the substrate are distinct (being encoded by *argX* and *lysX*, respectively) [65]. In *Thermus*, the LysX/LysW pair has been characterized [66]. In UniProt, this reference has been mis-associated with the proteins from strain HB8 instead of HB27 (accessed March 2021). The LysW protein from strain HB27 (TT_C1544, Q72HE5) has the codon for Val- 23 incorrectly assigned to be the start codon. After sequence extension, there is 57% protein sequence identity to HVO_0047.

Halophilic archaea do not use the alpha-aminoadipate pathway for lysine biosynthesis but instead use the diaminopimelate pathway [67], which does not require protection of the alpha-amino group. The genes for carrier protein and ligase are encoded in the arginine biosynthesis cluster. We thus assign gene names *argW* and *argX* rather than *lysW* and *lysX* (as in *Thermococcus*, *Sulfolobus*, and *Thermus*). However, experimental confirmation of these predictions is not yet available. Also, no genes have been assigned for conversion of alpha-ketoglutarate to alpha-aminoadipate in haloarchaea, which would be a prerequisite for involvement of the *arg* cluster genes in lysine biosynthesis via the alpha-aminoadipate pathway. It should be noted that KEGG also does not assign two of the four enzymes for *T. kodakarensis* (accessed April 2021), even though *Thermococcus* is known to use that pathway [64].

Because most of the enzymes for arginine biosynthesis are related to bifunctional enzymes, we provide the reconstruction of the complete pathway in Table 2, where genes are arranged along the biosynthesis pathway according to the enzymes they encode. It has been observed experimentally that the level of arginine biosynthesis enzymes decreases with increased temperature [68]. Additionally, we provide an extensive reconstruction of the lysine biosynthesis pathway in haloarchaea in Table 2. There is one gap in the pathway (EC 2.6.1.17, *dapC*, *argD*). One of the generally annotated pyridoxal phosphate-dependent aminotransferases may code for this reaction, but there is none in the genomic vicinity. Several haloarchaea lack homologs to

dapF and/or to *dapE*. It should also be noted that KEGG assigns DapE function also to HVO_A0634 (accessed March 2021). We consider this unlikely because it's genomic position is not adjacent to other genes of the pathway, and because it shows low similarity to a GSP.

S2.b Aromatic amino acid biosynthesis

Methanocaldococcus jannaschii enzymes MJ0400 and MJ1249 have been functionally characterized and shown to be involved in the archaea specific part of the shikimate pathway for aromatic amino acid biosynthesis (2-amino-3,7-dideoxy-D-threo-hept-6-ulosonate synthase, EC 2.2.1.10 and 3-dehydroquinate synthase, EC 1.4.1.24) [69]. Orthologs in halophilic archaea (encoded by *fba2*) are OE_1472F from *Hbt. salinarum* and HVO_0790 from *Hfx. volcanii* for MJ0400 and OE_1475F and HVO_0792 for MJ1249. OE_1472F and OE_1475F have been partially characterized [70]. Gene deletion was not possible but transcription could be strongly diminished [70]. This resulted in poor growth in the absence of aromatic amino acids, supporting the involvement in aromatic amino acid biosynthesis. According to the pathway in *M. jannaschii*, MJ0400 should condense aspartate semialdehyde with 6-deoxy-5-ketofructose (DKFP). Based on circumstantial evidence (see below), an alternative reaction is proposed where aspartate semialdehyde is condensed with fructose-1,6-bisphosphate (F-1,6-P) rather than DKFP. This would result in an alternative product that, however, could be converted to an identical compound, 3-dehydroquinate, in the subsequent step, because the part where the molecules differ would not be incorporated into the product. Reasons to propose a different substrate are: (a) difficulties in reconstructing a pathway leading to DKFP in *Halobacterium* and (b) the ability to detect binding of fructose-1,6-bisphosphate to MJ0400 by X-ray structural studies which involve soaking of the crystal with fructose-1,6-bisphosphate [71] and (c) the observation by White [69] that DKFP, incubated with labelled glucose, "only slightly decreased the extent of label incorporation into the products". White concludes "This result would indicate that that DKFP may not be the preferred precursor to Compound 1". F-1,6-P has been tested only in combination with labelled pyruvate and with L-homoserine (no product formation with either), but not in combination with aspartate semialdehyde.

Unfortunately, the establishment of an in vitro assay for the condensation of F-1,6-P with aspartate semialdehyde for the *Halobacterium* protein was not successful. Thus, the proposed

alternative reaction for haloarchaeal biosynthesis of aromatic amino acids remains unconfirmed. A clean deletion of the *Haloferax* ortholog HVO_0790 has been generated (Maupin-Furlow, personal communication), but this strain has not been further characterized. Thus, the initial reactions in aromatic amino acid biosynthesis are a persisting open issue which awaits experimental confirmation.

S2.c Tryptophan degradation

The gene for tryptophanase (*tnaA*) is stringently regulated in *Haloferax*, which is the basis for using its promoter in the genetic toolbox for regulated gene expression [72]. Clearly, tryptophan should only be degraded when available in the medium. Tryptophanase can then cleaves Trp to NH_3, pyruvate, and indole. However, the fate of the resulting indole remains unknown. None of the indole conversions represented in KEGG are assigned to a protein from *Hfx. volcanii* (as of April 2021).

S2.d Histidine utilization

A probable histidine utilization cluster exists, based on distant homologs from *B. subtilis* [73-76]. The set of four clustered genes (HVO_A0562 – HVO_A0559) would code for a complete pathway that converts histidine to glutamate. To our knowledge, growth of *Hfx. volcanii* on histidine has not yet been characterized and the enzymes have not yet been experimentally verified. Previously we had observed an enhanced level of enzymes involved in histidine utilization under high-salt conditions [68].

S2.e Auxotrophic mutants from a transposon insertion library with yet unassigned function

Among 16 auxotrophic mutants observed in a *Hfx. volcanii* transposon insertion mutant library [77], some could grow only in the presence of one (or several) supplied amino acids. Besides disruption of genes already known to be involved in the biosynthesis of the required amino acid (e.g. *argB*, *argC*, *carA*), some of the targeted genes do not yet have an assigned function. These are listed here.

Targeting of HVO_0431 (HAD superfamily hydrolase) makes *Haloferax* auxotrophic for histidine. This fits well to what currently is considered the only remaining pathway gap of

histidine biosynthesis (histidinol-phosphatase). However, the enzyme catalyzing the preceding step (HVO_1295) had its function assigned because it can complement a histidine auxotrophic mutation and shows distant similarity to *E. coli* histidinol-phosphate aminotransferase (EC 2.6.1.9) encoded by *hisC* (31% protein sequence identity). This level of sequence identity does not allow substrate assignments for this protein family (pyridoxal-phosphate dependent aminotransferase). To the best of our knowledge, it has not been experimentally confirmed if HVO_1295 catalyzes the reaction of EC 2.6.1.9. The gene coding for HVO_1295 is part of a highly conserved three-gene operon, with at least one of the other members being involved in polar lipid biosynthesis (see below, Section 7). It cannot be excluded that the complementation of histidine auxotrophy is due to a side activity of an enzyme with reduced substrate specificity which primarily functions in polar lipid biosynthesis. In that case, even, it might be possible that haloarchaea do not use the classical histidine biosynthesis pathway where the product of EC 4.2.1.19 (imidazole-acetol phosphate) is first transaminated (by EC 2.6.1.9, histidinol-phosphate aminotransferase) and then dephosphorylated (by EC 3.1.3.15, the pathway gap described above). These reactions might be swapped in haloarchaea. Only experimental analysis can clarify whether HVO_1295 functions as histidinol-phosphate aminotransferase and HVO_0431 functions as histidinol-phosphatase.

HVO_0644 is annotated as 2-isopropylmalate synthase / (R)-citramalate synthase (EC 2.3.3.13 and EC 2.3.1.182, respectively). The two reactions are chemically similar and thus could be catalyzed by an enzyme with relaxed substrate specificity. (R)-citramalate synthase is an early step of isoleucine biosynthesis and transposon insertion into HVO_0644 makes *Haloferax* auxotrophic for isoleucine [77]. It is yet unresolved if HVO_0644 (encoded by *leuA1*) is really bifunctional, having 2-isopropylmalate synthase activity and thus being involved in leucine biosynthesis. This activity is assigned to the distant paralog HVO_1510 (encoded by *leuA2*, 39% protein sequence identity). HVO_1510 belongs to an ortholog set for which the start codon assignment is very difficult (see below, S2.f).

Targeting of HVO_1153 makes *Haloferax* auxotrophic for proline. The characteristics of that protein provide no link to proline biosynthesis. The protein has an N-terminal transmembrane domain but tools to detect secretion signals (Sec, Tat, Fla) are negative. The protein may be O-

glycosylated (positive with NetOGlyC) and thus may belong to the secretome. Thus, this protein is currently annotated as "probable secreted glycoprotein", the same annotation being used for the adjacently encoded HVO_1154 and HVO_1155. In this case, a standard gene deletion should be generated to confirm the phenotype of the transposon insertion library. It should be noted that transposon insertion mutants are relatively frequently associated with secondary genome alterations [78], and the observed phenotypes might be caused by these rather than the transposon insertion event.

S2.f Difficulty in finding a phylogenetically consistent start codon for a conserved leucine biosynthesis gene may indicate that the translation start is atypical

HVO_1510 is annotated as 2-isopropylmalate synthase (EC 2.3.3.13) (also in KEGG as of April-2021), which is the first reaction specific for leucine biosynthesis, just after that pathway branches off from valine biosynthesis. This activity is also assigned as one of two activities to HVO_0644, the other being a chemically similar reaction (EC 2.3.1.182) of isoleucine biosynthesis.

HVO_1510 belongs to an ortholog set with a major difficulty in start codon assignment. Each of the genomes under survey (Suppl. Table S2) has an ortholog (with more than 68% protein sequence identity), sequence similarity starting between pos 27 and 45. The most N-terminal peptide of HVO_1510 which was identified in the ArcPP project [79] is a tryptic peptide starting at pos 62, the next in-frame start codon (when accepting ATG, GTG, or TTG) is the codon for Met-93. Given the high coverage obtained for that protein, it is remarkable that the 18 aa peptide from pos 43-61 has not been identified, which might indicate that this peptide does not exist in the protein. Other peptides in this region cannot be considered as they are too small for proteomic detection, a consequence of the large number of arginine residues. The genomes from *Haloferax gibbonsii* strains LR2-5 (CP063205) and ARA6 (CP011947) are near-identical at the DNA and protein level but only strain LR2-5 has a start codon (GTG) assignment consistent with *Hfx. volcanii* HVO_1510 while in ARA6 the corresponding codon is GTA (Figure S1). This makes it very unlikely that the currently assigned GTG is truly the start codon of HVO_1510. The genome of *Haloferax mediterranei* is near-identical at the DNA level starting from base 23 but lacks similarity to bases 1-22. The region from pos 24 to 91 has a near-identical DNA

sequence but contains two in-frame stop codons in *Hfx. mediterranei* (Figure S1). Thus, the ortholog HFX_1573 is annotated as a disrupted gene, starting with Leu-27 (having a CTG, not a TTG codon) but being N-terminally truncated. Further examples are provided in Figure S1, where the *Hfx. volcanii* DNA sequence (codons 3 to 58, centered at codon 27) is compared to the genomes from 20 other strains from the genus *Haloferax*. *Hfx. mediterranei* is not exceptional in this respect, but the described pattern is also found in several other strains from the genus *Haloferax*. In Figure S1, deviating bases are highlighted by coloring, and it is evident that the region upstream of codon 27 is much more diverse than the region thereafter. The conserved sequence starts with a strictly retained CTG codon, which would code for Leu if an internal codon and for Met if used as an atypical start codon. We also note a strictly retained out-of-frame ATG close to the start of the conserved region. Its usage as translation start would necessitate ribosomal slippage, which might be employed for translational regulation. A potential transcription start was mapped ca 10 nt after codon 27, but the applied method is prone to limited positional uncertainty. The comparison to genera other than *Haloferax* is possible at the protein sequence level due to strong sequence conservation while there is a higher diversity at the DNA sequence level. Sequence similarity to orthologs from *Haloquadratum*, *Haloarcula* and *Natrialba* starts with Cys-30 of HVO_1510, which corresponds to Cys-10 of HQ277A/Hqrw_3051, Cys-8 of rrnAC0329/HAH_1067, and Cys-18 of Nmag_0906. Similarity starts even later for orthologs from *Halobacterium*. Glu-45 of HVO_1510 corresponds to Glu-7 of Hhub_3057 from *Hbt. hubeiense* and Phe-46 corresponds to Phe-6 of HBSAL_05970 from strain 91-R6^T of *Hbt. salinarum*. The orthologs from *Natronomonas* are closely related from Arg-36 of HVO_1510 (Arg-4 of NP_2206A and Nmlp_3164) but lack an upstream start codon (the next alternative being the codon for Met-60). The ortholog from *Halohasta litchfieldiae* starts with Cys-30, also lacks an upstream start codon, and the next alternative start codon is that for Met-63. It can be considered highly unlikely that this gene is disrupted in such a large number of haloarchaea. The other genes of the leucine specific part of the biosynthetic pathway are encoded in those genomes, including the gene for the first such enzyme that would have independently become nonfunctional. Thus, it is tempting to speculate that this gene is functional and that it is translated from an atypical start codon. This would allow for a regulatory mechanism where translation occurs only under conditions of an insufficient leucine supply. Conserved in-frame CTG and out-of-frame ATG codons were observed by DNA sequence

comparison to other genomes from *Haloferax* (Figure S1) but experimental analysis will be needed to clarify the true start codon used.

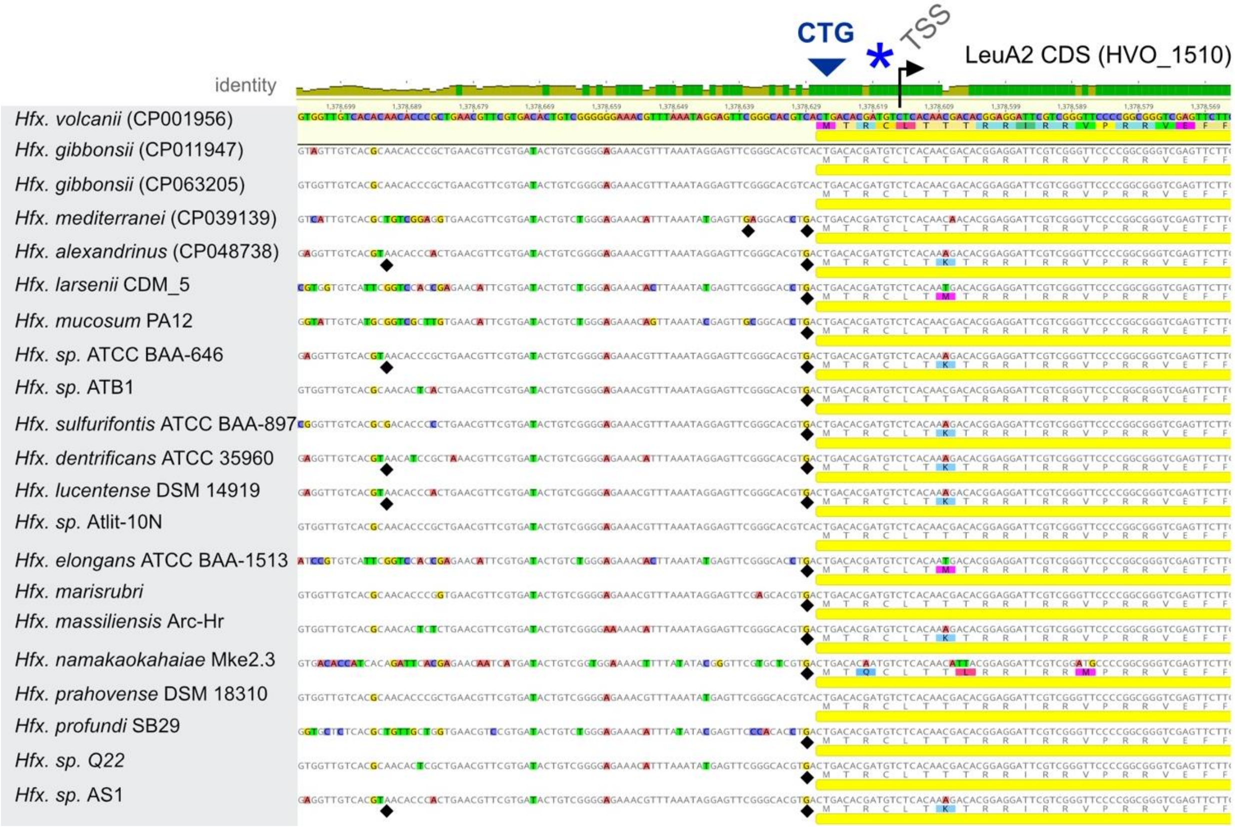

Figure S1. Alignment of the 5' end of *leuA2* (HVO_1510) from *Hfx. volcanii* DS2 with homologs from other species of *Haloferax*. Translation given in single letter code. In the comparison sequences below *Hfx. volcanii*, colored bases (and amino acids) indicate where differences occur. It is evident that colored bases are more frequent in the left half of the alignment. The dark blue arrow marks a conserved CTG codon which might serve as a start codon (tentatively translated to Met, corresponds to Leu-27 of HVO_1510 as currently annotated; the currently assigned start codon is the GTG at the beginning of the sequence). Black diamonds show positions of in-frame stop codons, which are found exclusively upstream of the CTG. The light blue asterisk marks a conserved, out of frame ATG. Its usage as a start codon would necessitate ribosomal slippage, which might be used for translation regulation. The arrow marks the transcription start site (TSS) of *leuA2* as determined by [80]. Even though the CTG is 10-12 bp upstream and the ATG is 2-4 bp upstream of the TSS, either of these codons may still be potential start codons given that the method for TSS determination carries some positional uncertainty.

Section S3: Coenzymes I: cobalamin and heme

Besides the canonical heme biosynthesis pathway (with the intermediate coproporphyrinogen III), two additional pathways exist. Halophilic archaea use the "alternative heme biosynthesis pathway" (the AHB pathway with the intermediate siroheme) [81,82], which has been reconstructed [14]. The third heme biosynthesis pathway (the CPD pathway with the intermediate coproporphyrin III) occurs in many bacteria [83] and shares its last pathway step with the AHB pathway.

De novo cobalamin biosynthesis is a multistep process, which occurs in two variants for the conversion of precorrin-2 to the corrin ring intermediate cobyrinate a,c diamide [84]. The cobalt-late (aerobic) pathway uses cobalt-free substrates, while the cobalt-early pathway uses cobalt-containing substrates. Many of the pathway intermediates are equivalent except for the absence/presence of the central cobalt atom, and the enzymatic reactions thus correspond closely to each other and are typically catalyzed by homologous enzymes. An exception is a key reaction which is chemically distinct, being catalyzed by CbiG (cobalt-early) or CobG (cobalt-late). The central cobalt atom plays an important role in the reaction catalyzed by CbiG [85]. In the cobalt-late (aerobic) pathway, the reaction is catalyzed by CobG and involves oxidation by molecular oxygen. The presence of a transition metal (cobalt) may not be suitable for this type of reaction.

The reactions beyond ligand cobyrinate a,c diamide are not primarily related to the corrin ring system but with the generation and attachment of the axial ligands. Some of these reactions are also involved in corrin ring salvage.

Both, cobalamin and heme biosynthesis pathways have been reconstructed during manual curation of haloarchaeal genomes [55]. Cobalamin biosynthesis has only few pathway gaps remaining. A proteomic study of *Hfx. volcanii* found a lower level of de novo cobalamin biosynthesis enzymes at increased temperature [68]. For heme biosynthesis, some pathway details are yet unresolved.

S3.a Currently, all *cbiX* gene paralogs are annotated as cobalt chelatase for cobalamin biosynthesis, but one may be an iron chelatase for heme biosynthesis

Haloarchaeal genomes code for homologs of characterized early cobaltochelatases (CbiX-type, e.g. from *Bacillus megaterium* [86], *Methanothermobacter thermoautotrophicus* [87], or *Archaeoglobus fulgidus* [88]). CbiX-type chelatases may be composed of a single or a pair of CbiX domains. Most haloarchaeal genomes code for two paralogous CbiX-type chelatases. Some species (e.g. *Nmn. pharaonis*, *Har. marismortui* and *Hbt. salinarum*) code for an additional CbiX domain protein.

Nab. magadii lacks genes for de novo cobalamin biosynthesis, so it was unexpected to find an early cobaltochelatase in this species [14]. The *Natrialba* protein (Nmag_3212) is a close ortholog of HVO_1128. Thus, it can be speculated that HVO_1128 and its close orthologs from other halophilic archaea do not function in cobalamin biosynthesis. It can be predicted that they function in siroheme biosynthesis as sirohydrochlorin ferrochelatase (EC 4.99.1.4). This prediction awaits experimental confirmation.

It should be noted that the products of the *cbiK* gene in *Salmonella* [89] and *Desulfovibrio* [90] were found to function as both cobaltochelatase in cobalamin biosynthesis and ferrochelatase in siroheme biosynthesis.

An alternative enzyme for sirohydrochlorin ferrochelatase activity is HVO_2312, which functions as precorrin-2 dehydrogenase but additionally may have sirohydrochlorin ferrochelatase activity. *E. coli* and *Salmonella* CysG is a trifunctional enzyme consisting of two domains. The N-terminal domain is bifunctional having precorrin-2 dehydrogenase as well as sirohydrochlorin ferrochelatase activities [91-93]. The homologous *B. megaterium* SirC is, however, a monofunctional precorrin-2 dehydrogenase devoid of chelatase activity, the latter having been attributed to SirB [86]. In the case of *Methanosarcina barkeri* (Mbar_A1461), precorrin-2 dehydrogenase was confirmed but sirohydrochlorin ferrochelatase activity was not analyzed [94]. Only experimental analysis will allow to resolve whether HVO_2312 has sirohydrochlorin ferrochelatase activity.

S3.b Two consecutive pathway gaps in cobalamin biosynthesis and four genes with unassigned function in the cobalamin gene cluster

The cobalamin biosynthesis pathway has been reconstructed during manual curation of haloarchaeal genomes [55]. As this reconstruction has not been described elsewhere, we list the associated genes and the underlying GSPs in Table 3, genes being ordered along the biosynthetic pathway. It is evident that most genes coding for the enzymes of the first part of the de novo cobalamin biosynthesis (upstream of cobyrinate a,c diamide) are clustered in the genome. In *Hfx. volcanii*, they are encoded on megaplasmid pHV3.

After detailed pathway reconstruction, two gaps remained open (EC 2.1.1.195 and 1.3.1.106). These enzymes catalyze consecutive steps which together result in conversion of cobalt-precorrin-5B to cobalt-precorrin-6B with cobalt-precorrin-6A being the intermediate. The first is a methyltransferase step, acting on C-1, while the second is a reduction, affecting the double bond between C18 and C19. It is quite possible that the order is inverted in haloarchaea, which would result in a novel intermediate. The cobalamin biosynthesis cluster contains four genes with yet unassigned function: HVO_B0052 (PQQ repeat protein), HVO_B0053 (DUF3209 domain protein), HVO_B0055 (conserved hypothetical) and HVO_B0056 (probable 4Fe-4S ferredoxin). A proteomic study found a decreased level of HVO_B0052 and HVO_B0055 at increased temperature, as found for the other enzymes of cobalamin biosynthesis [68].

SynTax analysis was performed in order to analyze if these genes are typical members of the cobalamin biosynthesis cluster. This cluster is plasmid-encoded (pHV3) in *Hfx. volcanii* and SyntTax only considers the main chromosome. Nevertheless, this analysis was possible as *Haloferax alexandrinus* strain wsp1 codes for an equivalent cluster which is chromosomally encoded, codes for highly conserved proteins (90-99% protein sequence identity) and gene order is identical (analyzed from *cbiG* (HVO_B0059, G3A49_06890) to *cbiE* (HVO_B0048, G3A49_06945). This region covers the four genes with yet unassigned function. SyntTax analysis uncovered that the four genes are strictly coupled to the cbiTLFGH1H2 operon in the majority of haloarchaeal genomes.

As this strengthens their status as candidates for closing the pathway gaps, we analyzed if their characteristics (e.g. domain architecture) would be compatible with the enzymatic conversions of the two pathway gaps. One missing enzyme is an oxidoreductase. It is obvious that a 4Fe-4S ferredoxin (HVO_B0056) may be involved in this reaction. HVO_B0052 is annotated as PQQ repeat protein. Such proteins probably use pyrroloquinoline quinone (PQQ) as prosthetic group, as e.g. found in quinoprotein alcohol dehydrogenases, making HVO_B0052 another top candidate for the missing reaction. The other two proteins (HVO_B0053, having a domain of uncharacterized function, DUF3209 and HVO_B0055, uncharacterized and devoid of any domain assignment), did not reveal any functional insights.

The other pathway gap is one of the many methyltransferases which are involved in cobalamin biosynthesis. In this context it should be noted that currently two paralogous enzymes are annotated to be responsible for C17 methylation: HVO_B0058, encoded by *cbiH1*, and HVO_B0057, encoded by *cbiH2*. These paralogs have 38% protein sequence identity to each other, with CbiH2 being much closer to characterized homologs. It may well be that CbiH1 is not responsible for methylation of C17 but of C1.

S3.c Cobalamin biosynthesis and salvage reactions beyond cobyrinate a,c diamide

Beyond the ligand cobyrinate a,c diamide are reactions which decorate the corrin ring (adenosylation, aminopropanol arm attachment, assembly of the nucleotide loop). The involved enzymes with their GSPs are listed in Table 3 (along the biosynthetic pathway with enzymes that prepare the ligands for attachment following after those directly acting on the corrin ring). For several of the enzymes, only a distant homolog has been characterized. Four enzymatic reactions need to be considered in more detail.

Two of these reactions are involved in the assembly of the nucleotide loop. In *Salmonella*, nicotinate-nucleotide-dimethylbenzimidazole phosphoribosyltransferase (encoded by *cobT*, EC 2.4.2.21) has been characterized [95]. Upon detailed bioinformatic analysis, this function has been assigned in *Halobacterium* [96]. Based on the adjacent genes shown in Table 1 of that paper, this assignment is for VNG_1572C, which shows 57% protein sequence identity to its *Haloferax* ortholog HVO_0590. This function assignment is circumstantial and has not yet been

confirmed experimentally. It should be noted that *Hht. litchfieldiae* has a much closer homolog to *Salmonella* CobT with 39% protein sequence identity (halTADL_3045, now known as SAMN05444271_10280). *Halohasta* lacks an ortholog of HVO_0590.

The subsequent reaction is catalyzed by adenosylcobalamin/alpha-ribazole phosphatase (encoded by CobC, EC 3.1.3.73), as shown in *Salmonella* [97]. No homolog had been identified in *Halobacterium* [96]. This was attributed to non-orthologous displacement and a candidate gene from the *Halobacterium* cobalamin cluster was proposed for this function (labelled HSL01294, Table 5 of that paper) [96]. Based on the adjacent genes shown in Table 1 of that paper, this assignment is for VNG_1577C, which shows 56% protein sequence identity to its *Haloferax* ortholog HVO_0586. This function assignment has to be considered tentative and awaits experimental confirmation. It should be noted that *Salmonella* CobC and CobS show reduced substrate specificity which allows them to function in arbitrary order. If CobC acts first, alpha-ribazole phosphatase is hydrolyzed to alpha-ribazole and then attached by CobS to the corrin ring. If CobS acts first, alpha-ribazole phosphate is attached to the corrin ring, resulting in adenosylcobalamin phosphate which is then hydrolyzed to adenosylcobalamin by CobC.

The other two reactions that need to be considered in more detail are involved in attachment of the aminopropanol arm. In *Salmonella*, L-threonine-O-3-phosphate decarboxylase (encoded by *cobD*, EC 4.1.1.81) generates the intermediate 1-aminopropan-2-yl phosphate, a substrate for CbiB [98]. For this enzyme, two paralogs with 51% protein sequence identity are encoded in close genomic vicinity in *Hfx. volcanii* (HVO_0591, cobD1, HVO_0593, cobD2). HVO_0591 shows 31% protein sequence identity to the characterized homolog from *Salmonella*. For HVO_0593, the assignment has to be based on its closely related paralog. A direct assignment based on a GSP is not possible, because BLASTp against SwissProt (accessed March 2021) returns histidinol-phosphate aminotransferases as top matches, but with less than 30% protein sequence identity. CobD decarboxylates threonine-O-3-phosphate, so that a threonine kinase is required. However, there is yet no candidate for this reaction (EC 2.7.1.177). Thus, either this reaction is catalyzed by a novel protein family or the corresponding pathway does not involve L-

threonine-O-3-phosphate and CobD1 and CobD2 catalyze a distinct (but probably related) reaction. Closing of this pathway gap has to await experimental analysis.

S3.d A potential late cobaltochelatase of the heterotrimeric type

Cobalt is inserted late in the aerobic biosynthesis pathway. A late cobaltochelatase has been characterized [99]. It is heterotrimeric (with genes assigned as *cobNST*) and was stated to originate from *Pseudomonas denitrificans* (reassigned to *Sinorhizobium sp.* by UniProt as of April 2021). Haloarchaea code for a homolog of CobN (e.g. *Hfx. volcanii* HVO_B0050). No homologs to the other two subunits (UniProt:P29929, subunit CobS, UniProt:P29934, subunit CobT) could be identified in haloarchaea.

Pseudomonas CobN is distantly related to subunit ChlH from the characterized heterotrimeric magnesium chelatase from *Synechocystis sp.* PCC 6803 [100,101]. The other subunits of the magnesium chelatase (ChlD and ChlI) are related to HVO_B0051. The N-terminal part of HVO_B0051 shows 46% protein sequence identity to ChlI and ca 30% protein sequence identity to the N-terminal part of ChlD. The remainder of HVO_B0051 shows ca 30% protein sequence identity to the remainder of ChlD. Comparative genomics analyses led to the observation that in several phylogenetic branches of bacteria the late cobaltochelatase has ChlID subunits instead of CobST subunits [96].

In summary, HVO_B0050 and HVO_B0051, which are encoded by adjacent genes within the major cobalamin cluster, might represent a late cobalt chelatase.

It may be unexpected to find a late cobalt chelatase in an organism that uses the cobalt-early biosynthetic pathway. However, a late cobalt chelatase may have a function in a corrin ring salvage pathway, a speculation not yet substantiated by experiment.

S3.e Two siroheme decarboxylase paralogs, each being a two-domain protein

The enzymes of the alternative heme biosynthesis pathway and their associated GSPs are listed in Table 3 (starting from siroheme and along the biosynthetic pathway; enzymes between precorrin-2 and siroheme are already covered above, see S3.a, *cbiX* gene paralogs). With respect

to the conversion of siroheme to 12,18-didecarboxysiroheme, this function has been attributed to two haloarchaeal proteins (encoded by *ahbA* and *ahbB*). Both are two-domain proteins, while several characterized homologs are single-domain proteins forming heterodimers. These catalyze both decarboxylations [102]. *Hydrogenobacter thermophilus* contains a single two-domain protein which has been characterized [103]. It is unclear if haloarchaeal AhbA and AhbB act independently (each forming a domain dimer) or as heterodimer (forming a domain tetramer).

S3.f Two distinct enzymes catalyze the last step of heme biosynthesis

The final two steps of the alternative heme biosynthesis pathway (AHB pathway), which is used by haloarchaea, are catalyzed by the products encoded of *ahbC* and *ahbD*, both being radical SAM enzymes. According to OrthoDB analysis, these enzymes are encoded in about two-thirds of the haloarchaeal genomes (107/118 of 166). AhbD catalyzes the decarboxylation of Fe-coproporphyrin III to protoheme (heme b) (adenosylmethionine-dependent heme synthase, EC 1.3.98.6). Many bacteria use the CPD pathway and not the classical heme biosynthesis pathway. The last step also is the same as in the AHB pathway, the decarboxylation of Fe-coproporphyrin III to protoheme [83]. However, a different type of enzyme is used for this step (encoded by *chdC*, peroxide-dependent heme synthase, EC 1.3.98.5) [83]. A ChdC homolog occurs in haloarchaea, resembling a specific domain fusion [104], and being encoded in nearly all haloarchaeal genomes (161 of 166). The protein from *Hfx. volcanii* was shown to catalyze the decarboxylation of Fe-coproporphyrin III [105]. It was found that AhbD is used under anaerobic conditions, while ChbD is used under aerobic conditions [105]. It is unresolved if and how haloarchaea without an *ahbC* gene can produce heme.

Section S4: Coenzymes II: coenzyme F420

S4.a Coenzyme F420 biosynthesis in haloarchaea

Coenzyme F420 was first detected in *Methanobacterium* [106] and is predominant in methanogenic archaea [107,108] but occurs also in certain bacteria and in small amounts in haloarchaea [109,110].

The pathway which creates the carbon backbone of coenzyme F420 has been reconstructed, based on the pathway characterized in *M. jannaschii*, and we list the enzymes with their

associated GSPs in Table 4. Coenzyme F420 contains a phospholactate moiety which was reported to originate from 2-phospho-lactate [111]. This compound is not well connected to the remainder of metabolism, and the assumed enzymes for its production could never be identified. The archaeal enzyme CofC (e.g. MJ0887) is involved in activation of 2-phospho-lactate and the enzyme CofD (e.g. MJ1256) in its attachment to the preassembled intermediate F_0 [111,112]. *Mycobacterium tuberculosis*, which also contains coenzyme F420, was assumed to also use this pathway. It turned out that the precursor for the phospholactate moiety was phosphoenolpyruvate, a common metabolic intermediate, rather than the atypical 2-phospho-lactate [113]. Phosphoenolpyruvate was activated (by FbiD, the homolog of CofC) and coupled (by FbiA, the homolog of CofD), leading to a new intermediate, dehydro-F420. The authors confirmed usage of phosphoenolpyruvate and generation of the novel intermediate also for the enzymes from *M. jannaschii* [113]. As a consequence, an additional enzyme activity is required, which reduces dehydro-F420 to F420. The authors found this activity in FbiB, a two-domain protein. The N-terminal part is homologous of CofE (MJ0768), the enzyme which attaches the polyglutamate tail to F420. The bacterial FbiB has a C-terminal extra domain which was known to contain a bound FMN for enigmatic reasons. It could be shown that this C-terminal domain reduces dehydro-F420 to F420, and that this reduction is a prerequisite for the N-terminal domain to attach the polyglutamate tail. Because the *Methanocaldococcus* CofE lacks such a domain, while CofC and CofD generate dehydro-F420, the authors tried to identify a genomically coupled dehydrogenase, but without success. The next twist to this story came with the analysis of coenzyme F420 biosynthesis in *Paraburkholderia rhizoxinica* [114]. This species uses 3-phospho-D-glycerate instead of phosphoenolpyruvate or 2-phospho-lactate and generates a F420 derivative which has a phosphoglyceryl moiety instead of a phospholactyl moiety [114]. These authors also determined the substrate specificity of the *M. jannaschii* enzymes CofC and CofD. In their hands, the enzymes work with the originally proposed precursor 2-phospho-lactate rather than the subsequently proposed precursor phosphoenolpyruvate. That would, again, resolve the problem with the missing dehydrogenase which converts dehydro-F420 to F420, but the problem that no biosynthetic route is known which leads to 2-phospho-lactate remains. It might thus be informative to subject the *Haloferax* coenzyme F420 biosynthetic pathway to experimental analysis.

S4.b Putative coenzyme F420-dependent oxidoreductases

To the best of our knowledge, only a single coenzyme F420 dependent enzymatic reaction has yet been reported for halophilic archaea [115]. No data were provided which would allow a gene assignment for this function. Thus, the importance of this coenzyme in haloarchaeal biology is highly enigmatic.

However, based on detailed bioinformatic analyses using a strategy called SIMBAL, coenzyme F420 dependent reactions have been predicted for bacteria [116]. Based on corresponding patterns and domains, F420 dependent enzymes were also predicted for haloarchaea.

HVO_0433 is a F420H2:NADP oxidoreductase, based on a characterized homolog from *A. fulgidus* [117].

HVO_B0113 is annotated as a probable F420-dependent oxidoreductase, which is supported by the assignment of a corresponding InterPro domain (IPR019952). Proteins with this domain assignment belong to a coenzyme F420-specific subset of proteins with a luciferase-like monooxygenase domain. HVO_B0113 is located within the rhamnose cluster but is not required for growth on rhamnose [118].

HVO_B0342 shows 29% protein sequence identity to a F420-dependent alcohol dehydrogenase from *Methanoculleus thermophilicus* which has been characterized, including a 3D structure with coenzyme F420 included as ligand [119,120].

HVO_1937 is a probable 5,10-methylenetetrahydrofolate reductase, an enzyme of one-carbon metabolism (see below, one-carbon metabolism). It is briefly listed here because it has coenzyme F420-dependent methanogenic homologs. It should be noted that HVO_1937 is encoded adjacent to the gene coding for one of the coenzyme biosynthesis proteins (CofE).

The *Nmn. pharaonis* protein NP_1902A is name-giving to the InterPro version of TIGR04024 (F420_NP1902A, IPR023909). TIGRFAM entries for coenzyme F420-dependent enzymes were generated during a bioinformatic study of F420-dependent enzymes in *M. tuberculosis*, applying

the SIMBAL algorithm [116]. NP_1902A or any of its close homologs has not been experimentally characterized, so that usage of F420 has not yet been confirmed. There is a paralog in *Nmn. pharaonis* (NP_4374A, 44% protein sequence identity) and there are more closely related orthologs (at least 60% protein sequence identity) in several haloarchaea (e.g. *Har. marismortui*, *Hbt. salinarum, Nab. magadii*). A close ortholog is absent from *Hfx. volcanii*, which only has distant homologs (e.g. HVO_A0572, 33% protein sequence identity, and no assignment of the F420_NP1902A domain by InterPro).

The *Nmn. pharaonis* protein NP_4006A is distantly related to Coenzyme F420-dependent sulfite reductase from *M. jannaschii* [121] and has the corresponding InterPro domain assigned (IPR007516).

In addition, there are proteins annotated as F420-dependent NADP oxidoreductase family protein (e.g. HVO_A0605). They belong to the same protein family as F420H2:NADP oxidoreductase, but only a subset uses coenzyme F420 while the others use NAD. Only experimental analysis can clarify the coenzyme specificity.

S4.c The coenzyme F420 precursor may be involved in DNA repair

UV light induces two types of DNA lesions in halophilic archaea: cyclobutane pyrimidine dimers and pyrimidine (6-4) pyrimidone photoproducts (for reviews on DNA repair see [122,123]). Photoreactivation of both lesion types has been detected, in addition to dark repair [124]. The action spectrum of photoreactivation has been analyzed in vivo and in cell extracts of *Hbt. salinarum*. The process depends on 8-hydroxy-5-deazaflavin (F0, the precursor of F420 lacking the poly-Glu tail) [125,126]. This is, however, an assignment to an overall process and not to individual proteins.

The enzyme which repairs cyclobutane pyrimidine dimers has been characterized in *Hbt. salinarum* (VNG_1335G, encoded by *phr2*) [127,128]. However, the chromophore has not been analyzed. Phr2 is one key player for the remarkable UV-radiation resistance of *Hbt. salinarum* [129]. The ortholog from *Hfx. volcanii* is HVO_2911.

Hfx. volcanii codes for two other potential photo-lyases (HVO_2843, encoded by *phr1*, HVO_1234, encoded by *phr3*). HVO_2843 belongs to the cryptochrome DASH class which was long considered not to be related to DNA repair [130,131]. However, it turned out that this class of proteins mediates photorepair of cyclobutane pyrimidine dimers in single-stranded DNA [132]. HVO_1234 may function as pyrimidine (6-4) pyrimidone photolyase [133].

Section S5: Coenzymes III: coenzymes of C1 metabolism: tetrahydrofolate in haloarchaea, methanopterin in methanogens

Several coenzymes are prominently associated with methanogenic organisms (methanopterin, coenzyme F420, coenzyme M, coenzyme B, cofactor F430). For two of these (methanopterin, coenzyme F420), corresponding annotations are found for halophilic archaea in public databases. As detailed above, this is valid for coenzyme F420. However, methanopterin, which is the one-carbon carrier in methanogenic archaea, does not exist in halophilic archaea, which use tetrahydrofolate as one-carbon carrier [134]. The distinct one-carbon carriers share a near-identical core structure (a pterin heterocyclic ring is linked via a methylene bridge to a phenyl ring) and also share a polyglutamate tail. The sequence similarity between methanopterin-specific methanogenic enzymes and tetrahydrofolate-specific haloarchaeal enzymes indicates divergence from a common ancestor. Experimentally characterized methanogenic enzymes thus may trigger annotation of haloarchaeal enzymes as being methanopterin-specific, which as a consequence implies the presence of methanopterin in haloarchaea. Such annotations are found in UniProt and KEGG (accessed April 2021). However, when haloarchaea switched from methanopterin to tetrahydrofolate as a C1 carrier, the haloarchaeal enzymes had to adapt to tetrahydrofolate, which shows structural similarity to methanopterin. Additionally, some details of haloarchaeal tetrahydrofolate biosynthesis are yet unresolved. A detailed review on the many variants of the tetrahydrofolate biosynthetic pathway is available [135]. Also, tetrahydrofolate and tetrahydromethanopterin have been compared [136].

S5.a A proposed pathway for aminobenzoate biosynthesis in haloarchaea

Tetrahydrofolate biosynthesis requires aminobenzoate. No haloarchaeal enzymes for the biosynthesis of aminobenzoate are assigned in KEGG (accessed April 2021). We had proposed

candidates for this pathway [1,137] which would convert chorismate to para-aminobenzoate. Our proposal is based on clustering of three genes, the products of which are homologous to enyzmes involved in amino acid biosynthesis. The components of a proposed aminodeoxychorismate synthase (HVO_0709 and HVO_0710, encoded by *pabAB*) are homologous to those of anthranilate synthase (encoded by *trpEG*). A proposed aminodeoxychorismate lyase (encoded by *pabC*) is distantly related to the product of *ilvE* (branched-chain amino acid aminotransferase). According to SyntTax analysis, *pabAB* are strictly clustered and *pabC* is genomically coupled in the majority of haloarchaeal genomes. This prediction is not supported by sufficient evidence for databases to adopt this. Also, the detailed review on tetrahydrofolate biosynthesis states that “in Archaea, the pABA synthesis pathway remains a mystery” [135].Thus, this prediction is awaiting experimental support or disproval.

S5.b GTP cyclohydrolase MptA is probably involved in biosynthesis of tetrahydrofolate rather than methanopterin

The annotation of HVO_2348 (GTP cyclohydrolase MptA, encoded by *mptA*, with gene synonym *folE2*) is based on the characterized homolog from *M. jannaschii* [138]. In UniProt, the protein from this methanogenic archaeon, is stated to "convert GTP to 7,8-dihydro-D-neopterin 2',3'-cyclic phosphate, the first intermediate in the biosynthesis of coenzyme methanopterin". Unfortunately, this statement is copied to HVO_2348 (as of March 2021), which is invalid, given that haloarchaea do not generate/contain methanopterin. The initial reactions of methanopterin and of tetrahydrofolate biosynthesis are common (up to the intermediate (7,8-dihydropterin-6-yl)methyl diphosphate). However, the enzymes specific for methanopterin biosynthesis are absent from haloarchaea. It should be noted that HVO_2348 has been partially characterized [139]. Deletion mutants of HVO_2348 are auxotrophic for thymidine and hypoxanthine. The conversion of dUMP to dTMP is dependent on dihydrofolate. Two enzymatic reactions of de novo purine biosynthesis (EC 2.1.2.2 and 2.1.2.3) are also dependent on tetrahydrofolate, and this block can be overcome by purine salvage, using hypoxanthine.

The product of HVO_2348 is the substrate for EC 3.1.4.56. This enzyme has been characterized from *M. jannaschii* (MJ0837) but remains a pathway gap in halophilic archaea. MJ0837 is very distantly related to HVO_A0533 with only 27% protein sequence identity. Nevertheless,

HVO_A0533 might be a promising candidate and a deletion strain could be checked for thymidine and hypoxanthine auxotrophy.

HVO_2628 is a member of the GHMP kinase family (kinases for galactose, homoserine, mevalonate and phosphomevalonate). There is about 30% protein sequence identity to beta-ribofuranosylaminobenzene 5'-phosphate synthase (EC 2.4.2.54) and this function is also assigned to HVO_2628 by UniProt and by KEGG (as of March 2021). This is based on the enzymatically characterized and distantly related enzymes from *A. fulgidus* [140] and *M. jannaschii* [141]. The reaction of EC 2.4.2.54 is not a simple kinase reaction, and it represents the first committed step to methanopterin biosynthesis. As this coenzyme is absent from halophilic archaea, a distinct function is likely. However, no corresponding reaction is required in the tetrahydrofolate biosynthesis pathway. The true biological function of HVO_2628 remains unresolved.

S5.c Enzymes which alter the oxidation level of the coenzyme-attached one-carbon compound likely operate on tetrahydrofolate rather than methanopterin

HVO_2573 (*mch*, probable methenyltetrahydrofolate cyclohydrolase; EC 3.5.4.9) is annotated in UniProt and KEGG as "methenyltetrahydromethanopterin cyclohydrolase (EC 3.5.4.27)" (as of March 2021). The characterized homolog is MK0625 from the methanogenic archaeon *M. kandleri* [142]. These proteins show 45% protein sequence identity. The C1 compound is attached to the substructure shared between tetrahydrofolate and methanopterin, and it is quite possible that these homologous proteins catalyze an equivalent reaction, but using distinct coenzymes. An experimental characterization of the haloarchaeal protein would unequivocally resolve this annotation ambiguity.

HVO_1937 (*mer*, probable 5,10-methylenetetrahydrofolate reductase) is annotated in UniProt and KEGG as "5,10-methylenetetrahydromethanopterin reductase (EC 1.5.98.2)" (as of March 2021). This annotation is based on the characterized *mer* from the methanogenic archaeon *M. thermoautotrophicus* [143-145]. These proteins show 38% protein sequence identity. As stated for HVO_2573, the usage of distinct but structurally related coenzymes is well possible at this level of sequence conservation. As mentioned above (see section S4.b), the methanogenic

enzyme functions with coenzyme F420 and this is also likely for the halophilic protein. Again, experimental characterization would resolve these issues.

Section S6: Coenzymes IV: NAD and FAD (riboflavin)

S6.a Two NAD kinase paralogs may function with ATP or polyphosphate

The paralogs *nadK1* (HVO_2363) and *nadK2* (HVO_0837) are only distantly related to each other. HVO_2363 is more closely related to NAD kinase from *M. tuberculosis* than to NAD kinase from *A. fulgidus*. The *Mycobacterium* enzyme can use both, ATP and pyrophosphate as energy source [146]. This information seems unavailable for the enzyme from *A. fulgidus*, even though the protein has been crystallized together with NAD, NADP, and ATP [147]. After soaking with ATP, the structure contains two ATP molecules. One is well structured but occupies part of the NAD binding site, which thus may represent fortuitous binding in the absence of NAD. For the other, which is considered to represent the phosphate donor, only the beta/gamma pyrophosphate is ordered enough to be visible. This might imply that this NAD kinase may function with both, ATP and polyphosphate. HVO_0837 is even more distantly related to characterized homologs. Thus, the assignment of the energy source of the *Haloferax* enzymes is not possible in the absence of experimental data. It should, however, be noted that polyphosphate could not be identified in exponentially growing *Hfx. volcanii* cells by DAPI staining [148], so that ATP is the more likely energy source.

S6.b HVO_0781 may be involved in NAD biosynthesis due to a highly conserved gene neighbourhood with HVO_0782 (*nadM*)

HVO_0782 is annotated as nicotinamide-nucleotide adenylyltransferase (*nadM*, EC 2.7.7.1), a step in NAD biosynthesis, based on the characterized homolog from *M. jannaschii* [149,150]. This enzyme is present in all Halobacteria (163 species) and even in 399 of 405 archaea (according to OrthoDB analysis, March 2021). The gene neighbourhood to its adjacent gene, HVO_0781, is conserved in most haloarchaea and beyond according to SyntTax analysis. HVO_0781 occurs in nearly all halophilic archaea (158 of 166) and in 296 out of 405 archaea, indicating that it must have an important function. It is related to the characterized S-adenosyl-L-methionine hydrolase (adenosine-forming) from *Salinispora arenicola* and from *Pyrococcus horikoshii* [151,152]. S-adenosyl-L-methionine hydrolase has the function to destroy S-

adenosylmethionine, which seems a wasteful reaction, and not expected to have been very well conserved over evolutionary time. Currently, HVO_0781 is annotated as "S-adenosylmethionine hydroxide adenosyltransferase family protein" to avoid the firm assignment of a function that is considered metabolically useless. The very strongly conserved gene neighbourhood with the NAD biosynthesis enzyme NadM may hint to an involvement in NAD biosynthesis, which could be investigated by generating mutants and checking for potential phenotypes.

S6.c Candidate genes which fill various gaps in riboflavin biosynthesis

Archaeal organisms were included in a 2017 study that provided an extensive bioinformatic analysis of riboflavin biosynthesis [153]. Reconstruction of this pathway for haloarchaea, including enzymes and their GSPs, is provided in Table 6. In riboflavin biosynthesis, D-ribulose-5-phosphate is converted by RibB (HVO_0327) to a compound termed “VI” [153]. This is condensed with another compound (termed "V’") by RibH (HVO_0974, 6,7-dimethyl-8-ribityllumazine synthase, EC 2.5.1.78). Compound "V’" is generated by the successive actions of ArfA, ArfB, ArfC, a gene product referred to as PyrD, and a putative phosphatase [153]. Only two of these enzymes have sufficient support to be annotated (ArfA, HVO_1284, EC 3.5.4.29, ArfC, HVO_1341, EC 1.1.1.302). For ArfB (EC 3.5.1.102), HVO_1235 has been proposed as a candidate [153]. For the so-called PyrD (RIB2 in KEGG as of April 2021, similar to but distinct from EC 3.5.4.26), HVO_2483 was proposed as a candidate [153]. If confirmed, a distinct gene name would have to be assigned (*pyrD* is already assigned to dihydroorotate dehydrogenase). No proposal has been made for the phosphatase which has now been assigned in various species (according to the information associated with EC 3.1.3.104) [154-156] but without enabling a prediction for haloarchaea.

The conversion of riboflavin to FMN is catalyzed by a bifunctional CTP-dependent riboflavin kinase / transcription regulator (HVO_0326, *rbkR*) which contains a N-terminal HTH domain and a C-terminal catalytic domain. FMN is then converted to FAD by HVO_1015 (encoded by *ribL*). The enzymes proposed to close the pathway gaps (ArfB and pyrimidine deaminase) require experimental confirmation.

Section S7: Biosynthesis of membrane lipids, bacterioruberin and menaquinone

S7.a Geranylgeranyl diphosphate synthase and geranylfarnesyl diphosphate synthase

The main core lipid of haloarchaea is archaeol, which consists of glycerol with ether-linked C20 isoprenoid units, which are derived from geranylgeranyl diphosphate. In *Hfx. volcanii*, HVO_2725 (*idsA1*) and HVO_0303 (*idsA2*) are annotated as geranylgeranyl diphosphate synthase.

Besides the main core lipid archaeol, there are variants, some of which have a C25 isoprenoid rather than a C20 isoprenoids (see e.g. [157,158]. These longer isoprenoids commonly occur in alkaliphilic organisms like *Nmn. pharaonis* and *Nab. magadii* [159]. An enzyme involved in C25 isoprenoid biosynthesis (geranylfarnesyl diphosphate synthase) has been purified and characterized on the enzymatic level in *Nmn. pharaonis* [160]. However, data allowing gene assignment were not provided in that study, nor has it been cited in any later publication reporting gene assignment. Three candidate genes exist in *Nmn. pharaonis*, (NP_0604A, NP_3696A, and NP_4556A). As NP_0604A, NP_3696A have orthologs in *Hfx. volcanii*, an organism devoid of C25 lipids, and as orthologs of these are found in nearly all genomes from Halobacteria according to OrthoDB analysis (159/162 of 166), we assign geranylfarnesyl diphosphate synthase activity to the third paralog, NP_4556A. This protein has a close homolog in comparably few halophilic archaea according to OrthoDB (64 of 166), among them *Nab. magadii*, which is also alkaliphilic. Uniprot added the Tachibana 1994 reference [160] to the entry for NP_3696A and assigned geranylfarnesyl diphosphate synthase activity to this paralog (as of April 2021) but without providing any evidence for this assignment. Geranylfarnesyl diphosphate synthase has been characterized in *A. pernix* (*fgs*, APE_1764) [161], but for this class of enzymes general sequence similarity is insufficient to predict substrate specificity. Our assignments are supported by analysis of key residues which determine the length of the isoprenoid chain [162]. These authors label the cluster containing NP_3696A (WP011323557.1) as “C15/C20” and the cluster containing NP_4556A (WP011323984.1) as “C20->C25->C30?”. Unambiguous specificity assignments have to await experimental characterization.

S7.b Biosynthesis of the phospholipid headgroups is only partially resolved

The haloarchaeal core lipid, archaeol, is activated to CDP-archaeol by the product of the *carS* gene (HVO_0332). Distinct headgroups are then attached by CDP-archaeol 1-archaetidyltransferases which share a common domain (represented e.g. by InterPro:IPR000462). Four members of this protein family are encoded in the *Hfx. volcanii genome* (HVO_1136, HVO_1143, HVO_1297, HVO_1971). Even though these proteins share the same domain, they are so distant that they do not detect each other upon BLASTp analysis (stringency of 10^{-5}).

HVO_1143 is distantly related (32% protein sequence identity) to a functionally characterized archaetidylserine synthase from *M. thermoautotrophicus* (MTH_1027) [163]. The *Hbt. salinarum* ortholog (VNG_0784G) has been assigned the same function in a bioinformatic analysis [164]. HVO_1143 has recently been shown to be involved in ArtA-dependent protein maturation, a process which includes C-terminal proteolytic cleavage and lipid attachment [165]. It is referred to as PssA, based on the gene assigned to the bacterial phosphatidylserine synthase. HVO_0146, which probably decarboxylates archaetidylserine to archaetidylethanolamine, was also found to be involved in ArtA-dependent protein maturation (referred to as PssD) [165]. The same function was also proposed for the *Hbt. salinarum* ortholog (VNG_2255C) [164].

The substrate assignment to HVO_1297 is uncertain. It shows distant similarity (25% protein sequence identity) to the characterized archaetidylinositol phosphate synthase from *M. thermoautotrophicus* (MTH_1691, EC 2.7.8.39) [166]. The ortholog from *Nmn. pharaonis* (NP_2144A) is somewhat less distant, having 32% protein sequence identity. KEGG also assigns EC 2.7.8.39 (via KO group K17884, accessed April 2021). It should be noted that MTH_1691 does not retrieve any of the other CDP-archaeol 1-archaetidyltransferases from *Hfx. volcanii* upon BLASTp analysis (stringency of 10^{-5}). Upon bioinformatic analysis, the *Hbt. salinarum* ortholog (VNG_1030G) had been assigned to function as archaetidylglycerol synthase [164]. This assignment is exclusively based on the reported absence of archaetidylinositol from haloarchaea [164,167]. In a subsequent review, the corresponding cluster is stated to be “related to myo-inositol-phosphate transfer” [168] and the authors add “(and maybe also glycerol in archaea, according to classification in [70]”, which refers to [164].

Various issues need to be taken into account for the evaluation of this specific open issue. (a) Is archaetidylinositol really absent from haloarchaea, even as a pathway intermediate? In this context it should be noted that archaetidylethanolamine has long been considered absent from haloarchaea, but recently has been identified (see above). (b) It should be taken into account which fraction of the haloarchaeal genomes codes for an ortholog of the *Haloferax* proteins. According to OrthoDB, orthologs of HVO_1143 and HVO_1297 are encoded in nearly all of the genomes from *Halobacteria* (159/161 of 166), of which only about half (80 out of 166) code for an ortholog of HVO_1136 and only one-fifth (26 out of 166) code for an ortholog of HVO_1971. As PGP and PGP-Me are prominent phospholipids in most haloarchaea, this might imply that HVO_1297 is more likely involved in attachment of glycerol phosphate than myo-inositol phosphate. (c) HVO_1297 is part of a three-gene operon, the synteny of which is highly conserved according to SyntTax analysis. We thus evaluated if the adjacent genes might shed light on the function prediction for HVO_1297.

One gene of the cluster is annotated as histidinol-phosphate aminotransferase (HVO_1295, encoded by *hisC*). This function assignment is based on the capability of this gene to overcome a histidine auxotrophy mutation [169]. The complementing gene was then found to be distantly related (31% protein sequence identity) to *E. coli* HisC. Substrate assignments are highly uncertain at this level of sequence identity in this protein family (see above, Section 2). It should be kept in mind that the complementation activity observed for HVO_1295 might be due to a side reaction of an enzyme with relaxed substrate specificity and that the major function of this pyridoxal-phosphate dependent aminotransferase might be involved in a polar lipid biosynthesis pathway.

The other gene of this highly conserved three-gene operon is annotated as adenylate kinase (HVO_1296, encoded by *adk2*). There is comparably low sequence identity to proteins with confirmed adenylate kinase activity. HVO_1296 shows 34% protein sequence identity to a *Pyrococcus* homolog, which seems to have its primary function in ribosome biogenesis [170]. HVO_1296 shows 32% protein sequence identity to human AK6 which has broad substrate specificity and is more a nucleotide kinase rather than an adenylate kinase [171]. It should be

noted that the *adk1* gene product of *Haloferax* (HVO_2496) has a much stronger support as being adenylate kinase (45% protein sequence identity to a characterized homolog from *B. subtilis*). Thus, there is only weak support for HVO_1296 being an adenylate kinase and the protein might, instead, function as inositol kinase. In that case, the reaction product, myo-inositol-1-phosphate, would be a potential substrate for HVO_1297 if that enzyme would generate archaetidylinositol phosphate.

Haloferax codes for an additional gene that is annotated as myo-inositol-1-phosphate synthase, HVO_B0213. Assignment of this specificity is based on a characterized homolog from *A. fulgidus* (AF_1794). Only few haloarchaea code for a close homolog (32 of 166). If a majority of haloarchaea would produce archaetidylinositol, at least as a pathway intermediate, a more widely distributed enzyme would be required. In that case, HVO_1296 (as inositol kinase) and HVO_1297 (as archaetidylinositol phosphate synthase) might be responsible for these activities. In summary, the substrate of HVO_1297 remains uncertain and will stay an open issue until being resolved by experimental analysis.

According to OrthoDB analysis, the two residual members of the CDP-archaeol 1-archaetidyltransferase family are encoded in about half (80 out of 166 for HVO_1136) and about one-fifth (26 out of 166 for HVO_1971) of the haloarchaea. HVO_1136 does not show full-length similarity to a GSP, so that this approach does not allow a specific function assignment. Speculations regarding the function of HVO_1136 depend on the tentative function assignment to HVO_1297. If HVO_1297 were an archaetidylinositol phosphate synthase, then HVO_1136, having a wider representation in haloarchaea, would be a candidate for an archaetidylglycerophosphate synthase. The product of this enzyme is PGP, which is probably methylated to PGP-Me, a polar lipid found in a wide variety of haloarchaea. On the other hand, PGP is dephosphorylated to PG, which also is found in many haloarchaea. Support for this assignment might be the adjacent gene (HVO_1135), which is encoded on the opposite strand. Based on an InterPro domain assignment, it is probably a S-adenosylmethionine-dependent methyltransferase. HVO_1971, being comparably rare in haloarchaea, shows 26% protein sequence identity to the characterized archaetidylserine synthase from *M. thermoautotrophicus*

(MTH_1027) which, however, is more closely related to HVO_1143. Thus, we think that no specific function can be assigned for HVO_1971. All proposed function assignments have to be considered uncertain until their experimental confirmation or rejection.

Haloarchaea are also known to contain cardiolipin and glycolipids (e.g. S-DGD-1, sulfated diglycosyldiether) [172]. The biosynthetic pathways leading to these polar lipids are currently enigmatic. Also enigmatic are the enzyme responsible for sulfation of the glycolipid, and the enzyme responsible for the sulfur transfer reaction that leads to PGS.

S7.c Conversion of geranylgeranyl diphosphate to the carotenoid bacterioruberin

The C20 compound geranylgeranyl diphosphate is converted to the C40 carotenoid phytoene by head-to-head condensation. This reaction is catalyzed by phytoene synthase (encoded by *crtB*, e.g. HVO_2524). Only distantly related homologs have been enzymatically characterized [173]. However, *crtB* mutants are colorless [77,174,175], which supports the involvement of this gene in carotenoid biosynthesis.

Next, phytoene is reduced to lycopene by phytoene desaturase. The bioinformatic assignment of phytoene desaturase activity to a gene proved to be difficult and will be covered after bacterioruberin biosynthesis.

Lycopene is converted to bacterioruberin by three enzymes which have been characterized experimentally in *Haloarcula japonica* [176]. The genes for these enzymes form a three gene cluster which is syntenically highly conserved according to SyntTax analysis. Based on the strict syntenic conservation of this three gene cluster in haloarchaea, we hypothesize that all orthologs are isofunctional to the enzymes from *Har. japonica*. Lycopene elongase (encoded by *lyeJ*, c0506 in *Har. japonica*, HVO_2527 in *Hfx. volcanii*, NP_4766A in *Nmn. pharaonis*) adds a C5 isoprenoid unit to one end of lycopene, generating a C45 intermediate [176]. LyeJ is bifunctional and also acts as a 1,2-hydratase. Next, carotenoid 3,4 desaturase (encoded by *crtD*, c0506, HVO_2528, NP_4764A) introduces a double bond in the newly attached isoprenoid unit. This

pair of reactions is repeated on the other side of the molecule, thus resulting in a C50 compound. Addition of C5 isoprenoid units to one end of lycopene was first observed in *Hbt. salinarum* [176,177]. As last step in biosynthesis, a C50 carotenoid 2”-3”-hydratase (encoded by *cruF*, c0505, HVO_2526, NP_4768A) generates the final product bacterioruberin.

This leaves one pathway gap, phytoene desaturase. For *Nmn. pharaonis*, this reaction had initially been attributed to NP_4764A (encoded by *crtI1*, now known as *crtD*, see above) and NP_0204A, encoded by *crtI2* [137]. It is uncertain and may even be considered unlikely that the *crtD* product (c0506, HVO_2528, NP_4764A) has broad substrate specificity and also has phytoene desaturase activity, thus acting on a C40 compound and on bonds which occur in the center of the molecule. Deletion of the *crtD* gene in *Har. japonica* (c0507) did not interfere with lycopene biosynthesis [176]. In *Hfx. volcanii*, a deletion mutant of *crtD* was white in colour [174], which might indicate that lycopene, a red-colored compound, cannot be produced. The *Nmn. pharaonis* paralog of *crtD*, NP_0204A, which has 60% protein sequence identity to NP_4764A, has comparably few orthologs in haloarchaea and thus may not represent a key enzyme of lycopene biosynthesis. Most haloarchaea have additional very distant paralogs of *crtD* (in *Nmn. pharaonis*/*Hfx. volcanii* NP_1630A/HVO_0817 and NP_4520A/HVO_2340). Despite being very distantly related, these are obvious candidates for being a phytoene desaturase. The *Hfx. mediterranei* ortholog of HVO_0817 (HFX_0786, HFX_RS03805) is depicted as a candidate for phytoene desaturase activity in the detailed bioinformatic reconstruction of haloarchaeal carotenogenesis (see Fig.3 of [178]), even though *Hfx. volcanii* HVO_0817, HVO_RS08620, is not depicted in that figure. It should be noted that phytoene desaturase catalyzes an oxidoreductase reaction, and the next gene upstream of the *crtD-lyeJ-cruF* three gene cluster codes for an oxidoreductase of the short-chain dehydrogenase family (HVO_2529, an ortholog of NP_4762A).

S7.d Reconstruction of menaquinone biosynthesis

Halophilic archaea contain high levels of menaquinone (mainly MK8:8) [179,180]. The pathway for menaquinone biosynthesis has been reconstructed, but two pathway gaps remain open. The enzymes and their associated GSPs are listed in Table 7 (starting from chorismate and along the

biosynthetic pathway). Many functions can be attributed by clear, though distant, sequence similarity (enzymes encoded by *menF*, *menD*, *menE*, *menB*, *menA*, *menG*). In one case, sequence similarity is very low but the assignment is supported by clustering of many genes coding for menaquinone biosynthesis enzymes (*menC*, HVO_1461). This assignment, and also that of menG has not been made by KEGG (accessed March 2021). Two pathway gaps remain (MenH, EC 4.2.99.20 and MenI, EC 3.1.2.28). There are five genes with unassigned function in the gene cluster coding for enzymes of the menaquinone biosynthesis pathway (HVO_1463,1464,1466,1467,1468), but we could not detect any obvious relationship between the domains assigned to their products and the missing enzymatic reactions.

Section S8: Issues concerning RNA polymerase, protein translation components and signal peptide degradation

S8.a RNA polymerase subunit epsilon (*rpoeps*)

Beyond the canonical haloarchaeal RNA polymerase subunits (encoded by *rpoA1A2B1B2DEFHKLNP*), *Hbt. salinarum* was found to contain an additional subunit called epsilon [181,182]. This was required for efficient translation of native templates [182]. Even though the subunit is encoded in the genome of *Halococcus morrhuae* (C448_10607), it was not identified in the purified RNA polymerase from that species [183]. While canonical RNA polymerase subunits are found in nearly all haloarchaea (e.g. 159 of 166 when OrthoDB is queried for subunit H), the epsilon subunit is only found in a subset of the Halobacteria (73 of 166). It occurs in most *Natrialbales* (47 of 49) and about half of the *Halobacteriales* (24 of 46) but is rare in *Haloferacales* (3 of 71). We consider it astounding that the core component of genetic information processing shows this level of variability within the haloarchaea. The biological relevance of this subunit seems largely unresolved.

S8.b Ribosomal proteins having two distant paralogs

Two distant paralogs are found for haloarchaeal ribosomal protein S10 in nearly all haloarchaeal genomes (in *Hfx. volcanii* HVO_0360, encoded by *rps10a*, and HVO_1392, encoded by *rps10b*, with 24% protein sequence identity). It is uncertain if both occur in the ribosome, together or alternatively, the latter would result in heterogeneity of the ribosomes. Alternatively, one of the

paralogs may exclusively have a non-ribosomal function. According to SyntTax analysis, *rps10a* and *tef1a1* form a gene pair that is highly conserved and is found in nearly all haloarchaea. The gene *tef1a1* codes for translation elongation factor aEF-1 alpha / peptide chain release factor aRF-3. This gene clustering supports a ribosomal function of the *rps10a* gene product. The gene *rps10b* is monocistronic but the divergently transcribed upstream gene (HVO_1391, NUDIX family hydrolase) is adjacent in most haloarchaeal genomes.

In a subset of archaea, two distant paralogs are found for haloarchaeal ribosomal protein S14 (in *Nmn. pharaonis* NP_4882A, encoded by *rps14a*, and NP_1768A, encoded by *rps14b*). The C-terminal ca 30 aa show 82% protein sequence identity while the N-terminal ca 20 aa are unrelated. The gene *rps14a* is encoded within a huge operon encoding 22 ribosomal proteins. The gene *rps14b* is monocistronic and its gene neighbourhood is not conserved.

S8.c A highly conserved C2-C2 type zinc finger is present in ribosomal protein L43e from *Halobacteriales* but absent in those from *Haloferacales* and *Natrialbales*

A subset of archaeal ribosomal proteins has homologs in eukaryotes but not in bacteria. Among these is ribosomal protein L43e, the homolog of the eukaryotic protein initially named L37a (rat) and later L43 (yeast). This protein is known to contain a zinc finger, representing a rubredoxin-like metal-binding fold according to InterPro (IPR011331) (CxxCx_nCxxC). For the yeast protein it was found that each of the four cysteine residues is required for zinc binding but only one of them is essential for the function of the protein [184]. Between the CxxC patterns is a conserved “Trp motif” (Rxxx[G/A/S][I/V/L]W) and mutation of the strictly conserved tryptophan residue is lethal [185]. Ribosomal protein L43e (L37a3) from *Har. marismortui* had not been sequenced prior to determination of the X-ray structure of the large ribosomal subunit at 2.4 A resolution [186] but the protein could be built into the structure, based on the homologous sequences from rat and yeast, which was facilitated by the bound zinc atom.

The strictly conserved residues of the zinc finger motif (CPxC and CxxCG) are reminiscent of those found in the so-called “small CPxCG-related zinc finger proteins” [187-189].

While the zinc finger and the central “Trp motif” are conserved in most homologs from euryarchaeota, there are exceptions. Both motifs are conserved in all euryarchaeal proteins from taxonomic orders outside Halobacteria, while within Halobacteria this is only the case for proteins of the order *Halobacteriales*. None of the proteins from the taxonomic order *Haloferacales* and a minority of the proteins from the taxonomic order *Natrialbales* contain the zinc finger, while the Trp-motif is conserved. This surprising observation was first made by UniProt staff (and communicated by e-mail) when their stringent consistency checking routines, applied to members of HAMAP MF_00327, failed to detect the expected cysteine residues [190].

Section S8.d Diphthamide, a posttranslational modification of translation elongation factor EF-2

Three enzymatic reactions convert a histidine residue in translation elongation factor EF-2 into diphthamide. We list these three enzymes in Table 8. Two of these (encoded by *dph2* and *dph5*) are annotated according to characterized homologs from *P. horikoshii* (PH1105, PH0725) [191,192]. The last enzymatic step (encoded by *dph6*) was assigned in a detailed bioinformatic analysis [193]. This assignment is consistent with distant similarity to the N-terminal region of a characterized homolog from yeast (YLR143W encoded by *DPH6*) [194,195].

S8.e Signal peptide peptidase

N-terminal signal sequences target proteins to the secretion machinery. Subsequent to membrane insertion or transmembrane transfer, the signal sequence is cleaved off by signal peptidase I (HVO_0002, HVO_2603) or signal peptidase II (still awaiting its identification in haloarchaea). After cleavage, the signal peptide must be degraded to avoid clogging of the membrane. Degradation is catalyzed by signal peptide peptidase. Candidates for this activity have been predicted from two protein families: the paralogs *sppA1* (HVO_0881) and *sppA2* (HVO_1987) are members of MEROPS family S49. A DUF1119 family protein (HVO_1107), which is a member of MEROPS family A22, has been predicted to function as signal peptide peptidase [196,197].

Section S9: Miscellaneous metabolic enzymes and proteins with other functions

S9.a A candidate gene for ketohexokinase

Ketohexokinase has been characterized in *Haloarcula vallismortis* [198]. Beyond enzymatic properties, only the amino acid composition has been reported. A speculative gene assignment was subsequently made [199] in *Halomicrobium mukohataei* (Hmuk_2662, the ortholog of HVO_1812). This protein belongs to a sugar kinase family and is in genomic vicinity with fructose 1-phosphate kinase (Hmuk_2661) and fructose bisphosphate aldolase (Hmuk_2663). Further bioinformatic analyses supported this assignment [200]. Homologs of Hmuk_2662 were found in six genomes, including species that are stated to have confirmed ketohexokinase activity (*Har. vallismortis*, *Har. marismortui*, *Hfx. mediterranei*). The amino acid composition of the homolog from *Har. vallismortis* (C437_04081) shows a close correlation with that of the characterized enzyme. Overall, bioinformatic analyses strongly support the assignment of ketohexokinase activity to Hmuk_2662 and C437_04081, with experimental confirmation still pending.

S9.b A candidate gene for fructokinase

The same paper which strongly supported the gene assignment to ketohexokinase [200] also assigned a gene to fructokinase. Differential proteomics in *Hht. litchfieldiae* grown in the presence and absence of sucrose showed an up-regulation of halTADL_1913 which, according to bioinformatic analyses, is a candidate for having fructokinase activity. A phylogenetic tree places halTADL_1913 into a cluster of 17 haloarchaeal genes. Among the most closely related sequences is a *Salmonella* gene product with 37% protein sequence identity that complements a fructose kinase deficiency (UniProt:P26984) [201] and OCC_03567 from *Thermococcus litoralis* (31% protein sequence identity) which has been experimentally analyzed [202]. Again, experimental confirmation may be possible by heterologous expression in *Hfx. volcanii* of halTADL_1913, OCC_03567, or another member of this sequence cluster.

S9.c A candidate gene for glucoamylase

There is a candidate ortholog set for having glucoamylase activity (*Hfx. volcanii* HVO_1711, *Haloquadratum walsbyi* HQ2539A, *Halorubrum sodomense* SAMN04487937_2677 [UniProt:A0A1I6HD35]). Glucoamylase from *Hrr. sodomense* (referred to in the older literature

as *Halobacterium sodomense*) has been characterized [203]. Beyond enzymatic characterization, only the amino acid composition has been reported. The correlation between the reported amino acid composition and the theoretical amino acid composition of UniProt:A0A1I6HD35 is only moderate ($R^2 = 0.72$). UniProt:A0A1I6HD35 shows 51% protein sequence identity to HVO_1711. The C-terminal half of HVO_1711 shows 33% protein sequence identity to glucoamylase from *Clostridium* strain G0005 which has been characterized [204]. Taken together, there is clear bioinformatic support for assignment of that function but due to the high level of uncertainty experimental validation seems necessary.

S9.d A candidate gene for glucose-6-phosphate isomerase

There is a strong candidate for having glucose-6-phosphate isomerase activity is *Hfx. volcanii* (HVO_1967, *pgi*). It is a homolog to *M. jannaschii* MJ1605 (36% protein sequence identity) which has been characterized [205].

S9.e A candidate gene for 2-dehydro-3-deoxy-(phospho)gluconate aldolase

A candidate gene product for 2-dehydro-3-deoxy-(phospho)gluconate aldolase activity is *Hbt. salinarum* KdgA (OE_1665R, *kdgA*). It shows 36% protein sequence identity to *Hfx. volcanii* HVO_1101 (encoded by *dapA*), which is involved in lysine biosynthesis. However, there are no other lysine biosynthesis genes in *Hbt. salinarum*. Instead, OE_1665R may be isofunctional with much more distantly related proteins, KdgA from *Saccharolobus (Sulfolobus) solfataricus* (SSO3197) and *Thermoproteus tenax* (TTX_1156.1) which have been characterized [206]. The function assignment to OE_1665R is shared by KEGG but not by UniProt (accessed March 2021). The assignment is supported by the adjacently encoded protein (OE_1664R) which catalyzes the preceding step in the pathway and another enzyme encoded in the vicinity (OE_1669F) which also codes for a step in the same pathway. Only few haloarchaea seem to have a close homolog of OE_1665R, including *Hbt. hubeiense*. This species, which codes for the enzymes of the lysine biosynthesis pathway, has two paralogs, one being close to OE_1665R (Hhub_1547, *kdgA*), the other close to HVO_1101 (Hhub_1591, *dapA*). OrthoDB places the paralogs from *Hbt. hubeiense* into the same group, so that the number of haloarchaeal genomes coding for an ortholog of OE_1665R cannot be easily retrieved. The function assignment to

OE_1665R and its close orthologs has to be considered uncertain until its experimental confirmation (or rejection).

S9.f A putative NAD-independent L-lactate dehydrogenase

LudBC (e.g. HVO_1692 and HVO_1693) is a probable NAD-independent L-lactate dehydrogenase which converts lactate into pyruvate. Lactate is one of the products of rhamnose catabolism, and in some haloarchaea *ludBC* gene pairs are found within the rhamnose catabolic cluster [118]. In *Hfx. volcanii*, the *ludBC* gene pair is not part of the rhamnose catabolic cluster, but deletion of *ludBC* (HVO_1692 and HVO_1693) impairs growth on rhamnose [118]. LudBC orthologs are found in many haloarchaea, with gene clustering being highly conserved. Both, *ludC* and *ludB* contain an assigned InterPro domain (LUD, previously DUF162, IPR003741).

A NAD-independent L-lactate dehydrogenase (*lutABC* in *B. subtilis* [207]; *lldABC* in *Pseudomonas stutzeri* [208]) has been characterized. LutC/LldC is very distantly related (up to 33% protein sequence identity, BLASTp E-value 10^{-8}) to homologs from *Hfx. volcanii* (HVO_1693, encoded by *ludC*) and *Nmn. pharaonis* (NP_1724A), which show 44% protein sequence identity to each other. LutB/LldB is related (up to 38% protein sequence identity) to the N-terminal part (up to ca pos 490 of 733) of the homologs from *Hfx. volcanii* (HVO_1692, encoded by *ludB*) and *Nmn. pharaonis* (NP_1722A) which show 60% protein sequence identity to each other.

Upon superficial analysis, it seems that *Hfx. volcanii* and *Nmn. pharaonis* lack homologs to *B. subtilis* LutA and *Pseudomonas* LldA. LutA and LldA show 42% protein sequence identity to each other and have a pair of IPR004017 domains assigned. LutA and LldA are distantly related to the C-terminal part of GlpC (e.g. HVO_1540, glycerol-3-phosphate dehydrogenase subunit C, 25% protein sequence identity, E-value 10^{-9}) and other FAD-dependent oxidoreductases. LldA but not LutA is very distantly related to the C-terminal part of LudB (27% protein sequence identity, E-value 10^{-5}). Neither *Hfx. volcanii* nor *Nmn. pharaonis* LudB have an IPR004017 domain assigned but have a C-terminal part with no other InterPro domain assignment. Thus, , haloarchaeal *ludB* may well represent a domain fusion (*lutBA*, *lldBA*). In that case, HVO_1692

and HVO_1693 would correspond to a complete heterotrimeric NAD-independent L-lactate dehydrogenase and would be promising candidates for having NAD-independent L-lactate dehydrogenase activity.

In *Pseudomonas*, *lldB* (PST_3338) and *lldC* (PST_3339) are clustered with genes for D-lactate dehydrogenase (PST_3340) and for a lactate permease (PST_3336). In the genomic vicinity of *Haloferax* ludBC is HVO_1696, a LctP family transport protein with 44% protein sequence identity to the *Pseudomonas* lactate permease. HVO_1697 shows 24% protein sequence identity to PST_3340. It cannot be excluded that those genes are also involved in lactate metabolism in *Haloferax*.

S9.g A gene cluster which may be responsible for conversion of urate to allantoin and may be part of a complete purine degradation pathway

We reconstructed a four-step pathway for conversion of urate to allantoate (Figure S2). According to KEGG (accessed April 2021), purines are degraded with intermediates being xanthine, urate and allantoate, the latter being further decomposed to CO_2 and glyoxylate. The four-step pathway is annotated according to a gene cluster from *B. subtilis* which codes for enzymes with the corresponding activities. The enzymes for the first and third pathway step are encoded by a single fused gene in *B. subtilis* (*pucL*) [209,210]. The enzymes are encoded by independent genes in haloarchaea (e.g. HVO_B0301, *pucL2*, corresponding to the N-terminus of *B. subtilis* PucL, HVO_B0300, *pucL1*, corresponding to the C-terminus of *B. subtilis* PucL). The second pathway step is catalyzed by the product of *pucM* [211]. The above assignments have also been made by KEGG (as of April 2021). The fourth step is catalyzed by allantoinase, a member of the cyclic amidohydrolase family. HVO_B0302 has only 30% protein sequence identity to characterized proteins with this activity (from *B. subtilis* and from *Salmonella* [212,213], but is slightly closer (33% protein sequence identity) to *Ralstonia* (*Burkholdia*) D-hydantoinase, which is involved in pyrimidine catabolism [214]. Purine and pyrimidine degradation pathways contain successive reactions which display similar chemistry and are catalyzed by homologous proteins [215]. KEGG (accessed April 2021) annotates HVO_B0302 as dihydropyrimidinase (D-hydantoinase), thus being involved in pyrimidine degradation, while we predict it as allantoinase, involved in purine degradation, taking the function of products from

clustered genes into account (Figure S2). There is a paralog with 61% protein sequence identity (HVO_A0303) which does not show function-related gene clustering. The pathway step subsequent to D-hydantoinase in pyrimidine catabolism is catalyzed by EC 3.5.1.6 (beta-ureidopropionase) and KEGG assigns this function to three *Haloferax* paralogs (as of April 2021), one being HVO_B0306 (encoded by *amaB4*). The chemically similar reaction in the purine degradation pathway is EC 3.5.1.116 (ureidoglycolate hydrolase) (Figure S2). Given the close proximity of the genes coding for other purine degradation pathway enzymes, we have assigned this function to HVO_B0306. Support comes from a characterized *Arabidopsis* homolog (34% protein sequence identity) [216,217] but a more closely related protein from *Geobacillus* (39% protein sequence identity) has a distinct function [218]. Two paralogs of HVO_B0306 in *Haloferax*, HVO_A0341 and HVO_2128, show more than 50% protein sequence identity but do not show function-related gene clustering and are currently not annotated as catalyzing EC 3.5.1.116. With these assignments, only two pathway gaps remain for xanthine degradation. The conversion of allantoate to ureido-glycolate, the substrate of EC 3.5.1.116, is yet unassigned. The other gap is the conversion of xanthine to urate. The genome region codes in a three-gene operon for the subunits of a molybdopterin-containing oxidoreductase (HVO_B0308, small subunit, HVO_B0309, large subunit, HVO_B0310, medium subunit) (Figure S2). These are distantly related to enzymes acting on various substrates (e.g. carbon monoxide). Among those is a probable xanthine dehydrogenase from *E. coli* which has been partially characterized [219]. It should be noted that xanthine oxidase activity has not been detected in *Hfx. volcanii* and that xanthine was not metabolized [220]. The proposed purine degradation pathway, if it exists, may not be expressed under optimal growth conditions. Finally, one gene in this cluster (HVO_B0303) codes for a transport protein for which xanthine and uracil are currently assigned as potential substrates. It shows 38% protein sequence identity to the product of *E. coli xanP*, a characterized xanthine permease [221].

Many of the assignments are insecure due to the low sequence similarity of functionally characterized homologs, and because homologs with distinct functions are equidistant for several of the enzymes. However, some enzymes are clearly related to purine catabolism and the clustering of these genes makes it likely that the complete region from HVO_B0299 to

HVO_B0310 codes for proteins involved in purine catabolism. These assignments will remain speculative until their experimental confirmation (or rejection).

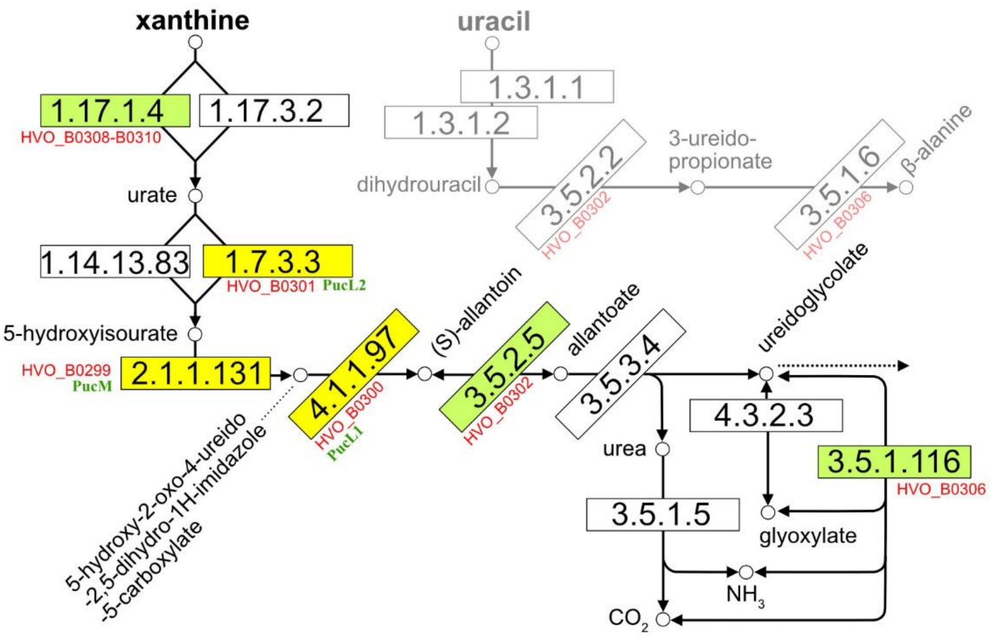

Figure S2. The proposed purine degradation pathway. Based on KEGG maps, the degradation pathways for purines (starting with xanthine, black, map 00230) and pyrimidines (starting with uracil, grey, map 00240) are shown. Enyzmes highlighted in yellow are assigned by KEGG and by us. Enzymes highlighted in green are tentatively assigned by us, but not by KEGG. For two proteins, KEGG makes an assignment to a chemically similar reaction of pyrimidine degradation.

S9.h *Hfx. volcanii* may have an enzyme with a nickel-pincer cofactor, generated by *larBCE*

HVO_2381 is annotated as LarC family protein, based on the product of the *Lactobacillus plantarum larC* gene which has been characterized [222]. The *Lactobacillus* LarC forms a complex with LarB and LarE, which generates the "nickel-pincer cofactor" of lactate racemase (LarA). The corresponding genes are part of an operon (*larA-larB-larC1-larC2-larD-larE*) and a regulatory gene (*larR*) is found nearby and transcribed in the opposite direction.

However, in the case of *Hfx. volcanii* DS2, the LarC family protein gene occurs without other *lar* homologs nearby, and not within an operon. In this and other species of haloarchaea, HVO_2381 is regularly found nearby an oppositely facing gene encoding AAA-type ATPase (CDC48 subfamily).

Later studies [223,224] identified many prokaryotic genomes with *larBCE* homologs that lack *larA*, and suggested from those findings that LarB, C and E can be used by enzymes other than LarA, and so for other functions. In *Hfx. volcanii* DS2 there are distant homologs of *larB* (HVO_0197) and of *larE* (HVO_0190) elsewhere in the chromosome but neither are part of an operon. In summary, the function of HVO_2381 is not clear even though a standard BLASTp search at GenBank (nr) returns a conserved protein domain match to PRK04194, "nickel pincer cofactor biosynthesis protein LarC" with high probability ($3.98x10^{-138}$) (accessed January, 2021).

S9.i Candidate genes which may catalyze cyclic di-AMP hydrolysis

Cyclic di-AMP (c-di-AMP) is generated from two molecules of ATP by diadenylate cyclase (encoded by *dacZ*) [225]. *Hfx. volcanii* DacZ (HVO_1660) has been characterized and it was shown that c-di-AMP levels must be tightly regulated [226]. c-di-AMP is degraded to pApA by phosphodiesterases [225]. As the level of this signalling molecule needs to be strictly controlled [227] [228], the degrading enzyme is also an important player. In *Hfx. volcanii*, this enzyme has yet not been identified but candidates have been proposed [225,229]. These are proteins with a DHH and DHHA1 domain that also have an N-terminal trkA_N domain. *Haloferax* candidates HVO_0990 and HVO_1690 were proposed recently, based on a phylogenetic profile [230]. Also proposed was HVO_0756, which, however, lacks the N-terminal trkA_N domain. Gene neighbourhood analysis did not provide additional clues to support or challenge the involvement of one or several of these candidates.

S9.j A homolog to RNaseZ

HVO_2763 shows 27% protein sequence identity to RNase Z (HVO_0144, *rnz*). The experimental characterization of HVO_2763 [231] specifically excluded its activity as a tRNA or

5S RNA processing enzyme and as an exonuclease generally. However, it did not reveal the physiological function of HVO_2763. The authors point out that this protein belongs to the metallo-beta-lactamase family, which is known to fulfil a diversity of functions. The authors deleted the gene, performed a transcriptome analysis, and found several genes to be downregulated in the deletion mutant. Among these were several transporter genes, and the authors note that the gene for HVO_2763 is adjacent to a pair of permease genes. However, SyntTax analysis shows that the permease genes are a strain-specific insertion in *Hfx. volcanii* DS2 when compared to closely related genomes. In other genomes, the adjacent gene corresponds to *lhr1* (HVO_2766) which codes for a helicase. The HVO_2763/2766 clustering is not only found in closely related strains, but in a considerable number of more distantly related haloarchaeal genomes. This should be considered when searching for a physiological function of HVO_2763. Additionally, three regulated genes are clustered and have since then been characterized (HVO_2055, *agl15*, HVO_2060, *agl8*, HVO_2061, *agl6*) [232]. They participate in a N-glycosylation pathway that was initially observed mainly under low-salt conditions. With respect to the downregulated transporter genes no annotation updates have been made since publication of the results.

S9.k A potential CO2 transporter (*dabAB*)

HVO_2410 and HVO_2411 probably function as carbon dioxide transporter, based on the identification of such transporters in *Halothiobacillus neapolitanus* [233]. The authors assume that CO_2 is directly transported and not a hydrated form (e.g. hydrogen carbonate), because transport activity is pH independent. This transporter is one of the CO_2 concentrating mechanisms. Orthologs are found in about two-thirds of the haloarchaeal genomes (108 of 166) according to OrthoDB. HVO_2411 belongs to the proton-conducting membrane transporter family (PFAM PF00361, InterPro IPR001750), which also contains subunits of the *nuo* complex and of the Mrp-type sodium/proton antiporter. In KEGG, HVO_2411 is annotated as NAD(P)H-quinone oxidoreductase subunit 5 (as of April 2021).

REFERENCES

1. Pfeiffer, F.; Schuster, S.C.; Broicher, A.; Falb, M.; Palm, P.; Rodewald, K.; Ruepp, A.; Soppa, J.; Tittor, J.; Oesterhelt, D. Evolution in the laboratory: the genome of *Halobacterium salinarum* strain R1 compared to that of strain NRC-1. *Genomics* **2008**, *91*, 335-346.
2. Ng, W.V.; Kennedy, S.P.; Mahairas, G.G.; Berquist, B.; Pan, M.; Shukla, H.D.; Lasky, S.R.; Baliga, N.S.; Thorsson, V.; Sbrogna, J., *et al.* Genome sequence of *Halobacterium* species NRC-1. *Proc. Natl. Acad. Sci. U. S. A.* **2000**, *97*, 12176-12181.
3. Ng, W.V.; Ciufo, S.A.; Smith, T.M.; Bumgarner, R.E.; Baskin, D.; Faust, J.; Hall, B.; Loretz, C.; Seto, J.; Slagel, J., *et al.* Snapshot of a large dynamic replicon in a halophilic archaeon: megaplasmid or minichromosome? *Genome Res.* **1998**, *8*, 1131-1141.
4. Pfeiffer, F.; Marchfelder, A.; Habermann, B.; Dyall-Smith, M.L. The genome sequence of the *Halobacterium salinarum* type strain is closely related to that of laboratory strains NRC-1 and R1. *Microbiol Resour Announc* **2019**, *8*, e00429-00419.
5. Pfeiffer, F.; Losensky, G.; Marchfelder, A.; Habermann, B.; Dyall-Smith, M. Whole-genome comparison between the type strain of *Halobacterium salinarum* (DSM 3754(T)) and the laboratory strains R1 and NRC-1. *Microbiologyopen* **2020**, *9*, e974.
6. Jaakkola, S.T.; Pfeiffer, F.; Ravantti, J.J.; Guo, Q.; Liu, Y.; Chen, X.; Ma, H.; Yang, C.; Oksanen, H.M.; Bamford, D.H. The complete genome of a viable archaeum isolated from 123-million-year-old rock salt. *Environ. Microbiol.* **2016**, *18*, 565-579.
7. Hartman, A.L.; Norais, C.; Badger, J.H.; Delmas, S.; Haldenby, S.; Madupu, R.; Robinson, J.; Khouri, H.; Ren, Q.; Lowe, T.M., *et al.* The complete genome sequence of *Haloferax volcanii* DS2, a model archaeon. *PLoS One* **2010**, *5*, e9605.
8. Tittes, C.; Schwarzer, S.; Pfeiffer, F.; Dyall-Smith, M.; Rodriguez-Franco, M.; Oksanen, H.M.; Quax, T.E.F. Cellular and genomic properties of *Haloferax gibbonsii* LR2-5, the host of euryarchaeal virus HFTV1. *Front. Microbiol.* **2021**, *12*, 625599.
9. Han, J.; Zhang, F.; Hou, J.; Liu, X.; Li, M.; Liu, H.; Cai, L.; Zhang, B.; Chen, Y.; Zhou, J., *et al.* Complete genome sequence of the metabolically versatile halophilic archaeon *Haloferax mediterranei*, a poly(3-hydroxybutyrate-co-3-hydroxyvalerate) producer. *J. Bacteriol.* **2012**, *194*, 4463-4464.
10. Falb, M.; Pfeiffer, F.; Palm, P.; Rodewald, K.; Hickmann, V.; Tittor, J.; Oesterhelt, D. Living with two extremes: conclusions from the genome sequence of *Natronomonas pharaonis. Genome Res.* **2005**, *15*, 1336-1343.
11. Dyall-Smith, M.L.; Pfeiffer, F.; Oberwinkler, T.; Klee, K.; Rampp, M.; Palm, P.; Gross, K.; Schuster, S.C.; Oesterhelt, D. Genome of the haloarchaeon *Natronomonas moolapensis*, a neutrophilic member of a previously haloalkaliphilic genus. *Genome Announc* **2013**, *1*, e0009513.
12. Bolhuis, H.; Palm, P.; Wende, A.; Falb, M.; Rampp, M.; Rodriguez-Valera, F.; Pfeiffer, F.; Oesterhelt, D. The genome of the square archaeon *Haloquadratum walsbyi* : life at the limits of water activity. *BMC Genomics* **2006**, *7*, 169.
13. Dyall-Smith, M.L.; Pfeiffer, F.; Klee, K.; Palm, P.; Gross, K.; Schuster, S.C.; Rampp, M.; Oesterhelt, D. *Haloquadratum walsbyi*: limited diversity in a global pond. *PLoS One* **2011**, *6*, e20968.
14. Siddaramappa, S.; Challacombe, J.F.; Decastro, R.E.; Pfeiffer, F.; Sastre, D.E.; Gimenez, M.I.; Paggi, R.A.; Detter, J.C.; Davenport, K.W.; Goodwin, L.A., *et al.* A comparative

genomics perspective on the genetic content of the alkaliphilic haloarchaeon *Natrialba magadii* ATCC 43099^T. *BMC Genomics* **2012**, *13*, 165.
15. Baliga, N.S.; Bonneau, R.; Facciotti, M.T.; Pan, M.; Glusman, G.; Deutsch, E.W.; Shannon, P.; Chiu, Y.; Weng, R.S.; Gan, R.R., *et al.* Genome sequence of *Haloarcula marismortui*: a halophilic archaeon from the Dead Sea. *Genome Res.* **2004**, *14*, 2221-2234.
16. Demaere, M.Z.; Williams, T.J.; Allen, M.A.; Brown, M.V.; Gibson, J.A.; Rich, J.; Lauro, F.M.; Dyall-Smith, M.; Davenport, K.W.; Woyke, T., *et al.* High level of intergenera gene exchange shapes the evolution of haloarchaea in an isolated Antarctic lake. *Proc. Natl. Acad. Sci. U. S. A.* **2013**, *110*, 16939-16944.
17. Kerscher, L.; Oesterhelt, D. Ferredoxin is the coenzyme of α-ketoacid oxidoreductases in *Halobacterium halobium*. *FEBS Lett.* **1977**, *83*, 197-201.
18. Plaga, W.; Lottspeich, F.; Oesterhelt, D. Improved purification, crystallization and primary structure of pyruvate:ferredoxin oxidoreductase from *Halobacterium halobium*. *Eur. J. Biochem.* **1992**, *205*, 391-397.
19. Kerscher, L.; Oesterhelt, D. Purification and properties of two 2-oxoacid:ferredoxin oxidoreductases from *Halobacterium halobium*. *Eur. J. Biochem.* **1981**, *116*, 587-594.
20. Kerscher, L.; Oesterhelt, D. The catalytic mechanism of 2-oxoacid:ferredoxin oxidoreductases from *Halobacterium halobium*. One-electron transfer at two distinct steps of the catalytic cycle. *Eur. J. Biochem.* **1981**, *116*, 595-600.
21. Kuprat, T.; Ortjohann, M.; Johnsen, U.; Schonheit, P. Glucose metabolism and acetate switch in Archaea: the enzymes in *Haloferax volcanii*. *J. Bacteriol.* **2021**, *203*, e00690-00620.
22. Zafrilla, B.; Martinez-Espinosa, R.M.; Bonete, M.J.; Butt, J.N.; Richardson, D.J.; Gates, A.J. A haloarchaeal ferredoxin electron donor that plays an essential role in nitrate assimilation. *Biochem. Soc. Trans.* **2011**, *39*, 1844-1848.
23. Rich, P.R.; Marechal, A. The mitochondrial respiratory chain. *Essays Biochem.* **2010**, *47*, 1-23.
24. Sousa, P.M.F.; Videira, M.A.M.; Bohn, A.; Hood, B.L.; Conrads, T.P.; Goulao, L.F.; Melo, A.M.P. The aerobic respiratory chain of *Escherichia coli*: from genes to supercomplexes. *Microbiology (Reading)* **2012**, *158*, 2408-2418.
25. Kaila, V.R.I.; Wikstrom, M. Architecture of bacterial respiratory chains. *Nat. Rev. Microbiol.* **2021**, *19*, 319-330.
26. Leif, H.; Sled, V.D.; Ohnishi, T.; Weiss, H.; Friedrich, T. Isolation and characterization of the proton-translocating NADH: ubiquinone oxidoreductase from *Escherichia coli*. *Eur. J. Biochem.* **1995**, *230*, 538-548.
27. Braun, M.; Bungert, S.; Friedrich, T. Characterization of the overproduced NADH dehydrogenase fragment of the NADH:ubiquinone oxidoreductase (complex I) from *Escherichia coli*. *Biochemistry* **1998**, *37*, 1861-1867.
28. Sreeramulu, K.; Schmidt, C.L.; Schafer, G.; Anemuller, S. Studies of the electron transport chain of the euryarcheon *Halobacterium salinarum*: indications for a type II NADH dehydrogenase and a complex III analog. *J Bioenerg Biomembr* **1998**, *30*, 443-453.
29. Zhang, P.; Battchikova, N.; Paakkarinen, V.; Katoh, H.; Iwai, M.; Ikeuchi, M.; Pakrasi, H.B.; Ogawa, T.; Aro, E.M. Isolation, subunit composition and interaction of the NDH-1 complexes from *Thermosynechococcus elongatus* BP-1. *Biochem. J.* **2005**, *390*, 513-520.

30. Schuller, J.M.; Birrell, J.A.; Tanaka, H.; Konuma, T.; Wulfhorst, H.; Cox, N.; Schuller, S.K.; Thiemann, J.; Lubitz, W.; Setif, P.*, et al.* Structural adaptations of photosynthetic complex I enable ferredoxin-dependent electron transfer. *Science* **2019**, *363*, 257-260.
31. Pan, X.; Cao, D.; Xie, F.; Xu, F.; Su, X.; Mi, H.; Zhang, X.; Li, M. Structural basis for electron transport mechanism of complex I-like photosynthetic NAD(P)H dehydrogenase. *Nat Commun* **2020**, *11*, 610.
32. Gomes, C.M.; Bandeiras, T.M.; Teixeira, M. A new type-II NADH dehydrogenase from the archaeon *Acidianus ambivalens*: characterization and in vitro reconstitution of the respiratory chain. *J Bioenerg Biomembr* **2001**, *33*, 1-8.
33. Bandeiras, T.M.; Salgueiro, C.; Kletzin, A.; Gomes, C.M.; Teixeira, M. *Acidianus ambivalens* type-II NADH dehydrogenase: genetic characterisation and identification of the flavin moiety as FMN. *FEBS Lett.* **2002**, *531*, 273-277.
34. Brito, J.A.; Sousa, F.L.; Stelter, M.; Bandeiras, T.M.; Vonrhein, C.; Teixeira, M.; Pereira, M.M.; Archer, M. Structural and functional insights into sulfide:quinone oxidoreductase. *Biochemistry* **2009**, *48*, 5613-5622.
35. Jaworowski, A.; Campbell, H.D.; Poulis, M.I.; Young, I.G. Genetic identification and purification of the respiratory NADH dehydrogenase of *Escherichia coli*. *Biochemistry* **1981**, *20*, 2041-2047.
36. Liu, J.; Krulwich, T.A.; Hicks, D.B. Purification of two putative type II NADH dehydrogenases with different substrate specificities from alkaliphilic *Bacillus pseudofirmus* OF4. *Biochim Biophys Acta* **2008**, *1777*, 453-461.
37. Marreiros, B.C.; Sena, F.V.; Sousa, F.M.; Batista, A.P.; Pereira, M.M. Type II NADH:quinone oxidoreductase family: phylogenetic distribution, structural diversity and evolutionary divergences. *Environ. Microbiol.* **2016**, *18*, 4697-4709.
38. Lee, T.-X.; Metzger, S.U.; Cho, Y.S.; Whitmarsh, J.; Kallas, T. Modification of inhibitor binding sites in the cytochrome bf complex by directed mutagenesis of cytochrome b6 in *Synechococcus* sp. PCC 7002. *Biochim. Biophys. Acta* **2001**, *1504*, 235-247.
39. Scharf, B.; Wittenberg, R.; Engelhard, M. Electron transfer proteins from the haloalkaliphilic archaeon *Natronobacterium pharaonis*: possible components of the respiratory chain include cytochrome bc and a terminal oxidase cytochrome ba3. *Biochemistry* **1997**, *36*, 4471-4479.
40. Mattar, S. Molekularbiologische und Biochemische Charakterisierung zweier Komplexe der Atmungskette von *Natronobacterium pharaonis*. Ruhr-Universität Bochum, Bochum.
41. Kletzin, A.; Heimerl, T.; Flechsler, J.; van Niftrik, L.; Rachel, R.; Klingl, A. Cytochromes c in Archaea: distribution, maturation, cell architecture, and the special case of *Ignicoccus hospitalis*. *Front. Microbiol.* **2015**, *6*, 439.
42. Sreeramulu, K. Purification and partial characterization of cytochrome c552 from *Halobacterium salinarium*. *Indian Journal of Biochemistry and Biophysics* **2003**, *40*, 274-277.
43. Mattar, S.; Scharf, B.; Kent, S.B.; Rodewald, K.; Oesterhelt, D.; Engelhard, M. The primary structure of halocyanin, an archaeal blue copper protein, predicts a lipid anchor for membrane fixation. *J. Biol. Chem.* **1994**, *269*, 14939-14945.
44. Scharf, B.; Engelhard, M. Halocyanin, an archaebacterial blue copper protein (type I) from *Natronobacterium pharaonis*. *Biochemistry* **1993**, *32*, 12894-12900.

45. Hildebrandt, P.; Matysik, J.; Schrader, B.; Scharf, B.; Engelhard, M. Raman spectroscopic study of the blue copper protein halocyanin from *Natronobacterium pharaonis*. *Biochemistry* **1994**, *33*, 11426-11431.
46. Storf, S.; Pfeiffer, F.; Dilks, K.; Chen, Z.Q.; Imam, S.; Pohlschroder, M. Mutational and bioinformatic analysis of haloarchaeal lipobox-containing proteins. *Archaea* **2010**, *2010*, Article ID 410975.
47. Mattar, S.; Engelhard, M. Cytochrome ba3 from *Natronobacterium pharaonis*--an archaeal four-subunit cytochrome-c-type oxidase. *Eur. J. Biochem.* **1997**, *250*, 332-341.
48. Guo, R.; Gu, J.; Zong, S.; Wu, M.; Yang, M. Structure and mechanism of mitochondrial electron transport chain. *Biomed J* **2018**, *41*, 9-20.
49. Schaefer, G.; Purschke, W.G.; Gleissner, M.; Schmidt, C.L. Respiratory chains of archaea and extremophiles. *Biochim. Biophys. Acta* **1996**, *1275*, 16-20.
50. Refojo, P.N.; Sena, F.V.; Calisto, F.; Sousa, F.M.; Pereira, M.M. The plethora of membrane respiratory chains in the phyla of life. *Adv. Microb. Physiol.* **2019**, *74*, 331-414.
51. Tanaka, M.; Ogawa, N.; Ihara, K.; Sugiyama, Y.; Mukohata, Y. Cytochrome aa(3) in *Haloferax volcanii*. *J. Bacteriol.* **2002**, *184*, 840-845.
52. Fujiwara, T.; Fukumori, Y.; Yamanaka, T. Purification and properties of *Halobacterium halobium* "cytochrome aa3" which lacks CuA and CuB. *J Biochem (Tokyo)* **1989**, *105*, 287-292.
53. Denda, K.; Fujiwara, T.; Seki, M.; Yoshida, M.; Fukumori, Y.; Yamanaka, T. Molecular cloning of the cytochrome aa3 gene from the archaeon (Archaebacterium) *Halobacterium halobium*. *Biochem Biophys Res Commun* **1991**, *181*, 316-322.
54. Ishikawa, R.; Ishido, Y.; Tachikawa, A.; Kawasaki, H.; Matsuzawa, H.; Wakagi, T. *Aeropyrum pernix* K1, a strictly aerobic and hyperthermophilic archaeon, has two terminal oxidases, cytochrome ba3 and cytochrome aa3. *Arch. Microbiol.* **2002**, *179*, 42-49.
55. Pfeiffer, F.; Oesterhelt, D. A manual curation strategy to improve genome annotation: application to a set of haloarchael genomes. *Life (Basel)* **2015**, *5*, 1427-1444.
56. Jolley, K.A.; Maddocks, D.G.; Gyles, S.L.; Mullan, Z.; Tang, S.L.; Dyall-Smith, M.L.; Hough, D.W.; Danson, M.J. 2-Oxoacid dehydrogenase multienzyme complexes in the halophilic Archaea? Gene sequences and protein structural predictions. *Microbiology (Reading)* **2000**, *146 (Pt 5)*, 1061-1069.
57. van Ooyen, J.; Soppa, J. Three 2-oxoacid dehydrogenase operons in *Haloferax volcanii*: expression, deletion mutants and evolution. *Microbiology* **2007**, *153*, 3303-3313.
58. Al-Mailem, D.M.; Hough, D.W.; Danson, M.J. The 2-oxoacid dehydrogenase multienzyme complex of *Haloferax volcanii*. *Extremophiles* **2008**, *12*, 89-96.
59. Wanner, C.; Soppa, J. Functional role for a 2-oxo acid dehydrogenase in the halophilic archaeon *Haloferax volcanii*. *J. Bacteriol.* **2002**, *184*, 3114-3121.
60. Sisignano, M.; Morbitzer, D.; Gatgens, J.; Oldiges, M.; Soppa, J. A 2-oxoacid dehydrogenase complex of *Haloferax volcanii* is essential for growth on isoleucine but not on other branched-chain amino acids. *Microbiology* **2010**, *156*, 521-529.
61. Heath, C.; Posner, M.G.; Aass, H.C.; Upadhyay, A.; Scott, D.J.; Hough, D.W.; Danson, M.J. The 2-oxoacid dehydrogenase multi-enzyme complex of the archaeon *Thermoplasma acidophilum* - recombinant expression, assembly and characterization. *FEBS J.* **2007**, *274*, 5406-5415.

62. Huang, M.; Oppermann-Sanio, F.B.; Steinbuchel, A. Biochemical and molecular characterization of the *Bacillus subtilis* acetoin catabolic pathway. *J. Bacteriol.* **1999**, *181*, 3837-3841.
63. Oppermann, F.B.; Schmidt, B.; Steinbuchel, A. Purification and characterization of acetoin:2,6-dichlorophenolindophenol oxidoreductase, dihydrolipoamide dehydrogenase, and dihydrolipoamide acetyltransferase of the *Pelobacter carbinolicus* acetoin dehydrogenase enzyme system. *J. Bacteriol.* **1991**, *173*, 757-767.
64. Yoshida, A.; Tomita, T.; Atomi, H.; Kuzuyama, T.; Nishiyama, M. Lysine biosynthesis of *Thermococcus kodakarensis* with the capacity to function as an ornithine biosynthetic system. *J. Biol. Chem.* **2016**, *291*, 21630-21643.
65. Ouchi, T.; Tomita, T.; Horie, A.; Yoshida, A.; Takahashi, K.; Nishida, H.; Lassak, K.; Taka, H.; Mineki, R.; Fujimura, T., *et al.* Lysine and arginine biosyntheses mediated by a common carrier protein in *Sulfolobus*. *Nat. Chem. Biol.* **2013**, *9*, 277-283.
66. Horie, A.; Tomita, T.; Saiki, A.; Kono, H.; Taka, H.; Mineki, R.; Fujimura, T.; Nishiyama, C.; Kuzuyama, T.; Nishiyama, M. Discovery of proteinaceous N-modification in lysine biosynthesis of *Thermus thermophilus*. *Nat. Chem. Biol.* **2009**, *5*, 673-679.
67. Hochuli, M.; Patzelt, H.; Oesterhelt, D.; Wuthrich, K.; Szyperski, T. Amino acid biosynthesis in the halophilic archaeon *Haloarcula hispanica*. *J. Bacteriol.* **1999**, *181*, 3226-3237.
68. Jevtic, Z.; Stoll, B.; Pfeiffer, F.; Sharma, K.; Urlaub, H.; Marchfelder, A.; Lenz, C. The response of *Haloferax volcanii* to salt and temperature stress: a proteome study by label-free mass spectrometry. *Proteomics* **2019**, *19*, e1800491.
69. White, R.H. L-Aspartate semialdehyde and a 6-deoxy-5-ketohexose 1-phosphate are the precursors to the aromatic amino acids in *Methanocaldococcus jannaschii*. *Biochemistry* **2004**, *43*, 7618-7627.
70. Gulko, M.K.; Dyall-Smith, M.; Gonzalez, O.; Oesterhelt, D. How do haloarchaea synthesize aromatic amino acids? *PLoS One* **2014**, *9*, e107475.
71. Morar, M.; White, R.H.; Ealick, S.E. Structure of 2-amino-3,7-dideoxy-D-threo-hept-6-ulosonic acid synthase, a catalyst in the archaeal pathway for the biosynthesis of aromatic amino acids. *Biochemistry* **2007**, *46*, 10562-10571.
72. Large, A.; Stamme, C.; Lange, C.; Duan, Z.; Allers, T.; Soppa, J.; Lund, P.A. Characterization of a tightly controlled promoter of the halophilic archaeon *Haloferax volcanii* and its use in the analysis of the essential *cct1* gene. *Mol. Microbiol.* **2007**, *66*, 1092-1106.
73. Kaminskas, E.; Kimhi, Y.; Magasanik, B. Urocanase and N-formimino-L-glutamate formiminohydrolase of *Bacillus subtilis*, two enzymes of the histidine degradation pathway. *J. Biol. Chem.* **1970**, *245*, 3536-3544.
74. Yu, Y.; Liang, Y.H.; Brostromer, E.; Quan, J.M.; Panjikar, S.; Dong, Y.H.; Su, X.D. A catalytic mechanism revealed by the crystal structures of the imidazolonepropionase from *Bacillus subtilis*. *J. Biol. Chem.* **2006**, *281*, 36929-36936.
75. Oda, M.; Sugishita, A.; Furukawa, K. Cloning and nucleotide sequences of histidase and regulatory genes in the *Bacillus subtilis hut* operon and positive regulation of the operon. *J. Bacteriol.* **1988**, *170*, 3199-3205.
76. Hartwell, L.H.; Magasanik, B. The molecular basis of histidase induction in *Bacillus subtilis*. *J. Mol. Biol.* **1963**, *7*, 401-420.

77. Kiljunen, S.; Pajunen, M.I.; Dilks, K.; Storf, S.; Pohlschroder, M.; Savilahti, H. Generation of comprehensive transposon insertion mutant library for the model archaeon, *Haloferax volcanii*, and its use for gene discovery. *BMC Biol.* **2014**, *12*, 103.
78. Collins, M.; Afolayan, S.; Igiraneza, A.B.; Schiller, H.; Krespan, E.; Beiting, D.P.; Dyall-Smith, M.; Pfeiffer, F.; Pohlschroder, M. Mutations affecting HVO_1357 or HVO_2248 cause hypermotility in *Haloferax volcanii*, suggesting roles in motility regulation. *Genes (Basel)* **2020**, *12*, 58.
79. Schulze, S.; Adams, Z.; Cerletti, M.; De Castro, R.; Ferreira-Cerca, S.; Fufezan, C.; Gimenez, M.I.; Hippler, M.; Jevtic, Z.; Knuppel, R., *et al.* The Archaeal Proteome Project advances knowledge about archaeal cell biology through comprehensive proteomics. *Nat Commun* **2020**, *11*, 3145.
80. Laass, S.; Monzon, V.A.; Kliemt, J.; Hammelmann, M.; Pfeiffer, F.; Forstner, K.U.; Soppa, J. Characterization of the transcriptome of *Haloferax volcanii*, grown under four different conditions, with mixed RNA-Seq. *PLoS One* **2019**, *14*, e0215986.
81. Bali, S.; Lawrence, A.D.; Lobo, S.A.; Saraiva, L.M.; Golding, B.T.; Palmer, D.J.; Howard, M.J.; Ferguson, S.J.; Warren, M.J. Molecular hijacking of siroheme for the synthesis of heme and d1 heme. *Proc. Natl. Acad. Sci. U. S. A.* **2011**, *108*, 18260-18265.
82. Kuhner, M.; Haufschildt, K.; Neumann, A.; Storbeck, S.; Streif, J.; Layer, G. The alternative route to heme in the methanogenic archaeon *Methanosarcina barkeri*. *Archaea* **2014**, *2014*, Article ID 327637.
83. Dailey, H.A.; Dailey, T.A.; Gerdes, S.; Jahn, D.; Jahn, M.; O'Brian, M.R.; Warren, M.J. Prokaryotic heme biosynthesis: multiple pathways to a common essential product. *Microbiol Mol Biol Rev* **2017**, *81*.
84. Blanche, F.; Thibaut, D.; Debussche, L.; Hertle, R.; Zipfel, F.; Müller, G. Parallels and decisive differences in vitamin B12 biosyntheses. *Angewandte Chemie International Edition in English* **1993**, *32*, 1651-1653.
85. Moore, S.J.; Lawrence, A.D.; Biedendieck, R.; Deery, E.; Frank, S.; Howard, M.J.; Rigby, S.E.; Warren, M.J. Elucidation of the anaerobic pathway for the corrin component of cobalamin (vitamin B12). *Proc. Natl. Acad. Sci. U. S. A.* **2013**, *110*, 14906-14911.
86. Raux, E.; Leech, H.K.; Beck, R.; Schubert, H.L.; Santander, P.J.; Roessner, C.A.; Scott, A.I.; Martens, J.H.; Jahn, D.; Thermes, C., *et al.* Identification and functional analysis of enzymes required for precorrin-2 dehydrogenation and metal ion insertion in the biosynthesis of sirohaem and cobalamin in *Bacillus megaterium*. *Biochem. J.* **2003**, *370*, 505-516.
87. Brindley, A.A.; Raux, E.; Leech, H.K.; Schubert, H.L.; Warren, M.J. A story of chelatase evolution: identification and characterization of a small 13-15-kDa "ancestral" cobaltochelatase (CbiXS) in the archaea. *J. Biol. Chem.* **2003**, *278*, 22388-22395.
88. Yin, J.; Xu, L.X.; Cherney, M.M.; Raux-Deery, E.; Bindley, A.A.; Savchenko, A.; Walker, J.R.; Cuff, M.E.; Warren, M.J.; James, M.N. Crystal structure of the vitamin B12 biosynthetic cobaltochelatase, CbiXS, from *Archaeoglobus fulgidus*. *J Struct Funct Genomics* **2006**, *7*, 37-50.
89. Raux, E.; Thermes, C.; Heathcote, P.; Rambach, A.; Warren, M.J. A role for *Salmonella typhimurium* cbiK in cobalamin (vitamin B12) and siroheme biosynthesis. *J. Bacteriol.* **1997**, *179*, 3202-3212.

90. Lobo, S.A.; Brindley, A.A.; Romao, C.V.; Leech, H.K.; Warren, M.J.; Saraiva, L.M. Two distinct roles for two functional cobaltochelatases (CbiK) in *Desulfovibrio vulgaris* hildenborough. *Biochemistry* **2008**, *47*, 5851-5857.
91. Spencer, J.B.; Stolowich, N.J.; Roessner, C.A.; Scott, A.I. The *Escherichia coli cysG* gene encodes the multifunctional protein, siroheme synthase. *FEBS Lett.* **1993**, *335*, 57-60.
92. Stroupe, M.E.; Leech, H.K.; Daniels, D.S.; Warren, M.J.; Getzoff, E.D. CysG structure reveals tetrapyrrole-binding features and novel regulation of siroheme biosynthesis. *Nat. Struct. Biol.* **2003**, *10*, 1064-1073.
93. Pennington, J.M.; Kemp, M.; McGarry, L.; Chen, Y.; Stroupe, M.E. Siroheme synthase orients substrates for dehydrogenase and chelatase activities in a common active site. *Nat Commun* **2020**, *11*, 864.
94. Storbeck, S.; Rolfes, S.; Raux-Deery, E.; Warren, M.J.; Jahn, D.; Layer, G. A novel pathway for the biosynthesis of heme in Archaea: genome-based bioinformatic predictions and experimental evidence. *Archaea* **2010**, *2010*, 175050.
95. Trzebiatowski, J.R.; O'Toole, G.A.; Escalante-Semerena, J.C. The cobT gene of *Salmonella typhimurium* encodes the NaMN: 5,6-dimethylbenzimidazole phosphoribosyltransferase responsible for the synthesis of N1-(5-phospho-alpha-D-ribosyl)-5,6-dimethylbenzimidazole, an intermediate in the synthesis of the nucleotide loop of cobalamin. *J. Bacteriol.* **1994**, *176*, 3568-3575.
96. Rodionov, D.A.; Vitreschak, A.G.; Mironov, A.A.; Gelfand, M.S. Comparative genomics of the vitamin B12 metabolism and regulation in prokaryotes. *J. Biol. Chem.* **2003**, *278*, 41148-41159.
97. O'Toole, G.A.; Trzebiatowski, J.R.; Escalante-Semerena, J.C. The cobC gene of *Salmonella typhimurium* codes for a novel phosphatase involved in the assembly of the nucleotide loop of cobalamin. *J. Biol. Chem.* **1994**, *269*, 26503-26511.
98. Brushaber, K.R.; O'Toole, G.A.; Escalante-Semerena, J.C. CobD, a novel enzyme with L-threonine-O-3-phosphate decarboxylase activity, is responsible for the synthesis of (R)-1-amino-2-propanol O-2-phosphate, a proposed new intermediate in cobalamin biosynthesis in *Salmonella typhimurium* LT2. *J. Biol. Chem.* **1998**, *273*, 2684-2691.
99. Debussche, L.; Couder, M.; Thibaut, D.; Cameron, B.; Crouzet, J.; Blanche, F. Assay, purification, and characterization of cobaltochelatase, a unique complex enzyme catalyzing cobalt insertion in hydrogenobyrinic acid a,c-diamide during coenzyme B12 biosynthesis in *Pseudomonas denitrificans*. *J. Bacteriol.* **1992**, *174*, 7445-7451.
100. Jensen, P.E.; Gibson, L.C.; Henningsen, K.W.; Hunter, C.N. Expression of the *chlI*, *chlD*, and *chlH* genes from the Cyanobacterium *Synechocystis* PCC6803 in *Escherichia coli* and demonstration that the three cognate proteins are required for magnesium-protoporphyrin chelatase activity. *J. Biol. Chem.* **1996**, *271*, 16662-16667.
101. Jensen, P.E.; Gibson, L.C.; Hunter, C.N. Determinants of catalytic activity with the use of purified I, D and H subunits of the magnesium protoporphyrin IX chelatase from *Synechocystis* PCC6803. *Biochem. J.* **1998**, *334 (Pt 2)*, 335-344.
102. Palmer, D.J.; Schroeder, S.; Lawrence, A.D.; Deery, E.; Lobo, S.A.; Saraiva, L.M.; McLean, K.J.; Munro, A.W.; Ferguson, S.J.; Pickersgill, R.W., *et al.* The structure, function and properties of sirohaem decarboxylase--an enzyme with structural homology to a transcription factor family that is part of the alternative haem biosynthesis pathway. *Mol. Microbiol.* **2014**, *93*, 247-261.

103. Haufschildt, K.; Schmelz, S.; Kriegler, T.M.; Neumann, A.; Streif, J.; Arai, H.; Heinz, D.W.; Layer, G. The crystal structure of siroheme decarboxylase in complex with iron-uroporphyrin III reveals two essential histidine residues. *J. Mol. Biol.* **2014**, *426*, 3272-3286.
104. Bab-Dinitz, E.; Shmuely, H.; Maupin-Furlow, J.; Eichler, J.; Shaanan, B. *Haloferax volcanii* PitA: an example of functional interaction between the Pfam chlorite dismutase and antibiotic biosynthesis monooxygenase families? *Bioinformatics* **2006**, *22*, 671-675.
105. Kosugi, N.; Araki, T.; Fujita, J.; Tanaka, S.; Fujiwara, T. Growth phenotype analysis of heme synthetic enzymes in a halophilic archaeon, *Haloferax volcanii*. *PLoS One* **2017**, *12*, e0189913.
106. Cheeseman, P.; Toms-Wood, A.; Wolfe, R.S. Isolation and properties of a fluorescent compound, factor 420 , from *Methanobacterium* strain M.o.H. *J. Bacteriol.* **1972**, *112*, 527-531.
107. Eirich, L.D.; Vogels, G.D.; Wolfe, R.S. Distribution of coenzyme F420 and properties of its hydrolytic fragments. *J. Bacteriol.* **1979**, *140*, 20-27.
108. Jaenchen, R.; Schonheit, P.; Thauer, R.K. Studies on the biosynthesis of coenzyme F420 in methanogenic bacteria. *Arch. Microbiol.* **1984**, *137*, 362-365.
109. Lin, X.L.; White, R.H. Occurrence of coenzyme F420 and its gamma-monoglutamyl derivative in nonmethanogenic archaebacteria. *J. Bacteriol.* **1986**, *168*, 444-448.
110. Greening, C.; Ahmed, F.H.; Mohamed, A.E.; Lee, B.M.; Pandey, G.; Warden, A.C.; Scott, C.; Oakeshott, J.G.; Taylor, M.C.; Jackson, C.J. Physiology, Biochemistry, and Applications of F420- and Fo-Dependent Redox Reactions. *Microbiol Mol Biol Rev* **2016**, *80*, 451-493.
111. Grochowski, L.L.; Xu, H.; White, R.H. Identification and characterization of the 2-phospho-L-lactate guanylyltransferase involved in coenzyme F420 biosynthesis. *Biochemistry* **2008**, *47*, 3033-3037.
112. Graupner, M.; Xu, H.; White, R.H. Characterization of the 2-phospho-L-lactate transferase enzyme involved in coenzyme F(420) biosynthesis in *Methanococcus jannaschii*. *Biochemistry* **2002**, *41*, 3754-3761.
113. Bashiri, G.; Antoney, J.; Jirgis, E.N.M.; Shah, M.V.; Ney, B.; Copp, J.; Stuteley, S.M.; Sreebhavan, S.; Palmer, B.; Middleditch, M., *et al.* A revised biosynthetic pathway for the cofactor F420 in prokaryotes. *Nat Commun* **2019**, *10*, 1558.
114. Braga, D.; Last, D.; Hasan, M.; Guo, H.; Leichnitz, D.; Uzum, Z.; Richter, I.; Schalk, F.; Beemelmanns, C.; Hertweck, C., *et al.* Metabolic pathway rerouting in *Paraburkholderia rhizoxinica* evolved long-overlooked derivatives of coenzyme F420. *ACS Chem. Biol.* **2019**, *14*, 2088-2094.
115. de Wit, L.E.A.; Eker, A.P.M. 8-Hydroxy-5-deazaflavin-dependent electron transfer in the extreme halophile *Halobacterium cutirubrum*. *FEMS Microbiol. Lett.* **1987**, *48*, 121-125.
116. Selengut, J.D.; Haft, D.H. Unexpected abundance of coenzyme F(420)-dependent enzymes in *Mycobacterium tuberculosis* and other actinobacteria. *J. Bacteriol.* **2010**, *192*, 5788-5798.
117. Kunow, J.; Schwoërer, B.; Stetter, K.O.; Thauer, R.K. A F420-dependent NADP reductase in the extremely thermophilic sulfate-reducing *Archaeoglobus fulgidus*. *Arch. Microbiol.* **1993**, *160*, 199-205.
118. Reinhardt, A.; Johnsen, U.; Schonheit, P. L-Rhamnose catabolism in archaea. *Mol. Microbiol.* **2019**, *111*, 1093-1108.

119. Klein, A.R.; Berk, H.; Purwantini, E.; Daniels, L.; Thauer, R.K. Si-face stereospecificity at C5 of coenzyme F420 for F420-dependent glucose-6-phosphate dehydrogenase from *Mycobacterium smegmatis* and F420-dependent alcohol dehydrogenase from *Methanoculleus thermophilicus*. *Eur. J. Biochem.* **1996**, *239*, 93-97.
120. Aufhammer, S.W.; Warkentin, E.; Berk, H.; Shima, S.; Thauer, R.K.; Ermler, U. Coenzyme binding in F420-dependent secondary alcohol dehydrogenase, a member of the bacterial luciferase family. *Structure* **2004**, *12*, 361-370.
121. Johnson, E.F.; Mukhopadhyay, B. A new type of sulfite reductase, a novel coenzyme F420-dependent enzyme, from the methanarchaeon *Methanocaldococcus jannaschii*. *J. Biol. Chem.* **2005**, *280*, 38776-38786.
122. Jones, D.L.; Baxter, B.K. DNA repair and photoprotection: mechanisms of overcoming environmental ultraviolet radiation exposure in halophilic Archaea. *Front. Microbiol.* **2017**, *8*, 1882.
123. Perez-Arnaiz, P.; Dattani, A.; Smith, V.; Allers, T. *Haloferax volcanii*-a model archaeon for studying DNA replication and repair. *Open Biol* **2020**, *10*, 200293.
124. McCready, S. The repair of ultraviolet light-induced DNA damage in the halophilic archaebacteria, *Halobacterium cutirubrum*, *Halobacterium halobium* and *Haloferax volcanii*. *Mutat. Res.* **1996**, *364*, 25-32.
125. Eker, A.P.M.; Formenoy, L.; Wit, L.E.A.D. Photoreactivation in the extreme halophilic archaebacterium *Halobacterium cutirubrum*. *Photochemistry and Photobiology* **1991**, *53*, 643-651.
126. Iwasa, T.; Tokutomi, S.; Tokunaga, F. Photoreactivation of *Halobacterium halobium*: action spectrum and role of pigmentation. *Photochemistry and Photobiology* **1988**, *47*, 267-270.
127. Takao, M.; Kobayashi, T.; Oikawa, A.; Yasui, A. Tandem arrangement of photolyase and superoxide dismutase genes in *Halobacterium halobium*. *J. Bacteriol.* **1989**, *171*, 6323-6329.
128. McCready, S.; Marcello, L. Repair of UV damage in *Halobacterium salinarum*. *Biochem. Soc. Trans.* **2003**, *31*, 694-698.
129. Baliga, N.S.; Bjork, S.J.; Bonneau, R.; Pan, M.; Iloanusi, C.; Kottemann, M.C.; Hood, L.; DiRuggiero, J. Systems level insights into the stress response to UV radiation in the halophilic archaeon *Halobacterium* NRC-1. *Genome Res.* **2004**, *14*, 1025-1035.
130. Brudler, R.; Hitomi, K.; Daiyasu, H.; Toh, H.; Kucho, K.-i.; Ishiura, M.; Kanehisa, M.; Roberts, V.A.; Todo, T.; Tainer, J.A., *et al.* Identification of a new cryptochrome class. *Mol. Cell* **2003**, *11*, 59-67.
131. Kleine, T.; Lockhart, P.; Batschauer, A. An *Arabidopsis* protein closely related to *Synechocystis* cryptochrome is targeted to organelles. *Plant J.* **2003**, *35*, 93-103.
132. Selby, C.P.; Sancar, A. A cryptochrome/photolyase class of enzymes with single-stranded DNA-specific photolyase activity. *Proc. Natl. Acad. Sci. U. S. A.* **2006**, *103*, 17696-17700.
133. Zhang, F.; Scheerer, P.; Oberpichler, I.; Lamparter, T.; Krauss, N. Crystal structure of a prokaryotic (6-4) photolyase with an Fe-S cluster and a 6,7-dimethyl-8-ribityllumazine antenna chromophore. *Proc. Natl. Acad. Sci. U. S. A.* **2013**, *110*, 7217-7222.
134. White, R.H. Analysis and characterization of the folates in the nonmethanogenic archaebacteria. *J. Bacteriol.* **1988**, *170*, 4608-4612.

135. de Crecy-Lagard, V. Variations in metabolic pathways create challenges for automated metabolic reconstructions: Examples from the tetrahydrofolate synthesis pathway. *Comput Struct Biotechnol J* **2014**, *10*, 41-50.
136. Maden, B.E. Tetrahydrofolate and tetrahydromethanopterin compared: functionally distinct carriers in C1 metabolism. *Biochem. J.* **2000**, *350 Pt 3*, 609-629.
137. Falb, M.; Muller, K.; Konigsmaier, L.; Oberwinkler, T.; Horn, P.; von Gronau, S.; Gonzalez, O.; Pfeiffer, F.; Bornberg-Bauer, E.; Oesterhelt, D. Metabolism of halophilic archaea. *Extremophiles* **2008**, *12*, 177-196.
138. Grochowski, L.L.; Xu, H.; Leung, K.; White, R.H. Characterization of an Fe(2+)-dependent archaeal-specific GTP cyclohydrolase, MptA, from *Methanocaldococcus jannaschii*. *Biochemistry* **2007**, *46*, 6658-6667.
139. El Yacoubi, B.; Phillips, G.; Blaby, I.K.; Haas, C.E.; Cruz, Y.; Greenberg, J.; de Crecy-Lagard, V. A Gateway platform for functional genomics in *Haloferax volcanii*: deletion of three tRNA modification genes. *Archaea* **2009**, *2*, 211-219.
140. Scott, J.W.; Rasche, M.E. Purification, overproduction, and partial characterization of beta-RFAP synthase, a key enzyme in the methanopterin biosynthesis pathway. *J. Bacteriol.* **2002**, *184*, 4442-4448.
141. Dumitru, R.V.; Ragsdale, S.W. Mechanism of 4-(beta-D-ribofuranosyl)aminobenzene 5'-phosphate synthase, a key enzyme in the methanopterin biosynthetic pathway. *J. Biol. Chem.* **2004**, *279*, 39389-39395.
142. Vaupel, M.; Vorholt, J.A.; Thauer, R.K. Overproduction and one-step purification of the N5,N10-methenyltetrahydromethanopterin cyclohydrolase (Mch) from the hyperthermophilic *Methanopyrus kandleri*. *Extremophiles* **1998**, *2*, 15-22.
143. Shima, S.; Warkentin, E.; Grabarse, W.; Sordel, M.; Wicke, M.; Thauer, R.K.; Ermler, U. Structure of coenzyme F(420) dependent methylenetetrahydromethanopterin reductase from two methanogenic archaea. *J. Mol. Biol.* **2000**, *300*, 935-950.
144. Vaupel, M.; Thauer, R.K. Coenzyme F420-dependent N5,N10-methylenetetrahydromethanopterin reductase (Mer) from *Methanobacterium thermoautotrophicum* strain Marburg. Cloning, sequencing, transcriptional analysis, and functional expression in *Escherichia coli* of the *mer* gene. *Eur. J. Biochem.* **1995**, *231*, 773-778.
145. te Brömmelstroet, B.W.; Hensgens, C.M.; Keltjens, J.T.; van der Drift, C.; Vogels, G.D. Purification and properties of 5,10-methylenetetrahydromethanopterin reductase, a coenzyme F420-dependent enzyme, from *Methanobacterium thermoautotrophicum* strain delta H. *J. Biol. Chem.* **1990**, *265*, 1852-1857.
146. Kawai, S.; Mori, S.; Mukai, T.; Suzuki, S.; Yamada, T.; Hashimoto, W.; Murata, K. Inorganic Polyphosphate/ATP-NAD kinase of Micrococcus flavus and Mycobacterium tuberculosis H37Rv. *Biochem Biophys Res Commun* **2000**, *276*, 57-63.
147. Liu, J.; Lou, Y.; Yokota, H.; Adams, P.D.; Kim, R.; Kim, S.H. Crystal structures of an NAD kinase from *Archaeoglobus fulgidus* in complex with ATP, NAD, or NADP. *J. Mol. Biol.* **2005**, *354*, 289-303.
148. Zerulla, K.; Chimileski, S.; Nather, D.; Gophna, U.; Papke, R.T.; Soppa, J. DNA as a phosphate storage polymer and the alternative advantages of polyploidy for growth or survival. *PLoS One* **2014**, *9*, e94819.

149. Raffaelli, N.; Pisani, F.M.; Lorenzi, T.; Emanuelli, M.; Amici, A.; Ruggieri, S.; Magni, G. Characterization of nicotinamide mononucleotide adenylyltransferase from thermophilic archaea. *J. Bacteriol.* **1997**, *179*, 7718-7723.
150. Raffaelli, N.; Emanuelli, M.; Pisani, F.M.; Amici, A.; Lorenzi, T.; Ruggieri, S.; Magni, G. Identification of the archaeal NMN adenylyltransferase gene. *Mol. Cell. Biochem.* **1999**, *193*, 99-102.
151. Eustaquio, A.S.; Harle, J.; Noel, J.P.; Moore, B.S. S-Adenosyl-L-methionine hydrolase (adenosine-forming), a conserved bacterial and archaeal protein related to SAM-dependent halogenases. *ChemBioChem* **2008**, *9*, 2215-2219.
152. Deng, H.; McMahon, S.A.; Eustaquio, A.S.; Moore, B.S.; Naismith, J.H.; O'Hagan, D. Mechanistic insights into water activation in SAM hydroxide adenosyltransferase (duf-62). *ChemBioChem* **2009**, *10*, 2455-2459.
153. Rodionova, I.A.; Vetting, M.W.; Li, X.; Almo, S.C.; Osterman, A.L.; Rodionov, D.A. A novel bifunctional transcriptional regulator of riboflavin metabolism in Archaea. *Nucleic Acids Res.* **2017**, *45*, 3785-3799.
154. Haase, I.; Sarge, S.; Illarionov, B.; Laudert, D.; Hohmann, H.P.; Bacher, A.; Fischer, M. Enzymes from the haloacid dehalogenase (HAD) superfamily catalyse the elusive dephosphorylation step of riboflavin biosynthesis. *ChemBioChem* **2013**, *14*, 2272-2275.
155. London, N.; Farelli, J.D.; Brown, S.D.; Liu, C.; Huang, H.; Korczynska, M.; Al-Obaidi, N.F.; Babbitt, P.C.; Almo, S.C.; Allen, K.N., *et al.* Covalent docking predicts substrates for haloalkanoate dehalogenase superfamily phosphatases. *Biochemistry* **2015**, *54*, 528-537.
156. Sarge, S.; Haase, I.; Illarionov, B.; Laudert, D.; Hohmann, H.P.; Bacher, A.; Fischer, M. Catalysis of an essential step in vitamin B2 biosynthesis by a consortium of broad spectrum hydrolases. *ChemBioChem* **2015**, *16*, 2466-2469.
157. De Rosa, M.; Gambacorta, A. The lipids of archaebacteria. *Prog. Lipid Res.* **1988**, *27*, 153-175.
158. Dawson, K.S.; Freeman, K.H.; Macalady, J.L. Molecular characterization of core lipids from halophilic archaea grown under different salinity conditions. *Org. Geochem.* **2012**, *48*, 1-8.
159. De Rosa, M.; Gambacorta, A.; Nicolaus, B.; Ross, H.N.M.; Grant, W.D.; Bu'Lock, J.D. An asymmetric archaebacterial diether lipid from alkaliphilic halophiles. *Microbiology* **1982**, *128*, 343-348.
160. Tachibana, A. A novel prenyltransferase, farnesylgeranyl diphosphate synthase, from the haloalkaliphilic archaeon, *Natronobacterium pharaonis*. *FEBS Lett.* **1994**, *341*, 291-294.
161. Tachibana, A.; Yano, Y.; Otani, S.; Nomura, N.; Sako, Y.; Taniguchi, M. Novel prenyltransferase gene encoding farnesylgeranyl diphosphate synthase from a hyperthermophilic archaeon, *Aeropyrum pernix* - Molecular evolution with alteration in product specificity. *Eur. J. Biochem.* **2000**, *267*, 321-328.
162. Bale, N.J.; Sorokin, D.Y.; Hopmans, E.C.; Koenen, M.; Rijpstra, W.I.C.; Villanueva, L.; Wienk, H.; Sinninghe Damste, J.S. New insights into the polar lipid composition of extremely halo(alkali)philic euryarchaea from hypersaline lakes. *Front. Microbiol.* **2019**, *10*, 377.
163. Morii, H.; Koga, Y. CDP-2,3-Di-O-geranylgeranyl-sn-glycerol:L-serine O-archaetidyltransferase (archaetidylserine synthase) in the methanogenic archaeon *Methanothermobacter thermautotrophicus*. *J. Bacteriol.* **2003**, *185*, 1181-1189.

164. Daiyasu, H.; Kuma, K.; Yokoi, T.; Morii, H.; Koga, Y.; Toh, H. A study of archaeal enzymes involved in polar lipid synthesis linking amino acid sequence information, genomic contexts and lipid composition. *Archaea* **2005**, *1*, 399-410.
165. Abdul-Halim, M.F.; Schulze, S.; DiLucido, A.; Pfeiffer, F.; Bisson Filho, A.W.; Pohlschroder, M. Lipid anchoring of archaeosortase substrates and midcell growth in haloarchaea. *mBio* **2020**, *11*, e00349-00320.
166. Morii, H.; Kiyonari, S.; Ishino, Y.; Koga, Y. A novel biosynthetic pathway of archaetidyl-myo-inositol via archaetidyl-myo-inositol phosphate from CDP-archaeol and D-glucose 6-phosphate in methanoarchaeon *Methanothermobacter thermautotrophicus* cells. *J. Biol. Chem.* **2009**, *284*, 30766-30774.
167. Kates, M. The phytanyl ether-linked polar lipids and isoprenoid neutral lipids of extremely halophilic bacteria. *Prog. Chem. Fats Other Lipids* **1977**, *15*, 301-342.
168. Lombard, J.; Lopez-Garcia, P.; Moreira, D. Phylogenomic investigation of phospholipid synthesis in archaea. *Archaea* **2012**, *2012*, 630910.
169. Conover, R.K.; Doolittle, W.F. Characterization of a gene involved in histidine biosynthesis in *Halobacterium* (*Haloferax*) *volcanii*: isolation and rapid mapping by transformation of an auxotroph with cosmid DNA. *J. Bacteriol.* **1990**, *172*, 3244-3249.
170. Loc'h, J.; Blaud, M.; Rety, S.; Lebaron, S.; Deschamps, P.; Bareille, J.; Jombart, J.; Robert-Paganin, J.; Delbos, L.; Chardon, F., *et al.* RNA mimicry by the fap7 adenylate kinase in ribosome biogenesis. *PLoS Biol.* **2014**, *12*, e1001860.
171. Ren, H.; Wang, L.; Bennett, M.; Liang, Y.; Zheng, X.; Lu, F.; Li, L.; Nan, J.; Luo, M.; Eriksson, S., *et al.* The crystal structure of human adenylate kinase 6: An adenylate kinase localized to the cell nucleus. *Proc. Natl. Acad. Sci. U. S. A.* **2005**, *102*, 303-308.
172. Sprott, G.D.; Larocque, S.; Cadotte, N.; Dicaire, C.J.; McGee, M.; Brisson, J.R. Novel polar lipids of halophilic eubacterium *Planococcus* H8 and archaeon *Haloferax volcanii*. *Biochim Biophys Acta* **2003**, *1633*, 179-188.
173. Chamovitz, D.; Misawa, N.; Sandmann, G.; Hirschberg, J. Molecular cloning and expression in *Escherichia coli* of a cyanobacterial gene coding for phytoene synthase, a carotenoid biosynthesis enzyme. *FEBS Lett.* **1992**, *296*, 305-310.
174. Maurer, S.; Ludt, K.; Soppa, J. Characterization of copy number control of two *Haloferax volcanii* replication origins using deletion mutants and haloarchaeal artificial chromosomes. *J. Bacteriol.* **2018**, *200*, e00517-00517.
175. Liu, H.; Han, J.; Liu, X.; Zhou, J.; Xiang, H. Development of *pyrF*-based gene knockout systems for genome-wide manipulation of the archaea *Haloferax mediterranei* and *Haloarcula hispanica*. *J Genet Genomics* **2011**, *38*, 261-269.
176. Yang, Y.; Yatsunami, R.; Ando, A.; Miyoko, N.; Fukui, T.; Takaichi, S.; Nakamura, S. Complete biosynthetic pathway of the C50 carotenoid bacterioruberin from lycopene in the extremely halophilic archaeon *Haloarcula japonica*. *J. Bacteriol.* **2015**, *197*, 1614-1623.
177. Dummer, A.M.; Bonsall, J.C.; Cihla, J.B.; Lawry, S.M.; Johnson, G.C.; Peck, R.F. Bacterioopsin-mediated regulation of bacterioruberin biosynthesis in *Halobacterium salinarum*. *J. Bacteriol.* **2011**, *193*, 5658-5667.
178. Giani, M.; Miralles-Robledillo, J.M.; Peiro, G.; Pire, C.; Martinez-Espinosa, R.M. Deciphering pathways for carotenogenesis in haloarchaea. *Molecules* **2020**, *25*, 1197.
179. Elling, F.J.; Becker, K.W.; Konneke, M.; Schroder, J.M.; Kellermann, M.Y.; Thomm, M.; Hinrichs, K.U. Respiratory quinones in Archaea: phylogenetic distribution and

application as biomarkers in the marine environment. *Environ. Microbiol.* **2016**, *18*, 692-707.
180. Kellermann, M.Y.; Yoshinaga, M.Y.; Valentine, R.C.; Wormer, L.; Valentine, D.L. Important roles for membrane lipids in haloarchaeal bioenergetics. *Biochim Biophys Acta* **2016**, *1858*, 2940-2956.
181. Leffers, H.; Gropp, F.; Lottspeich, F.; Zillig, W.; Garrett, R.A. Sequence, organization, transcription and evolution of RNA polymerase subunit genes from the archaebacterial extreme halophiles *Halobacterium halobium* and *Halococcus morrhuae*. *J. Mol. Biol.* **1989**, *206*, 1-17.
182. Madon, J.; Zillig, W. A form of the DNA-dependent RNA polymerase of *Halobacterium halobium*, containing an additional component, is able to transcribe native DNA. *Eur. J. Biochem.* **1983**, *133*, 471-474.
183. Madon, J.; Leser, U.; Zillig, W. DNA-dependent RNA polymerase from the extremely halophilic archaebacterium *Halococcus morrhuae*. *Eur. J. Biochem.* **1983**, *135*, 279-283.
184. Rivlin, A.A.; Chan, Y.L.; Wool, I.G. The contribution of a zinc finger motif to the function of yeast ribosomal protein YL37a. *J. Mol. Biol.* **1999**, *294*, 909-919.
185. Dresios, J.; Chan, Y.L.; Wool, I.G. The role of the zinc finger motif and of the residues at the amino terminus in the function of yeast ribosomal protein YL37a. *J. Mol. Biol.* **2002**, *316*, 475-488.
186. Ban, N.; Nissen, P.; Hansen, J.; Moore, P.B.; Steitz, T.A. The complete atomic structure of the large ribosomal subunit at 2.4 A resolution. *Science* **2000**, *289*, 905-920.
187. Klein, C.; Aivaliotis, M.; Olsen, J.V.; Falb, M.; Besir, H.; Scheffer, B.; Bisle, B.; Tebbe, A.; Konstantinidis, K.; Siedler, F., *et al.* The low molecular weight proteome of *Halobacterium salinarum*. *J. Proteome Res.* **2007**, *6*, 1510-1518.
188. Tarasov, V.Y.; Besir, H.; Schwaiger, R.; Klee, K.; Furtwangler, K.; Pfeiffer, F.; Oesterhelt, D. A small protein from the *bop-brp* intergenic region of *Halobacterium salinarum* contains a zinc finger motif and regulates *bop* and *crtB1* transcription. *Mol. Microbiol.* **2008**, *67*, 772-780.
189. Nagel, C.; Machulla, A.; Zahn, S.; Soppa, J. Several one-domain zinc finger micro-proteins of *Haloferax volcanii* are important for stress adaptation, biofilm formation, and swarming. *Genes (Basel)* **2019**, *10*, 361.
190. Pedruzzi, I.; Rivoire, C.; Auchincloss, A.H.; Coudert, E.; Keller, G.; de Castro, E.; Baratin, D.; Cuche, B.A.; Bougueleret, L.; Poux, S., *et al.* HAMAP in 2015: updates to the protein family classification and annotation system. *Nucleic Acids Res.* **2015**, *43*, D1064-1070.
191. Zhu, X.; Dzikovski, B.; Su, X.; Torelli, A.T.; Zhang, Y.; Ealick, S.E.; Freed, J.H.; Lin, H. Mechanistic understanding of *Pyrococcus horikoshii* Dph2, a [4Fe-4S] enzyme required for diphthamide biosynthesis. *Mol. Biosyst.* **2011**, *7*, 74-81.
192. Zhu, X.; Kim, J.; Su, X.; Lin, H. Reconstitution of diphthine synthase activity in vitro. *Biochemistry* **2010**, *49*, 9649-9657.
193. de Crecy-Lagard, V.; Forouhar, F.; Brochier-Armanet, C.; Tong, L.; Hunt, J.F. Comparative genomic analysis of the DUF71/COG2102 family predicts roles in diphthamide biosynthesis and B12 salvage. *Biol. Direct* **2012**, *7*, 32.
194. Su, X.; Lin, Z.; Chen, W.; Jiang, H.; Zhang, S.; Lin, H. Chemogenomic approach identified yeast YLR143W as diphthamide synthetase. *Proc. Natl. Acad. Sci. U. S. A.* **2012**, *109*, 19983-19987.

195. Uthman, S.; Bar, C.; Scheidt, V.; Liu, S.; ten Have, S.; Giorgini, F.; Stark, M.J.; Schaffrath, R. The amidation step of diphthamide biosynthesis in yeast requires DPH6, a gene identified through mining the DPH1-DPH5 interaction network. *PLoS Genet.* **2013**, *9*, e1003334.
196. Ng, S.Y.; Chaban, B.; VanDyke, D.J.; Jarrell, K.F. Archaeal signal peptidases. *Microbiology (Reading)* **2007**, *153*, 305-314.
197. Raut, P.; Glass, J.B.; Lieberman, R.L. Archaeal roots of intramembrane aspartyl protease siblings signal peptide peptidase and presenilin. *Proteins* **2021**, *89*, 232-241.
198. Rangaswamy, V.; Altekar, W. Ketohexokinase (ATP:D-fructose 1-phosphotransferase) from a halophilic archaebacterium, *Haloarcula vallismortis*: purification and properties. *J. Bacteriol.* **1994**, *176*, 5505-5512.
199. Anderson, I.; Scheuner, C.; Goker, M.; Mavromatis, K.; Hooper, S.D.; Porat, I.; Klenk, H.P.; Ivanova, N.; Kyrpides, N. Novel insights into the diversity of catabolic metabolism from ten haloarchaeal genomes. *PLoS One* **2011**, *6*, e20237.
200. Williams, T.J.; Allen, M.A.; Liao, Y.; Raftery, M.J.; Cavicchioli, R. Sucrose metabolism in haloarchaea: reassessment using genomics, proteomics, and metagenomics. *Appl Environ Microbiol* **2019**, *85*, e02935-02918.
201. Aulkemeyer, P.; Ebner, R.; Heilenmann, G.; Jahreis, K.; Schmid, K.; Wrieden, S.; Lengeler, J.W. Molecular analysis of two fructokinases involved in sucrose metabolism of enteric bacteria. *Mol. Microbiol.* **1991**, *5*, 2913-2922.
202. Qu, Q.; Lee, S.J.; Boos, W. Molecular and biochemical characterization of a fructose-6-phosphate-forming and ATP-dependent fructokinase of the hyperthermophilic archaeon *Thermococcus litoralis*. *Extremophiles* **2004**, *8*, 301-308.
203. Chaga, G.; Porath, J.; Illeni, T. Isolation and purification of amyloglucosidase from *Halobacterium sodomense*. *Biomed. Chromatogr.* **1993**, *7*, 256-261.
204. Ohnishi, H.; Kitamura, H.; Minowa, T.; Sakai, H.; Ohta, T. Molecular cloning of a glucoamylase gene from a thermophilic *Clostridium* and kinetics of the cloned enzyme. *Eur. J. Biochem.* **1992**, *207*, 413-418.
205. Rudolph, B.; Hansen, T.; Schonheit, P. Glucose-6-phosphate isomerase from the hyperthermophilic archaeon *Methanococcus jannaschii*: characterization of the first archaeal member of the phosphoglucose isomerase superfamily. *Arch. Microbiol.* **2004**, *181*, 82-87.
206. Ahmed, H.; Ettema, T.J.; Tjaden, B.; Geerling, A.C.; van der Oost, J.; Siebers, B. The semi-phosphorylative Entner-Doudoroff pathway in hyperthermophilic archaea: a re-evaluation. *Biochem. J.* **2005**, *390*, 529-540.
207. Chai, Y.; Kolter, R.; Losick, R. A widely conserved gene cluster required for lactate utilization in *Bacillus subtilis* and its involvement in biofilm formation. *J. Bacteriol.* **2009**, *191*, 2423-2430.
208. Gao, C.; Wang, Y.; Zhang, Y.; Lv, M.; Dou, P.; Xu, P.; Ma, C. NAD-independent L-lactate dehydrogenase required for L-lactate utilization in *Pseudomonas stutzeri* A1501. *J. Bacteriol.* **2015**, *197*, 2239-2247.
209. Pfrimer, P.; de Moraes, L.M.; Galdino, A.S.; Salles, L.P.; Reis, V.C.; De Marco, J.L.; Prates, M.V.; Bloch, C., Jr.; Torres, F.A. Cloning, purification, and partial characterization of *Bacillus subtilis* urate oxidase expressed in *Escherichia coli*. *J. Biomed. Biotechnol.* **2010**, *2010*, 674908.

210. Kim, K.; Park, J.; Rhee, S. Structural and functional basis for (S)-allantoin formation in the ureide pathway. *J. Biol. Chem.* **2007**, *282*, 23457-23464.
211. Lee, Y.; Lee, D.H.; Kho, C.W.; Lee, A.Y.; Jang, M.; Cho, S.; Lee, C.H.; Lee, J.S.; Myung, P.K.; Park, B.C., *et al.* Transthyretin-related proteins function to facilitate the hydrolysis of 5-hydroxyisourate, the end product of the uricase reaction. *FEBS Lett.* **2005**, *579*, 4769-4774.
212. Schultz, A.C.; Nygaard, P.; Saxild, H.H. Functional analysis of 14 genes that constitute the purine catabolic pathway in *Bacillus subtilis* and evidence for a novel regulon controlled by the PucR transcription activator. *J. Bacteriol.* **2001**, *183*, 3293-3302.
213. Ho, Y.Y.; Huang, Y.H.; Huang, C.Y. Chemical rescue of the post-translationally carboxylated lysine mutant of allantoinase and dihydroorotase by metal ions and short-chain carboxylic acids. *Amino Acids* **2013**, *44*, 1181-1191.
214. Xu, Z.; Jiang, W.H.; Jiao, R.S.; Yang, Y.L. [Cloning, sequencing and high expression in *Escherichia coli* of D-hydantoinase gene from *Burkholderia pickettii*]. *Sheng Wu Gong Cheng Xue Bao* **2002**, *18*, 149-154.
215. Barba, M.; Dutoit, R.; Legrain, C.; Labedan, B. Identifying reaction modules in metabolic pathways: bioinformatic deduction and experimental validation of a new putative route in purine catabolism. *BMC Syst. Biol.* **2013**, *7*, 99.
216. Werner, A.K.; Romeis, T.; Witte, C.P. Ureide catabolism in *Arabidopsis thaliana* and *Escherichia coli*. *Nat. Chem. Biol.* **2010**, *6*, 19-21.
217. Werner, A.K.; Medina-Escobar, N.; Zulawski, M.; Sparkes, I.A.; Cao, F.Q.; Witte, C.P. The ureide-degrading reactions of purine ring catabolism employ three amidohydrolases and one aminohydrolase in *Arabidopsis*, soybean, and rice. *Plant Physiol.* **2013**, *163*, 672-681.
218. Martinez-Rodriguez, S.; Garcia-Pino, A.; Las Heras-Vazquez, F.J.; Clemente-Jimenez, J.M.; Rodriguez-Vico, F.; Garcia-Ruiz, J.M.; Loris, R.; Gavira, J.A. Mutational and structural analysis of L-N-carbamoylase reveals new insights into a peptidase M20/M25/M40 family member. *J. Bacteriol.* **2012**, *194*, 5759-5768.
219. Xi, H.; Schneider, B.L.; Reitzer, L. Purine catabolism in *Escherichia coli* and function of xanthine dehydrogenase in purine salvage. *J. Bacteriol.* **2000**, *182*, 5332-5341.
220. Stuerlauridsen, B.; Nygaard, P. Purine salvage in two halophilic archaea - characterization of salvage pathways and isolation of mutants resistant to purine analogs. *J. Bacteriol.* **1998**, *180*, 457-463.
221. Karatza, P.; Frillingos, S. Cloning and functional characterization of two bacterial members of the NAT/NCS2 family in *Escherichia coli*. *Mol. Membr. Biol.* **2005**, *22*, 251-261.
222. Desguin, B.; Soumillion, P.; Hols, P.; Hausinger, R.P. Nickel-pincer cofactor biosynthesis involves LarB-catalyzed pyridinium carboxylation and LarE-dependent sacrificial sulfur insertion. *Proc. Natl. Acad. Sci. U. S. A.* **2016**, *113*, 5598-5603.
223. Desguin, B.; Soumillion, P.; Hausinger, R.P.; Hols, P. Unexpected complexity in the lactate racemization system of lactic acid bacteria. *FEMS Microbiol. Rev.* **2017**, *41*, S71-S83.
224. Hausinger, R.P.; Desguin, B.; Fellner, M.; Rankin, J.A.; Hu, J. Nickel-pincer nucleotide cofactor. *Curr. Opin. Chem. Biol.* **2018**, *47*, 18-23.
225. Corrigan, R.M.; Grundling, A. Cyclic di-AMP: another second messenger enters the fray. *Nat. Rev. Microbiol.* **2013**, *11*, 513-524.

226. Braun, F.; Thomalla, L.; van der Does, C.; Quax, T.E.F.; Allers, T.; Kaever, V.; Albers, S.V. Cyclic nucleotides in archaea: Cyclic di-AMP in the archaeon *Haloferax volcanii* and its putative role. *Microbiologyopen* **2019**, *8*, e00829.
227. Gundlach, J.; Mehne, F.M.; Herzberg, C.; Kampf, J.; Valerius, O.; Kaever, V.; Stulke, J. An essential poison: synthesis and degradation of cyclic di-AMP in *Bacillus subtilis*. *J. Bacteriol.* **2015**, *197*, 3265-3274.
228. Commichau, F.M.; Heidemann, J.L.; Ficner, R.; Stulke, J. Making and breaking of an essential poison: the cyclases and phosphodiesterases that produce and degrade the essential second messenger cyclic di-AMP in bacteria. *J. Bacteriol.* **2019**, *201*, e00462-00418.
229. Yin, W.; Cai, X.; Ma, H.; Zhu, L.; Zhang, Y.; Chou, S.H.; Galperin, M.Y.; He, J. A decade of research on the second messenger c-di-AMP. *FEMS Microbiol. Rev.* **2020**, *44*, 701-724.
230. He, J.; Yin, W.; Galperin, M.Y.; Chou, S.H. Cyclic di-AMP, a second messenger of primary importance: tertiary structures and binding mechanisms. *Nucleic Acids Res.* **2020**, *48*, 2807-2829.
231. Fischer, S.; John von Freyend, S.; Sabag-Daigle, A.; Daniels, C.J.; Allers, T.; Marchfelder, A. Assigning a function to a conserved archaeal metallo-beta-lactamase from *Haloferax volcanii*. *Extremophiles* **2012**, *16*, 333-343.
232. Kaminski, L.; Guan, Z.; Yurist-Doutsch, S.; Eichler, J. Two distinct N-glycosylation pathways process the *Haloferax volcanii* S-layer glycoprotein upon changes in environmental salinity. *mBio* **2013**, *4*, e00716-00713.
233. Desmarais, J.J.; Flamholz, A.I.; Blikstad, C.; Dugan, E.J.; Laughlin, T.G.; Oltrogge, L.M.; Chen, A.W.; Wetmore, K.; Diamond, S.; Wang, J.Y., *et al.* DABs are inorganic carbon pumps found throughout prokaryotic phyla. *Nat Microbiol* **2019**, *4*, 2204-2215.
